# An Enhanced Slime Mould Algorithm Based on Best–Worst Management for Numerical Optimization Problems

**DOI:** 10.3390/biomimetics10080504

**Published:** 2025-08-01

**Authors:** Tongzheng Li, Hongchi Meng, Dong Wang, Bin Fu, Yuanyuan Shao, Zhenzhong Liu

**Affiliations:** 1 Salford Business School, University of Salford, Manchester M5 4WT, UK; ltz@asu.edu.pl; 2 ESC Amiens, 80000 Amiens, France; abc15252093366@163.com; 3 School of Management, Fudan University, Shanghai 200433, China; w116826@163.com; 4 Taizhou Institute of Zhejiang University, Taizhou 318000, China; fubin@tzizju.cn (B.F.); shaoyuanyuan@tzizju.cn (Y.S.)

**Keywords:** slime mold algorithm, swarm intelligence, greedy mechanism, stagnant replacement mechanism, metaheuristic algorithms

## Abstract

The Slime Mould Algorithm (SMA) is a widely used swarm intelligence algorithm. Encouraged by the theory of no free lunch and the inherent shortcomings of the SMA, this work proposes a new variant of the SMA, called the BWSMA, in which three improvement mechanisms are integrated. The adaptive greedy mechanism is used to accelerate the convergence of the algorithm and avoid ineffective updates. The best–worst management strategy improves the quality of the population and increases its search capability. The stagnant replacement mechanism prevents the algorithm from falling into a local optimum by replacing stalled individuals. In order to verify the effectiveness of the proposed method, this paper conducts a full range of experiments on the CEC2018 test suite and the CEC2022 test suite and compares BWSMA with three derived algorithms, eight SMA variants, and eight other improved algorithms. The experimental results are analyzed using the Wilcoxon rank-sum test, the Friedman test, and the Nemenyi test. The results indicate that the BWSMA significantly outperforms these compared algorithms. In the comparison with the SMA variants, the BWSMA obtained average rankings of 1.414, 1.138, 1.069, and 1.414. In comparison with other improved algorithms, the BWSMA obtained average rankings of 2.583 and 1.833. Finally, the applicability of the BWSMA is further validated through two structural optimization problems. In conclusion, the proposed BWSMA is a promising algorithm with excellent search accuracy and robustness.

## 1. Introduction

In today’s world, the difficulty of optimization problems is increasing with the rapid development of technology and the increasing complexity of engineering applications [1]. These complex problems usually have multiple local optimal solutions, high dimensionality, and complex coupling relationships, which bring unprecedented challenges to the traditional solution methods [1]. To cope with these difficulties, optimization algorithms have become a hot research topic. Optimization algorithms are mainly divided into two categories: deterministic algorithms and nondeterministic algorithms [2]. Deterministic algorithms generate consistent outputs with the same inputs, and they rely on a rigorous computational process to find exact solutions [3]. However, such algorithms often take too long to run due to excessive computation when dealing with large-scale data. In addition, when faced with complex and variable high-dimensional problems, deterministic algorithms are prone to fall into local optimal solutions and exhibit low adaptability. In contrast, metaheuristic algorithms among the nondeterministic algorithms stand out with their unique advantages. Metaheuristic algorithms do not rely on the derivative information of the problem and are able to find approximate optimal solutions quickly in complex and variable environments [4]. Such algorithms are widely adopted in several fields, such as task planning [5,6,7], image processing [8,9,10], parameter tuning [11,12,13], feature selection [14,15,16], energy management [17,18,19], biomedical detection [20,21,22], electronic monitoring systems [23,24], model calibration [25,26], etc.

These algorithms do have certain drawbacks, though. When dealing with difficult problems, they have a tendency to converge slowly, lack stability, and fall into local optimum. Researchers have been investigating novel metaheuristic algorithms and enhancement techniques in an effort to get beyond these restrictions. Numerous innovative algorithms have been put forth, drawing influence from a variety of disciplines, including physics, sociology, and biology. To enhance their effectiveness, these algorithms mimic social behaviors and natural events in an effort to strike a better balance between exploration and exploitation. Evolution-based algorithms (EAs), physics-based algorithms (PAs), human-based algorithms (HAs), and swarm intelligence algorithms (SIAs) are the four basic categories into which metaheuristic algorithms can be generally divided.

**Figure 1:**
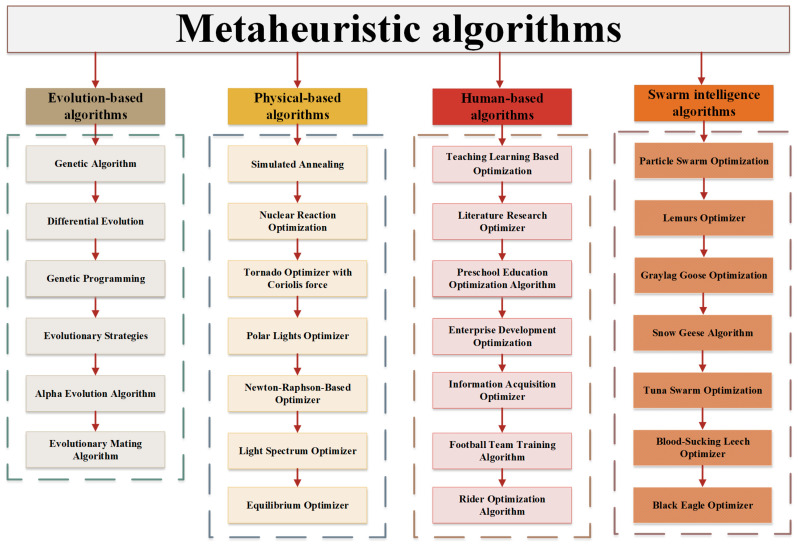
Summary of metaheuristic algorithms.

The Genetic Algorithm (GA) [27] and Differential Evolution (DE) [28] are designed based on the idea of evolution, including selection, mutation, and crossover, and we categorize them as EAs. Apart from these, there are some other algorithms based on evolutionary ideas, such as Genetic Programming (GP) [29], Evolutionary Strategies (ESs) [30], Alpha Evolution Algorithm (AEA) [31], and Evolutionary Mating Algorithm (EMA) [32]. PAs include Simulated Annealing (SA) [33], Nuclear Reaction Optimization (NRO) [34], Tornado Optimizer with Coriolis force (TOC) [35], Polar Lights Optimizer (PLO) [36], Newton–Raphson-Based Optimizer (NRBO) [37], Light Spectrum Optimizer (LSO) [38], Equilibrium Optimizer (EO) [39], Attraction–Repulsion Optimization Algorithm (AROA) [40], Henry Gas Solubility Optimization (HGSO) [41], Prism Refraction Search (PRS) [42], and others. It has encompassed Teaching–Learning-Based Optimization (TLBO) [43], Literature Research Optimizer (LRO) [44], Preschool Education Optimization Algorithm (PEOA) [45], Enterprise Development Optimization (EDO) [46], Information Acquisition Optimizer (IAO) [47], Football Team Training Algorithm (FTTA) [48], Rider Optimization Algorithm (ROA) [49], Escape Optimization Algorithm (EOA) [50], Kids Learning Optimizer (KLO) [51], Human Memory Optimization Algorithm (HMOA) [52], and similar approaches. SIAs are one of the largest groups of metaheuristic algorithms. Algorithms such as Particle Swarm Optimization (PSO) [53], Lemurs Optimizer (LO) [54], Graylag Goose Optimization (GGO) [55], Snow Geese Algorithm (SGA) [56], Tuna Swarm Optimization (TSO) [1], Blood-Sucking Leech Optimizer (BSLO) [57], Black Eagle Optimizer (BEO) [58], Elk Herd Optimizer (EHO) [59], Sled Dog Optimizer (SDO) [60], and Electric Eel Foraging Optimization (EEFO) [61], which mimic the behaviors of various swarming groups in nature, are classified as SIAs. These four categories of metaheuristic algorithms are summarized in Figure 1.

In 2020, Li et al. [57] introduced the Slime Mould method (SMA), a swarm intelligence method. The SMA primarily mimics slime molds’ feeding habits and morphological changes. The SMA has been widely used in various optimization sectors because to its exceptional performance. Like many other MAs, the SMA has been successful in certain problem areas, but it is important to understand its limitations. Furthermore, no algorithm can offer the most suitable response in any particular domain, according to the “no free lunch” (NFL) concept [58]. It is vital to enhance the SMA in order to increase its performance and expand their applications. Researchers have attempted in several ways to remedy the SMA’s limitations in recent years. Ornek et al. incorporated the sine–cosine algorithm in order to improve the search capability of the SMA [62]. To solve the PV cell model parameter estimation, Mostafa et al. used adaptive weights to dynamically adjust the search behavior of slime molds [63]. Yu et al. further refined the biological mechanisms of the SMA by adding pole growth, memory, and amoebic mechanisms to improve the overall performance of the SMA [64]. Chen et al. introduced the arithmetic optimization algorithm into the SMA for improving its exploitation and integrated the mutation strategy and restart strategy to help the SMA jump out of the local optimum [65]. Alhashash et al. developed a new SMA variant that utilizes an adaptive intelligent switching mechanism to dynamically select different search operators [66]. Conteh et al. proposed a triangular variation rule and a good point set initialization strategy for addressing the shortcomings of poor diversity in SMA populations [67]. In order to solve the multi-UAV path-planning problem, Ma et al. proposed an adaptive improved SMA to enrich the population diversity by integrating an adaptive switching operator and an adaptive perturbation strategy [68]. Focusing on improving the initial population quality of the SMA, Duan et al. proposed an elite population initialization strategy based on Bloch balls and dynamically adjusted the step size using an adaptive search operator to improve the search efficiency [69]. With the review of the above SMA variants, we observe that the SMA suffers from the deficiencies of unbalanced exploitation and exploration, insufficient population diversity, and difficulty in escaping from the trap of local optimum. Although these variants solve the deficiencies of the SMA to some extent, there is still room for further improvement of the SMA.

In this work, an SMA variant integrating the adaptive greedy mechanism (AGM), best–worst management strategy (BWS), and stagnant replacement mechanism (SRM) is proposed, which is called the BWSMA. The AGM can dynamically adjust the execution of the greedy mechanism or not, which simultaneously achieves fast convergence and avoids population clustering. The BWS strategy improves the quality of the superior and inferior groups and promotes the balance between global and local search. The SRM strategy reduces the probability of the SMA falling into a local optimum by updating stagnant individuals. In order to fully assess the performance of the BWSMA, we conducted three experiments. First, we evaluated the impact of the proposed three strategies on the BWSMA based on the CEC2018 and CEC2022 test sets. Then, we compared the performance of the BWSMA and other SMA variants on the CEC2018 test set. Finally, to further validate the performance of the BWSMA, we compare it with other state-of-the-art metaheuristics on the CEC2022 test set. The experimental results indicate that the BWSMA performs well on these benchmark function problems, demonstrating its potential for solving a wide range of complex problems. The contributions and highlights of this paper are as follows:

(1) The AGM mechanism effectively enhances the exploitation of the SMA and improves its convergence speed while enriching the population diversity.
(2) The BWS enhances the global exploration ability of the algorithm by utilizing the information of the dominant group to guide the population towards high-quality regions, and the introduction of dominant individuals enhances the ability of the SMA to utilize local regions and achieve better convergence to the target solution.
(3) The SRM prevents the population from falling into search stagnation.
(4) The superior performance of the BWSMA is verified by comparing it with eight SMA variants and eight state-of-the-art improved algorithms on the CEC2018 and CEC2022 test sets.
(5) The applicability of the BWSMA is verified in two engineering constrained-optimization problems.

The remainder of this work is structured as follows: Section 2 describes the principles of the basic SMA. Section 3 elaborates on the proposed BWSMA, providing flowcharts, pseudo-code, and time complexity analysis. In Section 4, the BWSMA is thoroughly examined, and a detailed analysis is provided. In Section 5, the BWSMA is examined for its applicability in engineering constrained-optimization problems. Finally, Section 6 summarizes the results of related work and provides an outlook on future research directions.

## 2. Overview of Slime Mould Algorithm

The basic SMA models the algorithm by simulating the foraging behavior and morphological changes of slime molds. The SMA first calculates the concentration of each food item, and then after determining the location of the food item, the slime molds keep approaching and surrounding the food item. Finally, the operation of grasping the food is executed. The specific model of the SMA is represented as follows:

### 2.1. Population Initialization

As a swarm intelligence algorithm, the SMA adopts the same method as other swarm intelligence algorithms when initializing the population. That is, N individuals are randomly generated in a D-dimensional space with a lower limit of lb and an upper limit of ub, as shown in Equation (1):

(1) $$P_{i}^{ini}=rand\times (ub-lb)+lb,\;\;i=1,2,\ldots ,N$$

where $P_{i}^{ini}$ denotes the initial position of the $i^{th}$ individual, and rand is a random vector in the range [0, 1].

### 2.2. Position Updating

The SMA has a total of three different updating methods in the position updating phase, and the decision of which method to use is based on the values of the parameters $p_{i}$ and z. The three methods of updating the SMA are shown in Equations (2)–(4):

(2) $$P_{i}^{t+1}=rand\times (ub-lb)+lb,\;rand<z$$

(3) $$P_{i}^{t+1}=P_{best}+vb\times (W\times P_{a}^{t}-P_{b}^{t}),\;rand\geq z\;\mathrm{and}\;rand<p_{i}$$

(4) $$P_{i}^{t+1}=vc\times P_{i}^{t},\;rand\geq z\;\mathrm{and}\;rand\geq p_{i}$$

where z is a constant value, which is taken to be 0.03 in the SMA source literature. $p_{i}$ is calculated from the fitness of the individual itself and the fitness of the best global individual, as shown in Equation (5). $P_{i}^{t+1}$ is the position of the $i^{th}$ individual at iteration t+1. $P_{best}$ is the position of the global optimal individual. $P_{a}^{t}$ and $P_{b}^{t}$ are two different individuals randomly selected from the current slime mold population. vb and vc are two *D*-dimensional vectors, where vb is in the range $\left[-c,c\right]$ and vc is in the range $\left[-d,d\right]$. c and d are defined by Equations (6) and (7). W is a weighting factor, as shown in Equation (8).

(5) $$p_{i}=\tanh\left|F\left(P_{i}^{t}\right)-F\left(P_{best}\right)\right|$$

(6) c=arctanh(−(t/T)+1)

(7) d=1−t/T

(8) $$W(smellindex(i))=\left\{\begin{array}{l} 1+rand\times \log(\frac{F\left(P_{b}^{t}\right)-F\left(P_{i}^{t}\right)}{F\left(P_{b}^{t}\right)-F\left(P_{w}^{t}\right)}),condition\\ 1-rand\times \log(\frac{F\left(P_{b}^{t}\right)-F\left(P_{i}^{t}\right)}{F\left(P_{b}^{t}\right)-F\left(P_{w}^{t}\right)}),others \end{array}\right.$$

(9) $$smellindex=sort(F\left(X^{t}\right))$$

Here, $F\left(P_{i}^{t}\right)$ denotes the fitness of the $i^{th}$ individual. $F\left(P_{best}\right)$ is the fitness of the best individual. T denotes the maximum number of iterations. $P_{b}^{t}$ and $P_{w}^{t}$ are the best and worst individuals of the current population. smellindex represents a sorted array of fitness values derived from sorting $F\left(X^{t}\right)$. condition denotes individuals ranked in the top half of fitness values.

## 3. Framework of the Proposed BWSMA

In order to solve the concerns of the SMA such as slow convergence speed, low accuracy, and easy to fall into local optimum, we propose an SMA variant called the BWSMA. The proposed BWSMA is integrated with three improvement techniques on the basis of the SMA: adaptive greedy mechanism (AGM), best–worst management strategy (BWS), and stagnant replacement mechanism (SRM). In this section, we introduce the above three improvement mechanisms in turn and provide the pseudo-code, flowchart, and time complexity analysis of the proposed BWSMA.

### 3.1. Adaptive Greedy Mechanism

In the basic SMA, all individuals retain the newest individual directly after updating, regardless of whether it gets better or worse. This inheritance relationship between parent and child individuals is not conducive to the convergence of the algorithm. Greedy selection is a simple and efficient mechanism that requires each individual to judge the goodness or badness of the new individual after each update and then decide whether to use the new individual or not. This inheritance mechanism accelerates the convergence of the algorithm. However, the greedy mechanism is also problematic. A focus on keeping better offspring individuals tends to lead to clustering of the population, which is not conducive to exploring a wider area. At the same time, the offspring that did not get better may get better in the next iteration, but we abandon it using the greedy mechanism. Therefore, we cannot use the greedy mechanism for all of them, nor can we ignore the good and bad and keep them directly. For this reason, this paper proposes an adaptive greedy mechanism (AGM). In the AGM, we decide which approach to use by judging the current number of individuals that have become good by choosing the greedy strategy and by retaining them directly. For each individual, we determine the size of the switching parameters S and rand, and when *rand* < S, the individual uses the greedy mechanism. Conversely, the individual simply keeps the latest position. S is obtained from Equation (10).

(10) $$S^{t+1}=S^{t}+0.05\times \left(1-S^{t}\right)\times \left(\frac{GS_{better}}{GS}-\frac{KD_{better}}{KD}\right)\times t/T$$

Here, GS is the number of individuals that select the greedy mechanism. KD is the number of individuals that directly keep the newest position. $GS_{better}$ is the number of offspring individuals that outperform the parent individuals after selecting the greedy mechanism. $KD_{better}$ is the number of individuals that actually become better by directly keeping the newest position.

### 3.2. Best–Worst Management Strategy

In the metaheuristic algorithm, the superior individuals represent the current promising search direction, and the inferior individuals represent the current search region with no prospect. However, superior individuals may guide the population to gradually fall into the local optimum trap, and inferior individuals are instead able to expand the search area. This is because the exploration phase is only a rough search of large areas, and the exploitation phase is a precise search in the search areas determined in the previous phase, and these large areas may not contain optimal locations, but instead, optimal locations may exist in areas that were considered hopeless in the previous phase. Therefore, it is necessary to utilize the characteristics of different groups to guide the population search. To this end, this paper proposes a best–worst management strategy (BWS). In the BWS, we divide the population into three subpopulations according to fitness: the superior group, the balanced group, and the inferior group. For the $N_{1}$ individuals with better fitness, we require them to have the best convergence ability while spacing the local optimal avoidance ability. For the $N_{2}$ individuals with poor adaptation, we want them to maximize the search range. For the middle part of the individuals, we let the dynamic selection be developed and explored, i.e., the original SMA update formula. The updating methods for the superior and inferior individuals are shown below:

(11) $$\mathrm{Superior}\;\mathrm{Group}:\;P_{i}^{t+1}=\frac{\left(P_{i}^{t}+P_{w}+P_{e}\right)}{3}+g_{i},g_{i}\sim N\left(0,C\right)$$

(12) $$\mathrm{inferior}\;\mathrm{Group}:\;P_{i}^{t+1}=P_{w}+g_{i},g_{i}\sim N\left(0,C\right)$$

(13) $$C=\frac{1}{\left|Q_{d}\right|}\sum_{i=1}^{\left|Q_{d}\right|}\left(P_{i}^{Q}-P_{w}\right)\times {\left(P_{i}^{Q}-P_{w}\right)}^{T},P_{i}^{Q}\in Q_{d}$$

(14) $$P_{w}=\sum_{i=1}^{\left|Q_{d}\right|}\ln\left(\left|Q_{d}\right|+1\right)/\left(\sum_{i=1}^{\left|Q_{d}\right|}\left(\ln\left(\left|Q_{d}\right|+1\right)-\ln\left(i\right)\right)\right)\times P_{i}^{Q},P_{i}^{Q}\in Q_{d}$$

where $P_{w}$ is the weighted average position of the best individuals. Replacing the arithmetic average with a weighted average better captures the contribution of each individual to the search trend. $P_{e}$ is a randomly selected individual from the top three individuals in terms of fitness, inspired by the Gray Wolf Optimizer [70]. Different superior individuals can correct the search direction and enrich the population diversity. $Q_{d}$ is the set of dominant groups, consisting of the top half of individuals at each iteration, and controls the size of the set according to the first-in-first-out principle.

### 3.3. Stagnant Replacement Mechanism

The SMA achieves positional reset for some individuals through Equation (2), which somewhat overcomes the ease of falling into localized traps. However, this approach is too blind, and resetting without timing risks resetting good individuals, which, in turn, slows down convergence. In order to effectively overcome the stagnation problem of the SMA, this paper proposes a stagnant replacement mechanism (SRM). In the SRM, the stagnant individuals will be replaced by the individuals generated by Equation (15).

(15) $$P_{i}^{t+1}=\left\{\begin{array}{l} P_{i}^{t}+{\left(1-(t/T)\right)}^{2\times \left(1-(t/T)\right)}\times U\times \left(lb+rand\times \left(ub-Lb\right)\right),rand\leq 0.2\\ P_{i}^{t}+\left(0.2\times \left(1-rand\right)+rand\right)\left(P_{a}^{t}-P_{b}^{t}\right),rand>0.2 \end{array}\right.$$

In the original SMA, there is a lack of information exchange mechanism between the mucilage individuals. This is one of the reasons for the defective convergence speed and accuracy of the SMA. The SRM can effectively promote the information exchange within the population and the interaction between individuals. Therefore, it accelerates the movement of searching individuals to the optimal solution or near-optimal solution. In addition, it promotes the exploration of different regions within the search space and helps to escape from the local optimum. When each individual, the algorithm generates a new solution randomly and continues the search. This strategy increases the probability of finding the most efficient solution to the problem and improves the robustness of the algorithm. We set a counter $Rm_{i}$ for each individual with an initial value of 0. After each iteration, we determine whether each individual has gotten better. If it does not get better, the counter $Rm_{i}$ is added 1. If it gets better, it is not added. The SRM executes when $Rm_{i}>\log\left(t\right)$.

### 3.4. The Implementation Steps of the BWSMA

**Figure 2:**
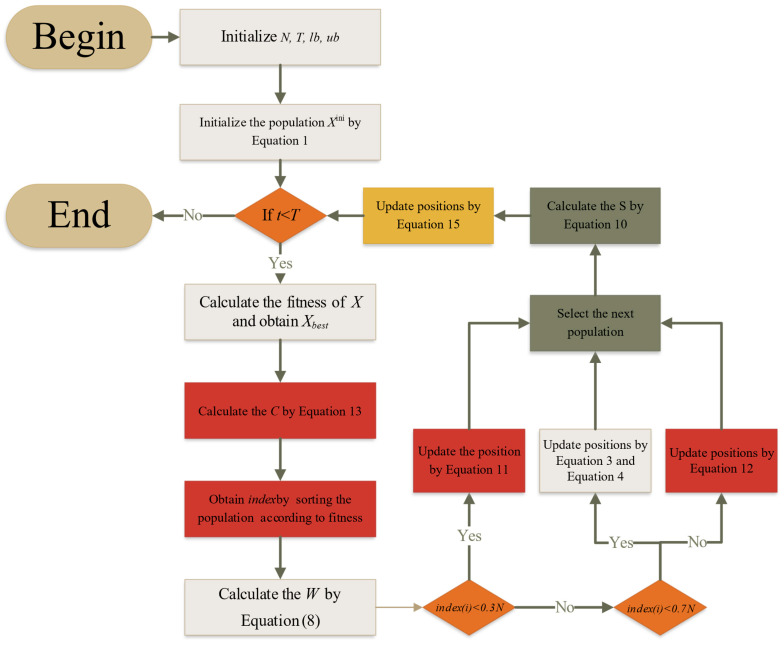
The flowchart of the BWSMA.

The proposed BWSMA is obtained by combining the basic SMA with the adaptive greedy mechanism, best–worst management strategy, and stagnant replacement mechanism whose pseudo-code and flowchart are shown in Algorithm 1 and Figure 2. The initialization phase of the BWSMA is the same as the basic SMA. At each iteration, the population is divided into three subpopulations based on fitness ordering. The superior and inferior populations employ the best–worst management strategy. The balanced populations use Equations (3) and (4) of the basic SMA. After updating, all populations use the adaptive greedy mechanism to select individuals from the offspring and parent individuals to participate in the next iteration. Then, it is determined whether it is in stagnation or not, and the stagnant replacement mechanism is applied.
**Algorithm 1** Pseudo-code of the BWSMA**Begin**: //Initialization   Initialize z, *N*, *T*, *lb*, *ub*;  Initialize the population $X^{ini}$ by Equation (1) //Main loop  **While** (*t* < *T*) **do**   Calculate the fitness of X and obtain
$X_{best}$
   Calculate the *C* by Equation (13)//**Best–worst management strategy**   Obtain *index* by sorting the population X according to fitness   **For** *i* = 1: *N* **do**    **If** *index*(*i*) < 0.3*N* **then**     Update the position by Equation (11)//**Best–worst management strategy**    **Else if** *index*(*i*) < 0.7*N*
**then**     Update positions by Equations (3) and (4)    **Else**
     Update the position by Equation (12)//**Best–worst management strategy**    **End if**
    **End if**
   **End For**
   Select the next population//**Adaptive greedy mechanism**   Calculate the S by Equation (10)//**Adaptive greedy mechanism**   **For** *i* = 1: *N* **do**    Update positions by Equation (15)//**Stagnant replacement mechanism**   **End for**
   *t* = *t* + 1  **End While**
**Return**: the best fitness and
$X_{best}$

Time complexity is a measure of the performance of an algorithm. This work proposes the BWSMA by combining the AGS, BWM, and SRM. According to Algorithm 1 and Figure 2, the BWSMA consists of population initialization, the AGS phase, the original search strategy, and the SRM phase. Here, we assume that the problem dimension is D, the number of populations is N, and the maximum number of iterations is T. Then, the complexity of the initialization step is $O\left(N\times D\right)$. At one iteration, each individual will only select one of the AGS and the original search strategy, and thus, the complexity of the position updating is $O\left(N\times D\times T\right)$. In addition, since the SRM strategy is executed when an individual falls into a stagnation, we assume that the individuals executing the SRM at each iteration consist of N1 individuals; then, the complexity of this strategy is $O\left(N_{1}\times D\times T\right)$. Therefore, the total time complexity of the proposed BWSMA is denoted as $O\left(D\times T\times \left(N_{1}+N\right)\right)$.

## 4. Experimental Results and Discussion

The test sets CEC2018 and CEC2022 from the IEEE Congress on Evolutionary Computation are chosen as test functions. Eight SMA variants and eight other advanced improved algorithms are selected for comparison. All experiments in this paper were conducted on a platform with the following specifications: a Windows 11 operating system, MATLAB R2021b, an AMD R9 7945HX CPU, and 32 GB RAM.

### 4.1. Test Functions and Evaluation Metrics

The IEEE CEC2018 test suite and the IEEE CEC2022 test suite are two popular public test suites that are widely used. The CEC2018 test suite contains 29 different classes of functions. The CEC2022 test suite contains 12 functions with different characteristics. These functions include unimodal, multimodal, hybrid, and composite functions that check the local search, global exploration, and ability of an algorithm to jump out of a local optimum. More information about the CEC2018 test suite and the CEC2022 test suite can be found in Table A1 and Table A2 in Appendix A. In the experiments of this paper, the dimensions of the CEC2018 test suite are set to 10, 30, 50, and 100. The dimensions of the CEC2022 test suite are set to 10 and 20. The population size of each algorithm is set to 50, and the maximum number of iterations is set to 300. Since metaheuristic algorithms belong to the category of stochastic algorithms, in order to circumvent the evaluation discrepancy caused by the randomness, all the experiments of this paper are required to be conducted 30 times. In addition, the Friedman test, Wilcoxon rank-sum test, and Nemenyi post hoc test are used to analyze the experimental data comprehensively. The Friedman test is used for overall comparison, which can examine the overall differences between the compared algorithms. The Wilcoxon rank-sum test is used for two-by-two comparisons and can examine whether there is a difference between two algorithms or not. The Nemenyi post hoc test is based on the Friedman test and can quantify the magnitude of the difference between different algorithms. It should be noted that all statistical tests were performed at a significance level of 0.05.

### 4.2. Comparison with BWSMA-Derived Algorithms

This paper proposes three improvement techniques to enhance the performance of the basic SMA. In order to analyze the impact of different improvement strategies on the BWSMA, the ablation experiments are performed in this section. Based on the three improvement strategies, we propose SMA-AGM, SMA-BWS, and SMA-SRM, which combine the adaptive greedy mechanism, best–worst management strategy, and stagnant replacement mechanism, respectively. By comparing the results of SMA-AGM, SMA-BWS, SMA-SRM, the SMA, and the BWSMA on the CEC2018 and CEC2022 test suites, we can demonstrate that these three mechanisms are effective in improving the performance of the mbasic SMA.

Since there are six cases in the two test sets and a full presentation of these results would take up more space, these results are summarized in Table A3, Table A4, Table A5, Table A6, Table A7 and Table A8 in Appendix A, which include the best value (Best), average value (Mean), standard deviation (Std), and the rank based on the mean value (Rank). In this subsection, the obtained results will be analyzed through statistical tests.

**Figure 3:**
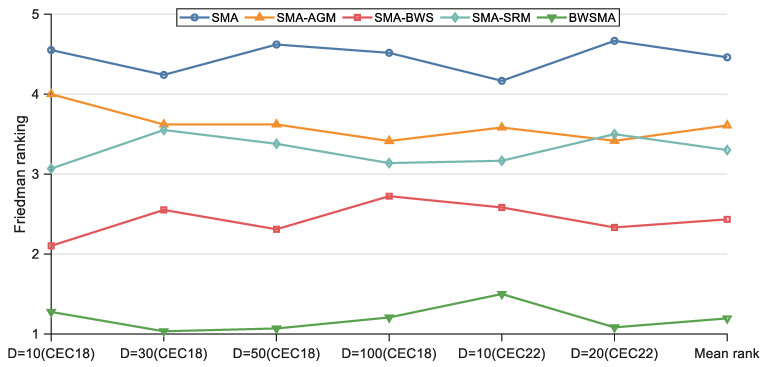
Friedman rankings of BWSMA and its derived algorithms (a = 0.05).

The results of the Friedman test based on the results of ablation experiments are shown in Table 1, where “Mean rank” indicates the average ranking and “Overall rank” indicates the ordering, and the rankings of each algorithm in different dimensions are visualized in Figure 3. Based on the Friedman *p*-values in Table 1, it is clear that there is a significant difference between the BWSMA, SMA-AGM, SMA-BWS, SMA-SRM, and basic SMA, which are significantly different from each other. Specifically, the BWSMA ranked first on all six cases, achieving an average ranking of 1.195. The three SMA variants also ranked above the basic SMA, with rankings of 3.609, 2.434, and 3.301, respectively. These results provide a preliminary indication of the effectiveness and compatibility of the three improved strategies.

Table 2 provides the corresponding Wilcoxon rank-sum test results, where the “+” sign indicates that the BWSMA and derived algorithms outperform the basic SMA, “−” indicates that the BWSMA and derived algorithms are inferior to the basic SMA, and “=” indicates that the BWSMA and derived algorithms perform similarly to the basic SMA. According to Table 2, we can find that both the BWSMA and the three SMA variants combining a single mechanism obtain more “+” than “−”, which indicates that each mechanism improves the performance of the basic SMA. Specifically, although the AGM mechanism improves the least, the SMA-AGM applying AGM is inferior to the basic SMA in only at most one function. On the contrary, the SRM, although it improves a lot, also weakens the SMA in many functions. The BWS contributes the most to the BWSMA, which is evident from the results in both Table 1 and Table 2. From this we can conclude that BWS contributes the most, followed by SRM and AGM. The BWSMA is most powerful when all three improvement mechanisms are combined.

**Figure 4:**
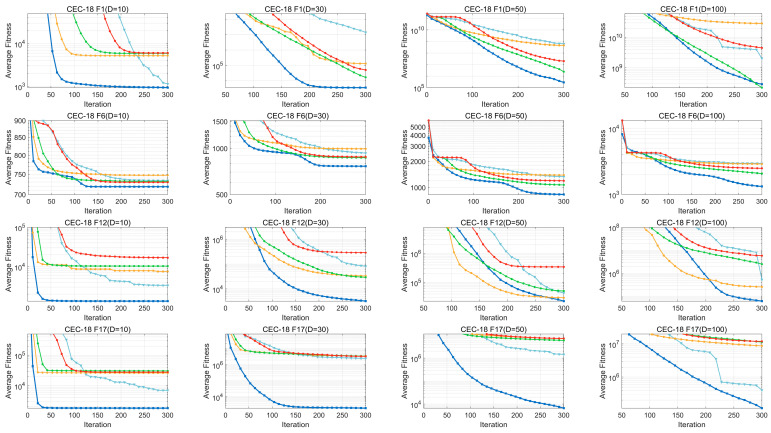

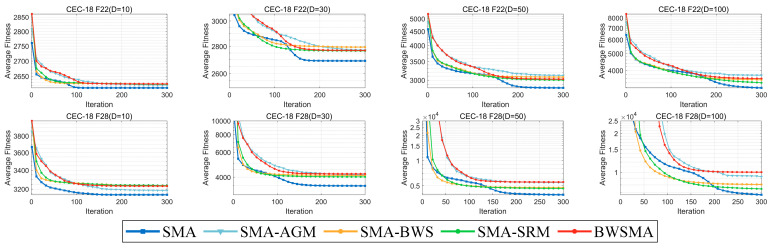
Convergence curve of BWSMA and its derived algorithms on CEC2018 test suite.

**Figure A1:**
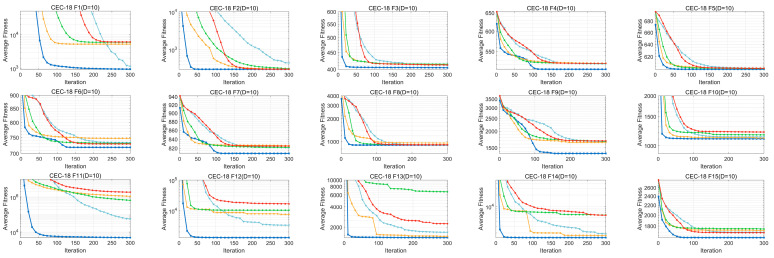

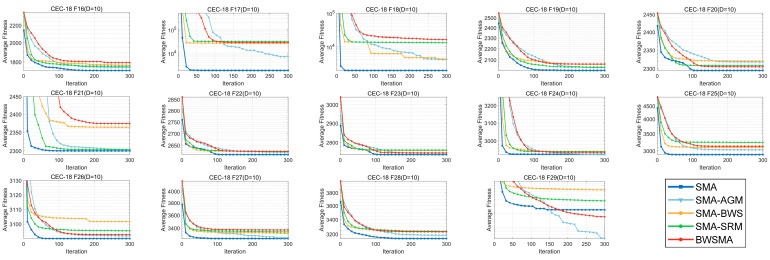
Convergence curve of BWSMA and its derived algorithms on CEC2018 (10D).

**Figure A2:**
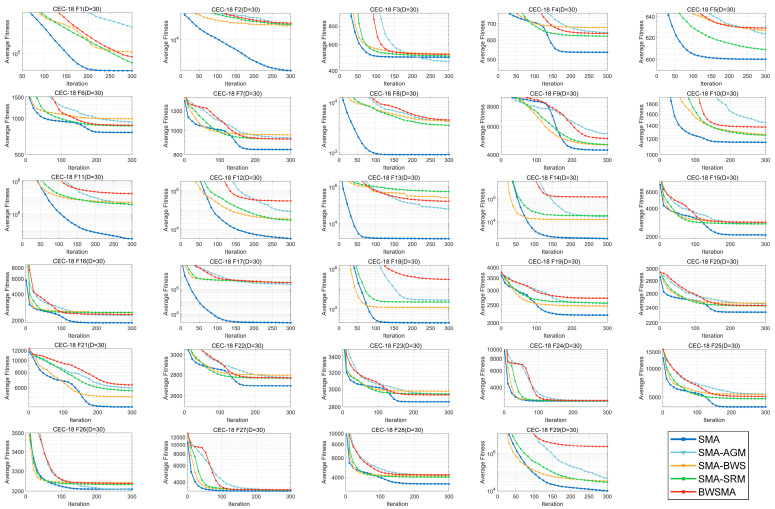
Convergence curve of BWSMA and its derived algorithms on CEC2018 (30D).

**Figure A3:**
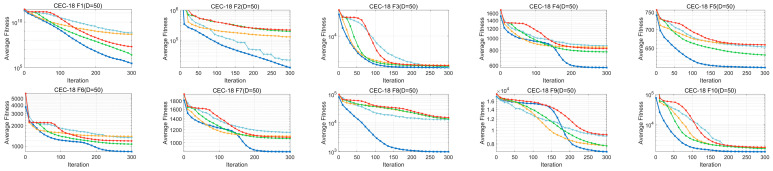

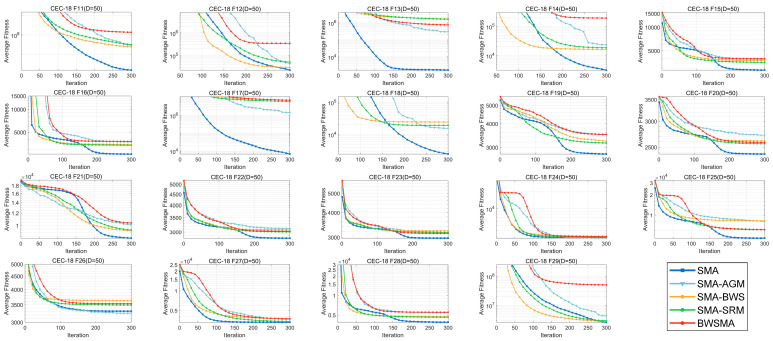
Convergence curve of BWSMA and its derived algorithms on CEC2018 (50D).

**Figure A4:**
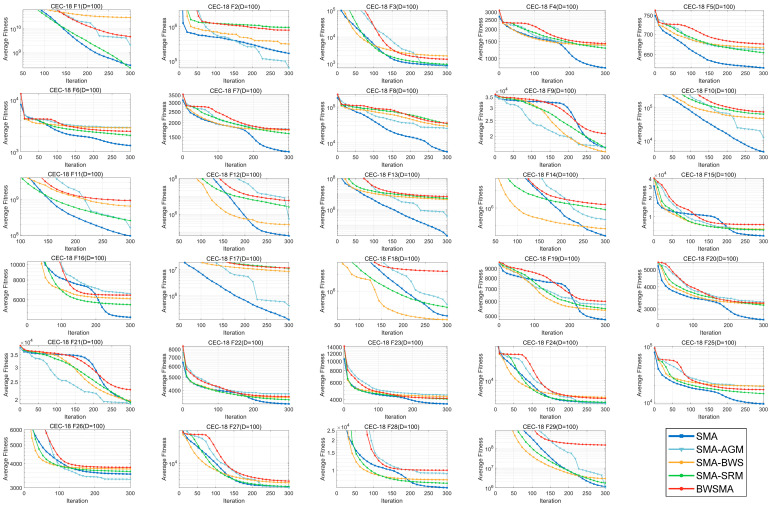
Convergence curve of BWSMA and its derived algorithms on CEC2018 (100D).

Convergence curves for the BWSMA, SMA, and BWSMA-derived algorithms on solving the CEC2018 test suite are provided in Figure 4, where the blue line represents the BWSMA. The complete convergence curves are summarized in Figure A1, Figure A2, Figure A3 and Figure A4 in Appendix A, considering the coherence of the reading. In the unimodal function F1, only the BWSMA and SMA-AGM are able to converge quickly, while the other two variants and the basic SMA stagnate very early and are unable to search further. This indicates that the AGM is able to help the algorithm converge quickly through the greedy mechanism. The convergence accuracy of the BWS and SRM is still higher than that of the basic SMA even though they are unable to search further, due to the fact that both the BWS and SRM are able to expand the search range and jump out of the local optimum. The opposite is true for the multi-peak function F6. The selection mechanism of the AGM makes the SMA easy to fall into the local optimum, while the SRM helps stagnant individuals to restart the search. As can be seen from the curve of the BWSMA, it stagnates in the middle of the process but quickly escapes from the local optimum. This is attributed to the stagnation-lifting effect of the SRM, which enhances the algorithm’s ability to avoid local stagnation by updating the stagnant individuals. For the hybrid functions F12 and F17, the BWSMA is able to converge stably and quickly to a better value, which is attributed to the fact that the AGM helps the BWSMA to converge quickly, and the BWS, with its bootstrapping effect, helps the BWSMA to find promising regions, which improves the algorithm’s convergence speed and accuracy. For the combined functions F22 and F28, the BWSMA stagnates in the middle but quickly continues the search and provides a high convergence accuracy. This is due to the fact that the SRM can reset the stagnant individuals, and the BWS and AGM allow the dominant individuals to accelerate convergence. In summary, the AGM accelerates the convergence of the BWSMA, the BWS enhances the performance of the BWSMA in general, and the SRM helps the BWSMA jump out of the local optimum. In other words, the three improvement strategies proposed in this paper improve the optimization ability of the BWSMA in an all-around way.

### 4.3. Comparison with SMA Variant Algorithms

**Figure 5:**
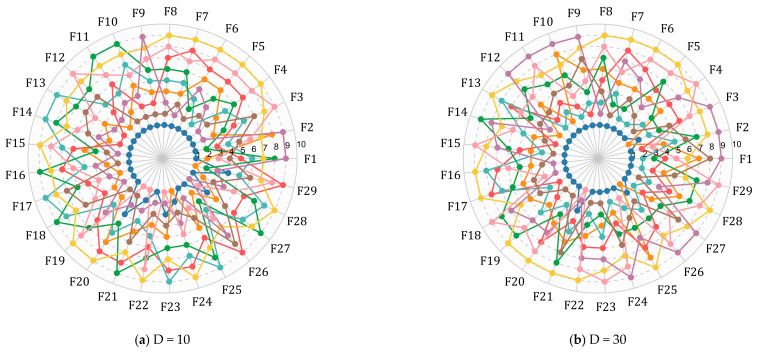

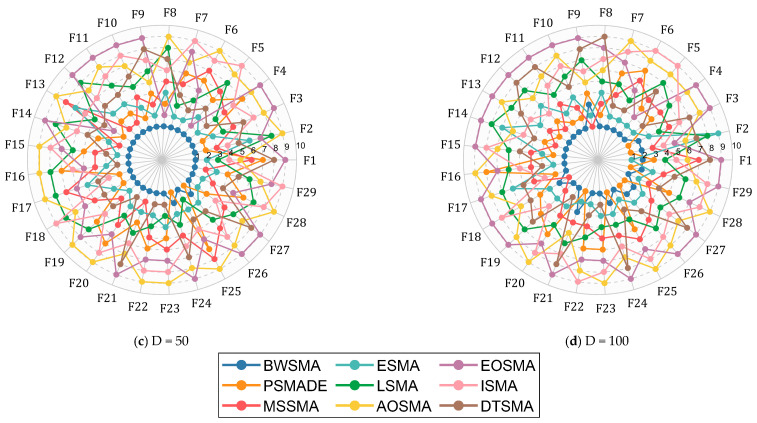
Algorithm performance-sorting radar chart of BWSMA and SMA variants.

In this subsection, we compare the BWSMA with eight SMA variants to illustrate the superiority of the proposed algorithm in this paper. These variants include PSMADE, MSSMA, ESMA, LSMA, AOSMA, EOSMA, ISMA, and DTSMA. Table 3 shows the specific parameters of these selected comparison algorithms. The best values, standard deviations, average values, and rankings obtained by the BWSMA and the eight SMA variants on the CEC2018 test suite are summarized in Table A9, Table A10, Table A11 and Table A12 in Appendix A. The rankings of the BWSMA and SMA variants on each function under the four dimensions are directly depicted in Figure 5. Within Figure 5, the rankings of each algorithm on the different functions enclose a surface, and the smaller the surface area, the better the performance of the algorithm. According to Figure 5, BWSMA has the smallest surface, which means that the overall performance of BWSMA is better than other SMA variants.

**Figure 6:**
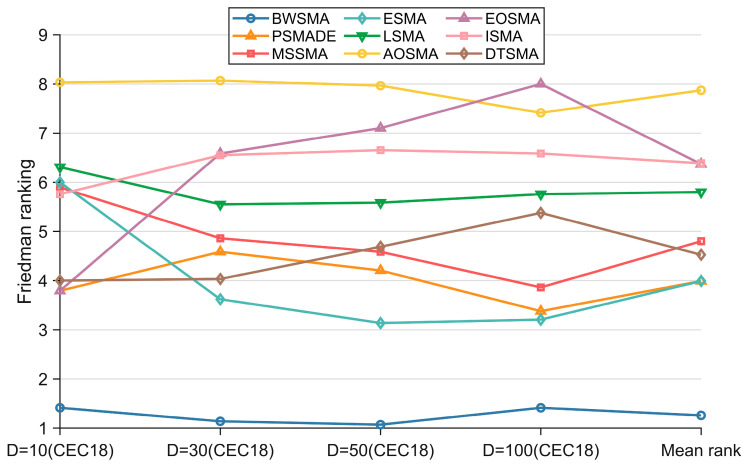
Friedman rankings of BWSMA and SMA variants (a = 0.05).

**Figure 7:**
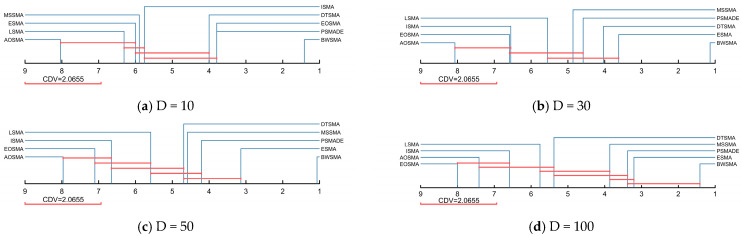
Nemenyi test results of BWSMA and SMA variants (a = 0.05).

Table 4 summarizes the results of the Wilcoxon rank-sum test between the BWSMA and SMA variants, where the symbols “+/=/−” indicate that the BWSMA is superior/similar/inferior to other SMA variants on the CEC2018 test set. According to Table 4, the BWSMA has a clear advantage, with significantly more “+” than “−” and even more than the total number of “−” and “=”. This indicates that the BWSMA has a clear dominance over other SMA variants on the CEC2018 test set. Table 5 presents the Friedman test results for the BWSMA and SMA variants, whose rankings are visualized in Figure 6. Based on the results of Friedman’s test for BWSMA and SMA variants, the BWSMA ranked first among all the algorithms, achieving an average ranking of 1.259. PSMADE and ESMA tied for second place. Overall, there is a significant difference between the BWSMA and the other SMA variants, which is derived from a *p*-value of less than 0.05. To further quantify the magnitude of the difference between the BWSMA and SMA variants, Nemenyi post hoc tests were performed. In the Nemenyi post hoc test, the critical difference value (CDV) can be calculated based on the number of functions and the number of algorithms [6]. The Nemenyi test assumes that two algorithms with similar performance will be connected by line segments of length CDV and that algorithms with significant gaps will not be connected by line segments between them. Figure 7 visualizes the size of the difference between the BWSMA and SMA variants. As shown in Figure 7, there are significant differences between the BWSMA and MSSMA, ESMA, LSMA, AOSMA, EOSMA, ISMA, and DTSMA on the 10D, 30D, and 50D CEC2018 test sets. On 100D, there is no significant difference between the performance of the BWSMA and PSMADE or ESMA, but it is significantly better than the MSSMA, LSMA, AOSMA, EOSMA, ISMA, and DTSMA.

In conclusion, the proposed BWSMA is a high-performing SMA variant with strong optimization effects in comparison with other SMA variants.

### 4.4. Comparison with Other Advanced Improved Algorithms

For this subsection, eight other advanced improved algorithms are selected as comparison algorithms and are fully compared with the BWSMA on the CEC2022 test suite. These algorithms include the NMPA, BEESO, FDBARO, WOSSA, PHISAO, ESLPSO, EPSCA, and IGWO, whose corresponding parameter settings are displayed in Table 6. The NMPA is a variant of the marine predator algorithm that introduces nonlinear control parameters. BEESO is an improved snake optimizer that combines elite backward learning and two-way search. FDBARO is a variant of the artificial rabbit optimizer that incorporates a fitness distance balancing strategy. WOSSA is an improved sparrow search algorithm that brings together an adaptive weight jumping mechanism and a suicide variant perturbation. PHISAO enhances the performance of the snow ablation optimizer by introducing multiple swarm iteration strategies and Cauchy mutation perturbations. ESLPSO introduces nonlinear control coefficients and interactive learning mechanisms to enhance the performance of social learning Particle Swarm Optimization. EPSCA is a modified sine–cosine algorithm that combines an elite pooling strategy and a pattern search approach. IGWO introduces a dimensional learning strategy to enhance the global search capability of the Gray Wolf Optimizer.

**Figure 8:**
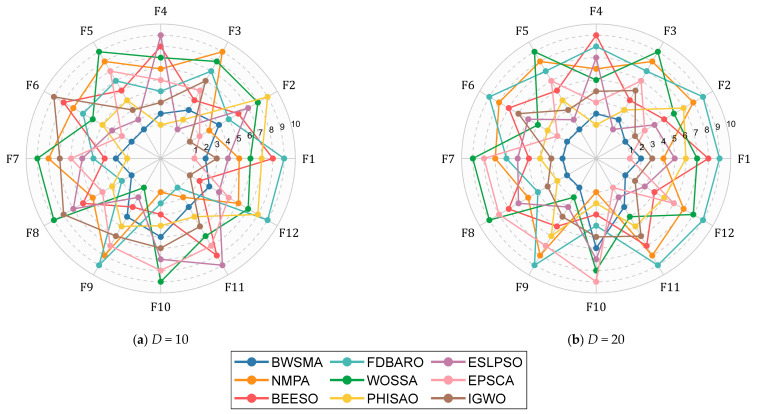
Algorithm performance-sorting radar chart of BWSMA and other improved algorithms.

Table A13 and Table A14 in Appendix A show the average values, best values, standard deviations, and ranks of the BWSMA and the other improved algorithms on the CEC2022 test set. Similarly, radar charts are utilized to visualize the rankings of the BWSMA and the comparison algorithms on the CEC2022 test set, as shown in Figure 8.

The BWSMA performs the best overall, as seen in Figure 8, where it attains the least surface area. The BWSMA is able to provide the minimum average value for three and seven functions on 10D and 20, respectively. In contrast, the other improved algorithms ranked first on at most one function. Table 7 lists the results of the statistical tests for BWSMA and the improved algorithms. According to the Friedman test results in Table 7, the *p*-value of both dimensions is less than 0.05, which means that there is a significant difference between the BWSMA and the improved algorithms on the CEC2022 test set. Specifically, the BWSMA received Friedman scores of 2.583 at 10D and 1.833 at 20D. The AOSMA, which came in second, had scores of 4.333 and 3.917. Overall, the BWSMA was the best overall performer, although it had an average performance on 10D, with only three functions in first place. In conjunction with the Wilcoxon rank-sum test in Table 7, the BWSMA receives more “+” than “−”, indicating that it can achieve significantly better results in most functions.

**Figure 9:**
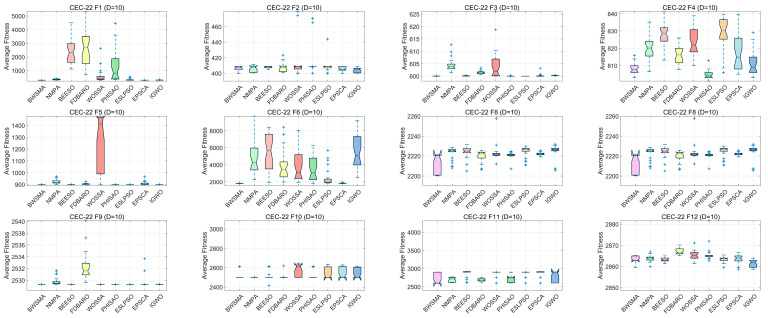
Boxplots of BWSMA and improved algorithm based on CEC2022 (10D).

**Figure 10:**
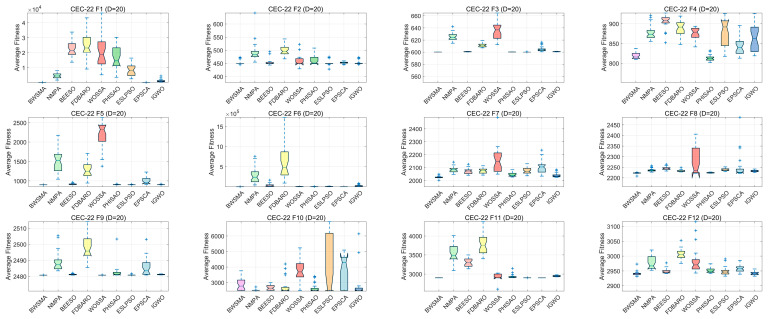
Boxplots of BWSMA and improved algorithm based on CEC2022 (20D).

Box plots can reflect the stability of the algorithms. Figure 9 and Figure 10 show box plots of the BWSMA and improved algorithms solving problems of different dimensions of the CEC2022 test set. Box plots are used to show the performance distribution of multiple algorithms over different test functions and multiple runs. Each box represents the statistical distribution of an algorithm, including the median value (center line), upper and lower quartiles (top and bottom edges of the box), maximum and minimum values (whiskers), and possible outliers (points). According to the results shown in Figure 9 and Figure 10, in most cases, the box plots of the BWSMA table contain a lower median and box, narrower box heights, and fewer outliers, which indicates that the BWSMA has higher stability and convergence accuracy compared to the other eight improved algorithms. Overall, the BWSMA provides an acceptable and favorable solution in terms of the balance between exploitation and utilization, indicating that it has the potential to address real-world applications. In summary, the BWSMA has excellent search capability on the CEC2022 test set, and even when compared with other state-of-the-art improved algorithms, it still has excellent performance, showing significant search accuracy and robustness.

## 5. Experiments Using Real-World Engineering Problems

In this section, we apply the BWSMA to engineering constrained-optimization problems to verify its potential for solving real-world optimization problems. To assess the utility and scalability of the algorithm, we apply it to two typical real-world engineering problems. The comparison algorithms include the basic ECO algorithm and the top three algorithms other than the BWSMA in both test sets.

### 5.1. Tension/Compression Spring Design Problem

**Figure 11:**
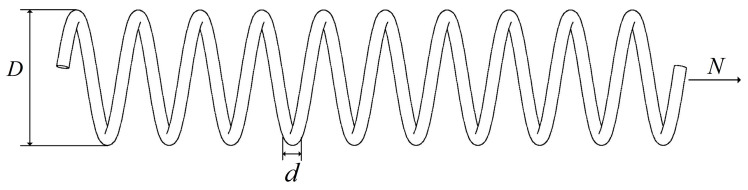
Tension/compression spring design structure.

This design problem aims to minimize the weight of a stretch/compression spring by finding three crucial parameters of the spring, including wire diameter (*d*), coil diameter (*D*), and the number of coils (*N*). The structure of this engineering problem is illustrated in Figure 11, and the mathematical model can be found in the literature [87]. The optimization results of the BWSMA and seven different algorithms on the tension/compression spring design structure are given in Table 8. From the table, it can be seen that the optimization results of the BWSMA are better than the other compared algorithms, and the optimal value obtained is 1.27E−02.

### 5.2. Pressure Vessel Design Problem

**Figure 12:**
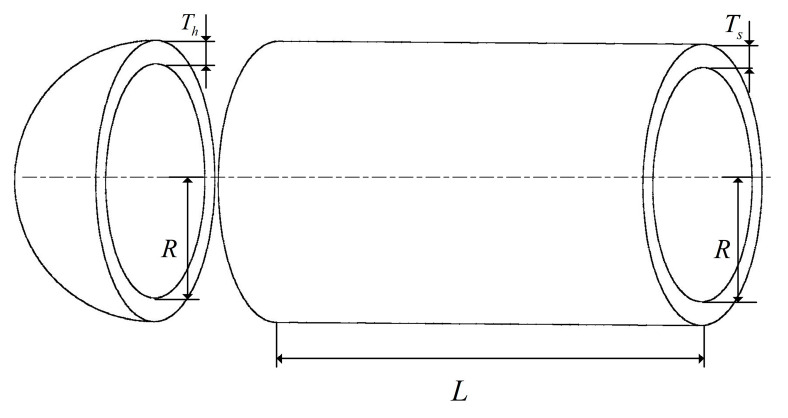
Pressure vessel design problem structure.

Figure 12 depicts the pressure vessel design problem. The objective is to minimize total cost while satisfying operational constraints, with decision variables comprising shell thickness (*Ts*), head thickness (*Th*), inner radius (*R*), and head length (L); the corresponding mathematical formulation is given in the literature [88]. From the results in Table 9, it is evident that the BWSMA’s optimal value surpasses all other competitors, achieving a minimum cost of 5.89E+03.

## 6. Conclusions

This paper is dedicated to addressing the shortcomings of the SMA and proposes an SMA variant, the BWSMA, by combining three improvement mechanisms. The AGM strategy improves the convergence speed and accuracy of the SMA. The BWS directs the SMA to search towards promising regions and improves its search capability. The SRM prevents the population from falling into a local optimum by updating stagnant individuals. To verify the performance of the BWSMA, this paper conducts comprehensive experiments on the CEC2018 test set, CEC2022 test set, and engineering constrained-optimization problems. Ablation experiments confirm that the three improvement strategies can enhance the performance of the SMA individually and further strengthen the SMA when combined. Comparison results with eight SMA variants indicate that the proposed BWSMA is an excellent SMA variant with strong optimization capabilities. Comparison results with eight state-of-the-art improved algorithms indicate that the BWSMA is a promising metaheuristic algorithm variant. In conclusion, the improvement strategies proposed in this paper distinctly enhance the performance of the basic SMA and alleviate its limitations. However, it cannot be denied that it still has shortcomings. The BWS strategy is extremely dependent on the quality of dominant populations and is difficult to accurately fit the evolutionary direction when the number of populations is small. The execution frequency of the introduced SRM mechanism needs to be further investigated, and indicators that better reflect the stagnation situation need to be studied. Finally, we only examined the performance of the BWSMA on test functions, and its scalability and adaptability need to be tested in more optimization scenarios.

We will consider the following aspects in our future work: First, we will examine the performance of the BWSMA on other optimization domains such as optimal power flow optimization, medical image segmentation, and UAV mission planning. Second, we will develop a multi-objective version of the BWSMA to solve complex multi-objective optimization problems in the real world such as model calibration problems [89]. Thirdly, we will try to modify the structure of the BWSMA to make it suitable for real-time optimization problems. Finally, We will apply the BWSMA to optimize the hyperparameters of various machine learning methods to address a wider range of applications. For example, it can be combined with the CNN for system monitoring [90], with the random forest approach for motion recognition [91], with the LSTM for energy system management [92], and with the feed-forward neural network for electronic detection [93].

## Figures and Tables

**Table 1:** Friedman test results obtained by BWSMA and its derived algorithms (a = 0.05).

Test Suite	Dimension	SMA	SMA-AGM	SMA-BWS	SMA-SRM	BWSMA	*p*-Value
CEC2018	10	4.552	4.000	2.103	3.069	1.276	3.33E−17
	30	4.241	3.621	2.552	3.552	1.034	5.22E−15
	50	4.621	3.621	2.310	3.379	1.069	1.26E−17
	100	4.517	3.414	2.724	3.138	1.207	9.33E−14
CEC2022	10	4.167	3.583	2.583	3.167	1.500	5.15E−04
	20	4.667	3.417	2.333	3.500	1.083	4.36E−07
Mean rank		4.461	3.609	2.434	3.301	1.195	N/A
Overall rank		5	4	2	3	1	N/A

**Table 2:** Wilcoxon rank-sum test results obtained by BWSMA and its derived algorithms (a = 0.05).

vs. SMA +/=/−	CEC-2018 Test Suite				CEC-2022 Test Suite	
	10D	30D	50D	100D	10D	20D
SMA-AGM	9/19/1	7/22/0	8/21/0	21/7/1	4/8/0	4/8/0
SMA-BWS	26/2/1	19/8/2	24/5/0	22/7/0	9/3/0	10/2/0
SMA-SRM	21/5/3	18/6/5	18/8/3	22/1/6	5/4/3	4/5/3
BWSMA	29/0/0	29/0/0	28/1/0	28/1/0	11/1/0	12/0/0

**Table 3:** Parameter settings of SMA variants.

Algorithm	Parameter Settings
BWSMA	$S^{ini}=0.5$
PSMADE [71]	$z=0.03,\beta_{\min}=0.4,\beta_{\max}=1,CR=0.5$
MSSMA [72]	z=0.03,E=100,Ns=10
ESMA [73]	z=0.03
LSMA [74]	z=0.03
AOSMA [75]	z=0.03
EOSMA [76]	z=0.6,q=0.2,GP=0.5
ISMA [77]	$z=0.03,S_{\max}=N,S_{\min}=0.5N$
DTSMA [78]	z=0.03,Cr=0.5,q=0.9

**Table 4:** Wilcoxon rank-sum test results obtained by BWSMA and SMA variants (a = 0.05).

BWSMA vs. +/=/−	Dimension	PSMADE	MSSMA	ESMA	LSMA	AOSMA	EOSMA	ISMA	DTSMA
CEC-2018 test suite	10D	24/5/0	29/0/0	29/0/0	28/0/1	29/0/0	24/4/1	25/2/2	28/1/0
	30D	24/3/2	28/1/0	28/1/0	29/0/0	29/0/0	28/1/0	27/2/0	29/0/0
	50D	25/2/2	27/2/0	29/0/0	28/1/0	29/0/0	29/0/0	29/0/0	29/0/0
	100D	19/4/6	26/2/1	23/6/0	29/0/0	29/0/0	29/0/0	29/0/0	29/0/0

**Table 5:** Friedman test results obtained by BWSMA and SMA variants (a = 0.05).

Algorithm	CEC-2018 Test Suite					
	10D	30D	50D	100D	Mean Rank	Overall Rank
BWSMA	1.414	1.138	1.069	1.414	1.259	1
PSMADE	3.793	4.586	4.207	3.379	3.991	2
MSSMA	5.897	4.862	4.586	3.862	4.802	5
ESMA	6.000	3.621	3.138	3.207	3.991	2
LSMA	6.310	5.552	5.586	5.759	5.802	6
AOSMA	8.034	8.069	7.966	7.414	7.871	9
EOSMA	3.793	6.586	7.103	8.000	6.371	7
ISMA	5.759	6.552	6.655	6.586	6.388	8
DTSMA	4.000	4.034	4.690	5.379	4.526	4
*p*-value	1.92E−21	1.90E−23	2.79E−26	7.65E−28	N/A	N/A

**Table 6:** Parameter settings of improved algorithms.

Algorithm	Parameter Settings
NMPA [79]	FADs=0.2,P=0.5
BEESO [80]	$a=0.25,b=0.6,c_{1}=0.5,c_{2}=0.05,c_{3}=2$
FDBARO [81]	w=0.5
WOSSA [82]	p=0.2
PHISAO [83]	a=0.2,b=0.8
ESLPSO [84]	CR=0.5,F=0.5,c=0.1,M=N
EPSCA [85]	a=2,β=0.5,c=0.5,d=1.2
IGWO [86]	N/A

**Table 7:** Statistical test results of BWSMA and other improved algorithms.

Algorithm	CEC-2022 Test Suite					
	Friedman Test				Wilcoxon Rank-Sum Test	
	10D	20D	Mean Rank	Overall Rank	10D	20D
BWSMA	2.583	1.833	2.208	1	BWSMA vs. +/=/−	
BWSMA	5.667	6.500	6.083	7	9/2/1	11/1/0
NMPA	5.333	5.667	5.500	6	8/2/2	11/1/0
BEESO	5.333	7.500	6.417	8	9/2/1	11/1/0
FDBARO	7.000	6.667	6.833	9	9/2/1	10/2/0
WOSSA	4.333	3.917	4.125	2	5/4/3	9/2/1
PHISAO	5.000	4.167	4.583	4	7/3/2	9/1/2
ESLPSO	4.917	5.000	4.958	5	7/4/1	9/1/2
EPSCA	4.833	3.750	4.292	3	9/1/2	10/2/0
*p*-value	2.46E−02	2.83E−06	N/A	N/A	N/A	N/A

**Table 8:** Experimental results of tension/compression spring design structure.

Algorithm	Optimal Values for Variable			Optimal Value	Ranking
	*d*	*D*	*N*		
BWSMA	5.171E−02	3.559E−01	1.132E+01	1.268E−02	1
SMA	5.301E−02	3.842E−01	1.001E+01	1.298E−02	8
ESMA	5.171E−02	3.573E−01	1.128E+01	1.272E−02	4
PSMADE	5.163E−02	3.549E−01	1.141E+01	1.269E−02	3
DTSMA	5.191E−02	3.630E−01	1.101E+01	1.270E−02	5
PHISAO	5.142E−02	3.500E−01	1.171E+01	1.273E−02	6
EPSCA	5.231E−02	3.722E−01	1.050E+01	1.271E−02	7
WOSSA	5.159E−02	3.550E−01	1.141E+01	1.271E−02	2

**Table 9:** Experimental results of pressure vessel design problem structure.

Algorithm	Optimal Values for Variable				Optimal Value	Ranking
	*T_s_*	*T_h_*	*R*	*L*		
BWSMA	7.781E−01	3.850E−01	4.030E+01	2.001E+02	5.890E+03	1
SMA	7.929E−01	4.101E−01	4.100E+01	1.911E+02	6.879E+03	8
ESMA	7.780E−01	3.850E−01	4.030E+01	2.000E+02	5.971E+03	3
PSMADE	7.931E−01	3.920E−01	4.101E+01	1.901E+02	6.290E+03	5
DTSMA	7.780E−01	3.851E−01	4.030E+01	2.000E+02	5.892E+03	2
PHISAO	7.789E−01	3.851E−01	4.039E+01	2.000E+02	6.081E+03	4
EPSCA	8.830E−01	4.401E−01	4.571E+01	1.371E+02	6.310E+03	6
WOSSA	7.801E−01	3.850E−01	4.038E+01	1.991E+02	6.442E+03	7

## Data Availability

The data are provided within the manuscript.

## Appendix A

**Table A1:** CEC-2018 test functions.

Type	ID	Function Names	fmin
Unimodal functions	F1	Shifted and Rotated Bent Cigar Function	100
	F2	Shifted and Rotated Zakharov Function	300
Multimodal functions	F3	Shifted and Rotated Rosenbrock Function	400
	F4	Shifted and Rotated Rastrigin Function	500
	F5	Shifted and Rotated Expanded Scaffer Function	600
	F6	Shifted and Rotated Lunacek Bi_Rastrigin Function	700
	F7	Shifted and Rotated Non-Continuous Rastrigin Function	800
	F8	Shifted and Rotated Levy Function	900
	F9	Shifted and Rotated Schwefel Function	1000
Hybrid functions	F10	Hybrid Function 1 (N = 3)	1100
	F11	Hybrid Function 2 (N = 3)	1200
	F12	Hybrid Function 3 (N = 3)	1300
	F13	Hybrid Function 4 (N = 4)	1400
	F14	Hybrid Function 5 (N = 4)	1500
	F15	Hybrid Function 6 (N = 4)	1600
	F16	Hybrid Function 6 (N = 5)	1700
	F17	Hybrid Function 6 (N = 5)	1800
	F18	Hybrid Function 6 (N = 5)	1900
	F19	Hybrid Function 6 (N = 6)	2000
Composition functions	F20	Composition Function 1 (N = 3)	2100
	F21	Composition Function 2 (N = 3)	2200
	F22	Composition Function 3 (N = 4)	2300
	F23	Composition Function 4 (N = 4)	2400
	F24	Composition Function 5 (N = 5)	2500
	F25	Composition Function 6 (N = 5)	2600
	F26	Composition Function 7 (N = 6)	2700
	F27	Composition Function 8 (N = 6)	2800
	F28	Composition Function 9 (N = 3)	2900
	F29	Composition Function 10 (N = 3)	3000
Search range: ${\left[-100,100\right]}^{D}$; dimension: 10/30/50/100			

**Table A2:** CEC-2022 test functions.

Type	ID	Function Names	fmin
Unimodal functions	F1	Shifted and Fully Rotated Zakharov Function	300
Basic functions	F2	Shifted and Fully Rotated Rosenbrock Function	400
	F3	Shifted and Fully Rotated Expanded Schaffer f6 Function	600
	F4	Shifted and Fully Rotated Non-Continuous Rastrigin Function	800
	F5	Shifted and Fully Rotated Levy Function	900
Hybrid functions	F6	Hybrid Function 1 (N = 3)	1800
	F7	Hybrid Function 2 (N = 6)	2000
	F8	Hybrid Function 3 (N = 5)	2200
Composition functions	F9	Composition Function 1 (N = 5)	2300
	F10	Composition Function 2 (N = 4)	2400
	F11	Composition Function 3 (N = 5)	2600
	F12	Composition Function 4 (N = 6)	2700
Search range: ${\left[-100,100\right]}^{D}$; dimension: 10/20			

**Table A3:** Results of ablation experiments with BWSMA based on CEC2018 (10D).

No.	Index	SMA	SMA-AGM	SMA-BWS	SMA-SRM	BWSMA
F1	Best	2.9078E+02	2.3390E+02	1.0245E+02	1.2021E+02	1.1525E+02
	Mean	7.4836E+03	6.3910E+03	1.8703E+03	6.5469E+03	9.2922E+02
	Std	3.9141E+03	4.2793E+03	2.2628E+03	4.1223E+03	7.5662E+02
	Rank	5	3	2	4	1
F2	Best	3.0001E+02	3.0000E+02	3.0000E+02	3.0011E+02	3.0000E+02
	Mean	3.0032E+02	3.0014E+02	3.0000E+02	3.0829E+02	3.0000E+02
	Std	1.0550E+00	4.8729E−01	1.5517E−03	2.2793E+01	1.6833E−03
	Rank	4	3	1	5	2
F3	Best	4.0003E+02	4.0333E+02	4.0183E+02	4.0161E+02	4.0080E+02
	Mean	4.2346E+02	4.1423E+02	4.0390E+02	4.0590E+02	4.0420E+02
	Std	3.2502E+01	2.3375E+01	9.1994E−01	1.3105E+00	1.1763E+00
	Rank	5	4	1	3	2
F4	Best	5.0796E+02	5.0798E+02	5.0199E+02	5.0601E+02	5.0299E+02
	Mean	5.1866E+02	5.1946E+02	5.0891E+02	5.1451E+02	5.0914E+02
	Std	6.7608E+00	6.7990E+00	3.7954E+00	4.7381E+00	3.7821E+00
	Rank	4	5	1	3	2
F5	Best	6.0007E+02	6.0006E+02	6.0002E+02	6.0031E+02	6.0000E+02
	Mean	6.0020E+02	6.0013E+02	6.0007E+02	6.0053E+02	6.0002E+02
	Std	9.8348E−02	5.2644E−02	3.7069E−02	1.1395E−01	2.4785E−02
	Rank	4	3	2	5	1
F6	Best	7.1335E+02	7.1531E+02	7.1290E+02	7.1495E+02	7.1320E+02
	Mean	7.3131E+02	7.2762E+02	7.2190E+02	7.2575E+02	7.1869E+02
	Std	7.0581E+00	8.4738E+00	5.4214E+00	6.9353E+00	3.2076E+00
	Rank	5	4	2	3	1
F7	Best	8.0797E+02	8.1095E+02	8.0398E+02	8.0408E+02	8.0199E+02
	Mean	8.2081E+02	8.1907E+02	8.0978E+02	8.1294E+02	8.0876E+02
	Std	9.4009E+00	6.6306E+00	4.8539E+00	4.4410E+00	3.7990E+00
	Rank	5	4	2	3	1
F8	Best	9.0000E+02	9.0000E+02	9.0000E+02	9.0007E+02	9.0000E+02
	Mean	9.0158E+02	9.0002E+02	9.0000E+02	9.0058E+02	9.0000E+02
	Std	8.1711E+00	8.3933E−02	3.3970E−04	8.2944E−01	1.6351E−02
	Rank	5	3	1	4	2
F9	Best	1.1536E+03	1.1519E+03	1.0184E+03	1.2391E+03	1.0103E+03
	Mean	1.7081E+03	1.6321E+03	1.5912E+03	1.5147E+03	1.3616E+03
	Std	2.6147E+02	2.4561E+02	2.9097E+02	1.6400E+02	2.1647E+02
	Rank	5	4	3	2	1
F10	Best	1.1082E+03	1.1050E+03	1.1012E+03	1.1014E+03	1.1003E+03
	Mean	1.1856E+03	1.1527E+03	1.1058E+03	1.1162E+03	1.1033E+03
	Std	9.7782E+01	8.1399E+01	3.3022E+00	3.5314E+01	1.4183E+00
	Rank	5	4	2	3	1
F11	Best	4.2818E+03	6.1369E+03	1.4034E+03	4.1131E+03	1.3188E+03
	Mean	4.2123E+05	1.3325E+05	2.7181E+03	3.1455E+04	4.7612E+03
	Std	4.8182E+05	1.7798E+05	2.5264E+03	2.8147E+04	8.4062E+03
	Rank	5	4	1	3	2
F12	Best	1.5841E+03	1.4169E+03	1.3076E+03	1.3290E+03	1.3032E+03
	Mean	1.4465E+04	1.6476E+04	1.3615E+03	2.6220E+03	1.3120E+03
	Std	1.3384E+04	1.3989E+04	5.7105E+01	5.6582E+03	5.8979E+00
	Rank	4	5	2	3	1
F13	Best	1.4418E+03	1.4257E+03	1.4011E+03	1.4021E+03	1.4000E+03
	Mean	2.8325E+03	2.1846E+03	1.4207E+03	1.4262E+03	1.4056E+03
	Std	3.3727E+03	1.5898E+03	1.0147E+01	1.1132E+01	8.0927E+00
	Rank	5	4	2	3	1
F14	Best	1.6070E+03	1.5256E+03	1.5004E+03	1.5047E+03	1.5002E+03
	Mean	8.8600E+03	5.2274E+03	1.5105E+03	1.5178E+03	1.5047E+03
	Std	8.0006E+03	3.5730E+03	8.9122E+00	1.4698E+01	4.8891E+00
	Rank	5	4	2	3	1
F15	Best	1.6019E+03	1.6011E+03	1.6007E+03	1.6006E+03	1.6005E+03
	Mean	1.6925E+03	1.6652E+03	1.6496E+03	1.6682E+03	1.6129E+03
	Std	9.0755E+01	7.8251E+01	6.2346E+01	7.9888E+01	2.4387E+01
	Rank	5	3	2	4	1
F16	Best	1.7041E+03	1.7058E+03	1.7013E+03	1.7044E+03	1.7006E+03
	Mean	1.7650E+03	1.7532E+03	1.7325E+03	1.7385E+03	1.7175E+03
	Std	4.5648E+01	3.7474E+01	1.8951E+01	3.0971E+01	1.2925E+01
	Rank	5	4	2	3	1
F17	Best	2.0563E+03	2.6257E+03	1.8052E+03	1.8894E+03	1.8004E+03
	Mean	2.7812E+04	2.6028E+04	1.8495E+03	3.0227E+03	1.8089E+03
	Std	1.6829E+04	1.6538E+04	3.2728E+01	3.3024E+03	9.1926E+00
	Rank	5	4	2	3	1
F18	Best	1.9841E+03	1.9047E+03	1.9016E+03	1.9046E+03	1.9001E+03
	Mean	9.8627E+03	1.3235E+04	1.9042E+03	1.9302E+03	1.9014E+03
	Std	1.1726E+04	1.1654E+04	1.9151E+00	8.1168E+01	1.4326E+00
	Rank	4	5	2	3	1
F19	Best	2.0051E+03	2.0013E+03	2.0052E+03	2.0020E+03	2.0000E+03
	Mean	2.0412E+03	2.0290E+03	2.0308E+03	2.0216E+03	2.0092E+03
	Std	3.8578E+01	2.7652E+01	1.7035E+01	1.2174E+01	1.0646E+01
	Rank	5	3	4	2	1
F20	Best	2.2036E+03	2.2035E+03	2.2000E+03	2.2004E+03	2.2000E+03
	Mean	2.3243E+03	2.3130E+03	2.3025E+03	2.3030E+03	2.2951E+03
	Std	3.4698E+01	3.7861E+01	3.4847E+01	3.4904E+01	3.7582E+01
	Rank	5	4	2	3	1
F21	Best	2.3003E+03	2.3009E+03	2.3000E+03	2.3006E+03	2.3000E+03
	Mean	2.3025E+03	2.3025E+03	2.3005E+03	2.3023E+03	2.3001E+03
	Std	1.0934E+00	1.0926E+00	5.2637E−01	7.6575E−01	1.7247E−01
	Rank	4	5	2	3	1
F22	Best	2.6130E+03	2.6085E+03	2.6057E+03	2.6079E+03	2.6031E+03
	Mean	2.6250E+03	2.6221E+03	2.6129E+03	2.6156E+03	2.6119E+03
	Std	9.4900E+00	1.0863E+01	4.7006E+00	4.9025E+00	5.0350E+00
	Rank	5	4	2	3	1
F23	Best	2.7402E+03	2.7380E+03	2.7338E+03	2.5005E+03	2.7317E+03
	Mean	2.7589E+03	2.7589E+03	2.7419E+03	2.7303E+03	2.7390E+03
	Std	1.0265E+01	1.1263E+01	5.5527E+00	6.3099E+01	3.9986E+00
	Rank	4	5	3	1	2
F24	Best	2.8978E+03	2.8981E+03	2.8977E+03	2.8988E+03	2.8977E+03
	Mean	2.9406E+03	2.9416E+03	2.9242E+03	2.9316E+03	2.9273E+03
	Std	2.9959E+01	3.0670E+01	2.3225E+01	2.1735E+01	2.2583E+01
	Rank	4	5	1	3	2
F25	Best	2.9000E+03	2.9000E+03	2.9000E+03	2.8012E+03	2.9000E+03
	Mean	3.1154E+03	3.1197E+03	2.9442E+03	2.9292E+03	2.9032E+03
	Std	3.8076E+02	3.7496E+02	2.1558E+02	5.5831E+01	1.1957E+01
	Rank	4	5	3	2	1
F26	Best	3.0894E+03	3.0890E+03	3.0890E+03	3.0891E+03	3.0890E+03
	Mean	3.0929E+03	3.0931E+03	3.0913E+03	3.0929E+03	3.0901E+03
	Std	2.1748E+00	1.1053E+01	2.3887E+00	1.0979E+01	1.6432E+00
	Rank	3	5	2	4	1
F27	Best	3.1683E+03	3.1664E+03	3.1000E+03	3.1006E+03	3.1000E+03
	Mean	3.3597E+03	3.3624E+03	3.2496E+03	3.3353E+03	3.2457E+03
	Std	9.0449E+01	1.2272E+02	1.4723E+02	1.4384E+02	1.4244E+02
	Rank	4	5	2	3	1
F28	Best	3.1343E+03	3.1390E+03	3.1340E+03	3.1332E+03	3.1293E+03
	Mean	3.2208E+03	3.2095E+03	3.1617E+03	3.1590E+03	3.1404E+03
	Std	5.8366E+01	6.1415E+01	2.2570E+01	2.9912E+01	1.0094E+01
	Rank	5	4	3	2	1
F29	Best	5.0776E+03	5.0063E+03	3.4551E+03	3.5590E+03	3.4600E+03
	Mean	2.6296E+05	2.2230E+05	3.8716E+05	2.5546E+05	2.3768E+05
	Std	5.4917E+05	3.9322E+05	6.5107E+05	4.3565E+05	4.4337E+05
	Rank	4	1	5	3	2

**Table A4:** Results of ablation experiments with BWSMA based on CEC2018 (30D).

No.	Index	SMA	SMA-AGM	SMA-BWS	SMA-SRM	BWSMA
F1	Best	6.7439E+04	2.2583E+03	4.9028E+05	8.1515E+06	1.0241E+02
	Mean	2.4405E+05	1.2736E+04	2.6764E+06	1.1298E+08	3.5430E+03
	Std	8.1541E+04	7.5806E+03	2.7402E+06	1.7839E+08	4.2661E+03
	Rank	3	2	4	5	1
F2	Best	1.0042E+04	7.9119E+03	7.6985E+03	1.5447E+04	3.0103E+02
	Mean	3.5881E+04	2.5994E+04	1.6212E+04	2.6238E+04	3.5108E+02
	Std	1.7853E+04	1.4161E+04	4.9023E+03	7.7037E+03	6.5321E+01
	Rank	5	3	2	4	1
F3	Best	4.7441E+02	4.8884E+02	4.7047E+02	4.9769E+02	4.4193E+02
	Mean	5.1237E+02	5.0324E+02	5.0524E+02	5.4154E+02	4.9308E+02
	Std	2.2657E+01	1.4880E+01	1.4709E+01	3.2096E+01	1.6638E+01
	Rank	4	2	3	5	1
F4	Best	5.7881E+02	5.6331E+02	5.3329E+02	5.4712E+02	5.2105E+02
	Mean	6.3539E+02	6.2221E+02	5.5484E+02	5.8731E+02	5.3446E+02
	Std	3.3258E+01	3.0961E+01	1.5583E+01	2.4522E+01	9.8776E+00
	Rank	5	4	2	3	1
F5	Best	6.0579E+02	6.0344E+02	6.0233E+02	6.0505E+02	6.0009E+02
	Mean	6.1431E+02	6.0810E+02	6.0412E+02	6.0824E+02	6.0027E+02
	Std	7.2943E+00	3.0892E+00	1.2551E+00	2.4450E+00	7.7242E−02
	Rank	5	3	2	4	1
F6	Best	8.3024E+02	7.8920E+02	7.7601E+02	8.1046E+02	7.4556E+02
	Mean	8.8021E+02	8.5414E+02	8.0362E+02	8.5700E+02	7.6442E+02
	Std	3.4858E+01	2.7198E+01	1.9829E+01	2.6410E+01	1.0946E+01
	Rank	5	3	2	4	1
F7	Best	8.7241E+02	8.7087E+02	8.2314E+02	8.4859E+02	8.1328E+02
	Mean	9.2778E+02	9.1591E+02	8.5478E+02	8.8223E+02	8.3911E+02
	Std	3.2725E+01	1.8425E+01	2.0200E+01	1.7381E+01	1.3461E+01
	Rank	5	4	2	3	1
F8	Best	1.9032E+03	2.1906E+03	9.1052E+02	1.2682E+03	9.0020E+02
	Mean	4.5906E+03	4.6876E+03	1.0079E+03	1.7800E+03	9.0142E+02
	Std	1.7384E+03	1.6363E+03	9.6028E+01	3.3481E+02	1.3383E+00
	Rank	4	5	2	3	1
F9	Best	3.8548E+03	2.8270E+03	3.3534E+03	3.7028E+03	3.1575E+03
	Mean	4.8157E+03	4.5420E+03	4.8611E+03	5.2112E+03	4.2796E+03
	Std	6.7942E+02	6.5535E+02	7.8089E+02	7.7753E+02	6.4497E+02
	Rank	3	2	4	5	1
F10	Best	1.1741E+03	1.1575E+03	1.1791E+03	1.2160E+03	1.1099E+03
	Mean	1.2840E+03	1.2960E+03	1.2540E+03	1.3077E+03	1.1536E+03
	Std	5.4767E+01	7.2220E+01	3.7576E+01	5.4904E+01	3.6894E+01
	Rank	3	4	2	5	1
F11	Best	4.0758E+05	7.5587E+05	2.5154E+05	5.7373E+05	8.5647E+03
	Mean	5.5961E+06	4.5173E+06	1.5020E+06	6.9770E+06	3.0646E+04
	Std	4.0940E+06	2.2622E+06	9.5901E+05	6.6525E+06	2.4784E+04
	Rank	4	3	2	5	1
F12	Best	1.6956E+04	7.7280E+03	2.5008E+04	6.0632E+03	1.7429E+03
	Mean	7.0258E+04	3.3481E+04	8.7179E+04	3.4719E+04	3.1906E+03
	Std	4.3349E+04	2.5973E+04	3.7536E+04	2.6800E+04	9.1225E+02
	Rank	4	2	5	3	1
F13	Best	1.4183E+04	1.0933E+04	1.6185E+03	1.7703E+03	1.4351E+03
	Mean	2.5014E+05	2.2363E+05	1.8672E+03	3.2151E+03	1.4590E+03
	Std	2.4023E+05	3.0665E+05	1.7197E+02	2.3679E+03	1.5078E+01
	Rank	5	4	2	3	1
F14	Best	3.0093E+03	1.8594E+03	1.1059E+04	1.7432E+03	1.5974E+03
	Mean	2.1864E+04	2.1082E+04	2.3565E+04	1.9586E+04	1.7426E+03
	Std	1.5677E+04	1.4954E+04	8.3826E+03	1.3128E+04	1.0656E+02
	Rank	4	3	5	2	1
F15	Best	1.9580E+03	2.0555E+03	1.7183E+03	1.9136E+03	1.6057E+03
	Mean	2.6839E+03	2.7636E+03	2.3378E+03	2.4408E+03	2.0826E+03
	Std	3.6369E+02	3.4355E+02	2.5910E+02	3.0942E+02	2.6252E+02
	Rank	4	5	2	3	1
F16	Best	1.9746E+03	1.8326E+03	1.8112E+03	1.8375E+03	1.7596E+03
	Mean	2.3855E+03	2.4637E+03	1.9918E+03	2.0960E+03	1.8777E+03
	Std	2.5220E+02	2.4793E+02	1.5646E+02	1.6796E+02	1.0153E+02
	Rank	4	5	2	3	1
F17	Best	3.2769E+05	2.8243E+05	1.8212E+04	3.6418E+04	1.8850E+03
	Mean	3.3322E+06	3.7023E+06	4.2271E+04	1.1091E+05	1.9892E+03
	Std	3.1667E+06	4.0584E+06	1.5571E+04	5.5042E+04	6.9781E+01
	Rank	4	5	2	3	1
F18	Best	2.1864E+03	1.9710E+03	4.0974E+03	2.3077E+03	1.9250E+03
	Mean	2.5775E+04	2.7607E+04	1.4770E+04	1.8624E+04	1.9746E+03
	Std	2.0354E+04	2.1064E+04	1.1098E+04	1.6668E+04	3.3756E+01
	Rank	4	5	2	3	1
F19	Best	2.1610E+03	2.1569E+03	2.0953E+03	2.1121E+03	2.0303E+03
	Mean	2.5883E+03	2.5468E+03	2.3834E+03	2.4202E+03	2.1999E+03
	Std	2.1724E+02	1.9929E+02	2.0607E+02	2.0190E+02	1.3211E+02
	Rank	5	4	2	3	1
F20	Best	2.3699E+03	2.3803E+03	2.3295E+03	2.3467E+03	2.3225E+03
	Mean	2.4379E+03	2.4263E+03	2.3488E+03	2.3858E+03	2.3367E+03
	Std	3.4957E+01	3.5131E+01	1.5744E+01	1.7120E+01	1.1600E+01
	Rank	5	4	2	3	1
F21	Best	2.3108E+03	2.3032E+03	2.3125E+03	2.3339E+03	2.3003E+03
	Mean	5.8679E+03	5.8762E+03	4.4677E+03	3.4421E+03	4.1865E+03
	Std	1.2259E+03	1.2585E+03	2.0297E+03	1.7667E+03	1.6751E+03
	Rank	4	5	3	1	2
F22	Best	2.6957E+03	2.7103E+03	2.6748E+03	2.6895E+03	2.6735E+03
	Mean	2.7660E+03	2.7685E+03	2.7095E+03	2.7309E+03	2.6919E+03
	Std	2.9491E+01	2.8247E+01	1.8177E+01	1.9791E+01	1.0484E+01
	Rank	4	5	2	3	1
F23	Best	2.8912E+03	2.8694E+03	2.8479E+03	2.8718E+03	2.8324E+03
	Mean	2.9398E+03	2.9336E+03	2.8774E+03	2.9033E+03	2.8571E+03
	Std	3.1492E+01	3.1571E+01	1.7062E+01	1.8967E+01	1.4884E+01
	Rank	5	4	2	3	1
F24	Best	2.8847E+03	2.8879E+03	2.8881E+03	2.8919E+03	2.8870E+03
	Mean	2.8982E+03	2.9016E+03	2.8999E+03	2.9293E+03	2.8907E+03
	Std	1.5124E+01	1.6760E+01	1.1381E+01	2.2353E+01	1.0557E+01
	Rank	2	4	3	5	1
F25	Best	2.9036E+03	2.9013E+03	2.9778E+03	4.2425E+03	2.9014E+03
	Mean	4.9419E+03	4.9095E+03	4.1956E+03	4.7110E+03	3.9298E+03
	Std	7.0619E+02	7.6065E+02	4.2475E+02	2.2883E+02	2.3423E+02
	Rank	5	4	2	3	1
F26	Best	3.1938E+03	3.2003E+03	3.2034E+03	3.1976E+03	3.1903E+03
	Mean	3.2339E+03	3.2289E+03	3.2294E+03	3.2333E+03	3.2092E+03
	Std	2.0646E+01	1.5779E+01	1.4262E+01	1.7641E+01	1.1246E+01
	Rank	5	2	3	4	1
F27	Best	3.2140E+03	3.2174E+03	3.2142E+03	3.2339E+03	3.1515E+03
	Mean	3.2620E+03	3.2594E+03	3.2581E+03	3.3855E+03	3.2208E+03
	Std	3.4961E+01	2.7856E+01	2.3479E+01	3.4102E+02	2.4966E+01
	Rank	4	3	2	5	1
F28	Best	3.5903E+03	3.4780E+03	3.3788E+03	3.4418E+03	3.3168E+03
	Mean	4.0404E+03	3.9055E+03	3.7829E+03	3.7757E+03	3.4787E+03
	Std	2.5825E+02	2.7004E+02	2.0724E+02	1.8634E+02	1.1094E+02
	Rank	5	4	3	2	1
F29	Best	3.6004E+04	8.4586E+03	1.7874E+04	1.2280E+04	6.5387E+03
	Mean	1.0546E+05	2.8231E+04	8.7860E+04	1.0773E+05	9.2945E+03
	Std	1.0724E+05	1.6547E+04	7.2305E+04	1.0449E+05	1.9576E+03
	Rank	4	2	3	5	1

**Table A5:** Results of ablation experiments with BWSMA based on CEC2018 (50D).

No.	Index	SMA	SMA-AGM	SMA-BWS	SMA-SRM	BWSMA
F1	Best	8.2183E+06	2.8479E+05	5.9760E+05	1.1623E+07	2.2080E+04
	Mean	1.2856E+07	7.2394E+05	4.4184E+06	7.7525E+07	2.7179E+05
	Std	4.6592E+06	2.9207E+05	2.7647E+06	8.9826E+07	5.6314E+05
	Rank	4	2	3	5	1
F2	Best	1.2020E+05	7.0953E+04	1.5401E+04	2.0020E+04	5.1564E+03
	Mean	1.8519E+05	1.4168E+05	3.1745E+04	3.2316E+04	1.0326E+04
	Std	7.5353E+04	5.4006E+04	9.5936E+03	9.1631E+03	3.8925E+03
	Rank	5	4	2	3	1
F3	Best	4.6962E+02	5.0681E+02	5.4087E+02	4.8423E+02	5.0326E+02
	Mean	6.0014E+02	6.1961E+02	6.0212E+02	6.1931E+02	5.5899E+02
	Std	5.6401E+01	5.9190E+01	2.9190E+01	4.7603E+01	4.3939E+01
	Rank	2	5	3	4	1
F4	Best	7.3706E+02	7.0206E+02	5.4738E+02	6.2767E+02	5.4801E+02
	Mean	7.8933E+02	7.7741E+02	5.8949E+02	6.6198E+02	5.7606E+02
	Std	3.4825E+01	5.0289E+01	2.6423E+01	2.2716E+01	1.7387E+01
	Rank	5	4	2	3	1
F5	Best	6.2216E+02	6.1673E+02	6.0303E+02	6.0772E+02	6.0112E+02
	Mean	6.4172E+02	6.3936E+02	6.0518E+02	6.1033E+02	6.0182E+02
	Std	1.5086E+01	1.3841E+01	1.4292E+00	2.3668E+00	5.9044E−01
	Rank	5	4	2	3	1
F6	Best	9.7074E+02	9.6898E+02	8.1512E+02	8.9816E+02	7.9464E+02
	Mean	1.0838E+03	1.0650E+03	8.5568E+02	9.6941E+02	8.3589E+02
	Std	6.2668E+01	4.9898E+01	2.0298E+01	4.7288E+01	1.8005E+01
	Rank	5	4	2	3	1
F7	Best	1.0177E+03	9.8811E+02	8.7311E+02	9.0091E+02	8.5654E+02
	Mean	1.1196E+03	1.0552E+03	8.9317E+02	9.5757E+02	8.8566E+02
	Std	6.1274E+01	4.2631E+01	1.3360E+01	3.5032E+01	1.7938E+01
	Rank	5	4	2	3	1
F8	Best	9.6214E+03	8.5137E+03	9.4608E+02	1.2156E+03	9.1860E+02
	Mean	1.7114E+04	1.5143E+04	1.1975E+03	2.4407E+03	1.0031E+03
	Std	4.5611E+03	3.5521E+03	1.9382E+02	6.4226E+02	7.9316E+01
	Rank	5	4	2	3	1
F9	Best	5.5925E+03	6.0820E+03	5.5637E+03	7.5996E+03	5.4488E+03
	Mean	8.0296E+03	7.3215E+03	7.9025E+03	9.0716E+03	6.7879E+03
	Std	1.2121E+03	6.9510E+02	1.1477E+03	9.9591E+02	9.5459E+02
	Rank	4	2	3	5	1
F10	Best	1.3516E+03	1.3371E+03	1.2537E+03	1.3450E+03	1.1790E+03
	Mean	1.4461E+03	1.4262E+03	1.3369E+03	1.4497E+03	1.2767E+03
	Std	4.8539E+01	8.2728E+01	4.5767E+01	4.5519E+01	5.1713E+01
	Rank	4	3	2	5	1
F11	Best	8.2005E+06	7.6849E+06	2.6098E+06	3.1245E+06	1.7229E+05
	Mean	3.7223E+07	3.1027E+07	5.6827E+06	2.6682E+07	1.4553E+06
	Std	2.6893E+07	1.7743E+07	2.8291E+06	2.6057E+07	1.6432E+06
	Rank	5	4	2	3	1
F12	Best	4.6341E+04	2.2802E+04	1.8597E+04	4.7165E+03	9.5964E+03
	Mean	1.5881E+05	5.9807E+04	5.3175E+04	2.5774E+04	2.0906E+04
	Std	9.3827E+04	2.2093E+04	2.6815E+04	1.5847E+04	5.8453E+03
	Rank	5	4	3	2	1
F13	Best	1.5568E+05	1.3038E+05	2.0134E+03	1.8294E+03	1.5925E+03
	Mean	1.0541E+06	6.6633E+05	2.2200E+03	2.7247E+03	1.6316E+03
	Std	7.8127E+05	4.3652E+05	1.6232E+02	5.3196E+02	4.5953E+01
	Rank	5	4	2	3	1
F14	Best	7.9972E+03	5.8670E+03	1.0982E+04	1.8788E+03	2.3777E+03
	Mean	4.0842E+04	2.2011E+04	2.2232E+04	2.1569E+04	3.0764E+03
	Std	2.6657E+04	1.0955E+04	8.8148E+03	1.3590E+04	4.9527E+02
	Rank	5	3	4	2	1
F15	Best	2.9519E+03	3.0565E+03	2.3151E+03	2.5691E+03	2.1960E+03
	Mean	3.7503E+03	3.6921E+03	2.9145E+03	2.9431E+03	2.8367E+03
	Std	4.2168E+02	4.7714E+02	3.1752E+02	2.2359E+02	3.6862E+02
	Rank	5	4	2	3	1
F16	Best	2.8413E+03	2.9096E+03	2.1692E+03	2.2362E+03	2.3598E+03
	Mean	3.3775E+03	3.3524E+03	2.6178E+03	2.8262E+03	2.6411E+03
	Std	3.3740E+02	3.1984E+02	2.9819E+02	2.1980E+02	1.6929E+02
	Rank	5	4	1	3	2
F17	Best	3.5370E+05	1.4022E+06	1.9531E+04	4.1169E+04	2.5336E+03
	Mean	8.5300E+06	6.1442E+06	3.8483E+04	7.9007E+04	4.7529E+03
	Std	7.0700E+06	3.8511E+06	1.2048E+04	2.1625E+04	2.4743E+03
	Rank	5	4	2	3	1
F18	Best	5.1009E+03	2.6364E+03	2.9996E+03	2.2017E+03	2.0305E+03
	Mean	1.9546E+04	1.1189E+04	1.2251E+04	1.1914E+04	2.3563E+03
	Std	1.6310E+04	1.2189E+04	9.5490E+03	1.1647E+04	5.1207E+02
	Rank	5	2	4	3	1
F19	Best	2.6298E+03	2.6451E+03	2.1832E+03	2.5961E+03	2.2358E+03
	Mean	3.2557E+03	3.3260E+03	2.8912E+03	3.0051E+03	2.6872E+03
	Std	3.7625E+02	3.0929E+02	3.3499E+02	2.5498E+02	3.3124E+02
	Rank	4	5	2	3	1
F20	Best	2.4890E+03	2.5074E+03	2.3384E+03	2.4002E+03	2.3525E+03
	Mean	2.5799E+03	2.5613E+03	2.3761E+03	2.4459E+03	2.3787E+03
	Std	5.5410E+01	5.9196E+01	2.0881E+01	2.1252E+01	1.8977E+01
	Rank	5	4	1	3	2
F21	Best	7.1981E+03	7.3031E+03	7.1930E+03	8.7972E+03	6.6908E+03
	Mean	9.2642E+03	8.9452E+03	9.1413E+03	1.0288E+04	8.3897E+03
	Std	9.5940E+02	1.1444E+03	1.1486E+03	6.8626E+02	1.1551E+03
	Rank	4	2	3	5	1
F22	Best	2.9641E+03	2.9102E+03	2.7806E+03	2.8353E+03	2.7757E+03
	Mean	3.0372E+03	3.0222E+03	2.8229E+03	2.8882E+03	2.8072E+03
	Std	5.8050E+01	7.1554E+01	2.5725E+01	3.3025E+01	1.7922E+01
	Rank	5	4	2	3	1
F23	Best	3.0653E+03	3.0887E+03	2.9609E+03	2.9849E+03	2.9318E+03
	Mean	3.1665E+03	3.1582E+03	2.9776E+03	3.0289E+03	2.9672E+03
	Std	5.6220E+01	8.2456E+01	1.7550E+01	2.2846E+01	2.0565E+01
	Rank	5	4	2	3	1
F24	Best	3.0647E+03	3.0100E+03	3.0291E+03	3.0625E+03	2.9855E+03
	Mean	3.1140E+03	3.0660E+03	3.0507E+03	3.1225E+03	3.0392E+03
	Std	3.2669E+01	3.5213E+01	1.8340E+01	5.2224E+01	2.4640E+01
	Rank	4	3	2	5	1
F25	Best	2.9544E+03	3.2925E+03	4.0930E+03	4.6883E+03	4.2395E+03
	Mean	5.7661E+03	6.2563E+03	4.5688E+03	5.2760E+03	4.5684E+03
	Std	2.3239E+03	1.2044E+03	2.2997E+02	3.5387E+02	2.6927E+02
	Rank	4	5	2	3	1
F26	Best	3.3376E+03	3.3883E+03	3.2843E+03	3.3181E+03	3.2423E+03
	Mean	3.5268E+03	3.4819E+03	3.3522E+03	3.4269E+03	3.3114E+03
	Std	1.1761E+02	7.2070E+01	5.6175E+01	6.7091E+01	6.3983E+01
	Rank	5	4	2	3	1
F27	Best	3.3225E+03	3.3053E+03	3.2628E+03	3.3349E+03	3.2625E+03
	Mean	3.3769E+03	3.3716E+03	3.3194E+03	3.5799E+03	3.2966E+03
	Std	4.6650E+01	5.3085E+01	3.6726E+01	2.5394E+02	2.4399E+01
	Rank	4	3	2	5	1
F28	Best	4.5063E+03	4.0633E+03	3.4389E+03	3.5108E+03	3.6198E+03
	Mean	5.0444E+03	4.8017E+03	4.1870E+03	4.1645E+03	3.9003E+03
	Std	3.0683E+02	4.0681E+02	3.2938E+02	2.8621E+02	1.8966E+02
	Rank	5	4	3	2	1
F29	Best	5.2654E+06	1.4821E+06	2.1609E+06	1.1233E+06	1.5738E+06
	Mean	9.3141E+06	3.2614E+06	3.7768E+06	4.0330E+06	2.2526E+06
	Std	3.0736E+06	1.2900E+06	1.3681E+06	2.7910E+06	4.1165E+05
	Rank	5	2	3	4	1

**Table A6:** Results of ablation experiments with BWSMA based on CEC2018 (100D).

No.	Index	SMA	SMA-AGM	SMA-BWS	SMA-SRM	BWSMA
F1	Best	2.9335E+08	6.1175E+07	3.3174E+08	2.7694E+09	7.0450E+07
	Mean	6.1449E+08	1.0262E+08	7.4634E+08	6.7584E+09	1.9438E+08
	Std	1.4285E+08	3.3841E+07	2.7285E+08	3.3645E+09	5.6174E+07
	Rank	3	1	4	5	2
F2	Best	3.6324E+05	2.9355E+05	2.6784E+05	1.7191E+05	1.3985E+05
	Mean	9.3939E+05	6.9383E+05	3.0176E+05	2.2057E+05	1.5957E+05
	Std	2.4631E+05	3.3450E+05	2.6071E+04	2.5909E+04	1.8424E+04
	Rank	5	4	3	2	1
F3	Best	9.2617E+02	7.5527E+02	8.4046E+02	1.0052E+03	7.6371E+02
	Mean	1.0117E+03	8.7867E+02	9.7985E+02	1.2733E+03	8.5964E+02
	Std	5.9868E+01	5.7837E+01	7.6284E+01	1.5521E+02	5.7241E+01
	Rank	4	2	3	5	1
F4	Best	1.2153E+03	1.1507E+03	7.7038E+02	9.4583E+02	7.2204E+02
	Mean	1.3500E+03	1.2694E+03	8.7054E+02	1.0297E+03	8.0471E+02
	Std	1.0779E+02	6.9625E+01	5.1830E+01	5.0712E+01	3.7713E+01
	Rank	5	4	2	3	1
F5	Best	6.4928E+02	6.4046E+02	6.2356E+02	6.2225E+02	6.1293E+02
	Mean	6.6278E+02	6.5564E+02	6.2800E+02	6.3251E+02	6.1802E+02
	Std	6.4471E+00	7.6191E+00	2.5103E+00	5.0945E+00	3.4028E+00
	Rank	5	4	2	3	1
F6	Best	1.9039E+03	1.8034E+03	1.3258E+03	1.5294E+03	1.2112E+03
	Mean	2.1697E+03	2.0658E+03	1.5129E+03	1.7287E+03	1.3253E+03
	Std	1.8561E+02	1.9328E+02	8.0094E+01	1.0803E+02	6.2628E+01
	Rank	5	4	2	3	1
F7	Best	1.5448E+03	1.4227E+03	1.1313E+03	1.2214E+03	1.0353E+03
	Mean	1.6851E+03	1.5787E+03	1.1874E+03	1.3127E+03	1.0991E+03
	Std	8.2218E+01	9.9907E+01	4.0329E+01	6.8417E+01	3.7806E+01
	Rank	5	4	2	3	1
F8	Best	3.3315E+04	2.7073E+04	5.9457E+03	9.0112E+03	3.8381E+03
	Mean	3.9850E+04	3.4222E+04	8.2614E+03	2.2512E+04	6.0605E+03
	Std	4.0371E+03	4.9529E+03	1.3150E+03	7.7692E+03	2.0907E+03
	Rank	5	4	2	3	1
F9	Best	1.5562E+04	1.3387E+04	1.5699E+04	1.6224E+04	1.5098E+04
	Mean	1.8444E+04	1.6680E+04	1.8014E+04	2.0501E+04	1.6738E+04
	Std	1.4215E+03	1.9066E+03	1.8078E+03	2.6631E+03	1.3312E+03
	Rank	4	1	3	5	2
F10	Best	1.3188E+04	1.6800E+04	1.0084E+04	3.8439E+03	3.2800E+03
	Mean	3.5506E+04	3.1681E+04	1.5490E+04	6.4362E+03	4.4914E+03
	Std	1.5518E+04	1.4828E+04	2.2524E+03	2.1344E+03	8.8862E+02
	Rank	5	4	3	2	1
F11	Best	1.6072E+08	7.3803E+07	9.0859E+07	2.1048E+08	4.1783E+07
	Mean	3.9468E+08	2.1963E+08	1.9447E+08	6.7318E+08	9.9655E+07
	Std	1.5571E+08	1.0086E+08	7.0554E+07	3.7604E+08	4.1968E+07
	Rank	4	3	2	5	1
F12	Best	1.3116E+05	4.5573E+04	6.5328E+04	2.5624E+04	4.3968E+04
	Mean	3.1379E+06	5.3748E+05	1.4098E+05	6.7967E+04	6.2741E+04
	Std	1.0416E+07	1.1832E+06	5.8255E+04	6.1766E+04	1.0634E+04
	Rank	5	4	3	2	1
F13	Best	2.3864E+06	1.6864E+06	1.3525E+05	6.0192E+04	6.6748E+03
	Mean	5.0827E+06	5.3299E+06	3.2445E+05	3.3047E+05	2.6564E+04
	Std	2.8888E+06	4.1994E+06	1.0532E+05	1.7111E+05	2.4931E+04
	Rank	4	5	2	3	1
F14	Best	6.0233E+04	3.5023E+04	5.0827E+04	4.9362E+03	1.0864E+04
	Mean	7.9729E+05	3.9549E+05	9.0593E+04	8.0804E+04	2.4456E+04
	Std	2.0219E+06	6.3747E+05	3.0032E+04	2.4914E+05	9.9346E+03
	Rank	5	4	3	2	1
F15	Best	4.9391E+03	5.0057E+03	4.4450E+03	4.1776E+03	4.5656E+03
	Mean	6.6359E+03	5.9270E+03	5.6523E+03	5.9308E+03	5.4122E+03
	Std	8.6079E+02	5.4788E+02	5.1037E+02	8.4544E+02	5.0119E+02
	Rank	5	3	2	4	1
F16	Best	4.3427E+03	4.7909E+03	4.2258E+03	3.7957E+03	3.4256E+03
	Mean	5.6436E+03	5.7837E+03	4.8933E+03	4.6775E+03	4.4578E+03
	Std	6.3468E+02	6.0216E+02	4.0687E+02	4.1494E+02	5.1992E+02
	Rank	4	5	3	2	1
F17	Best	4.8225E+06	3.2832E+06	3.2128E+05	3.1771E+05	3.9945E+04
	Mean	1.0047E+07	1.2209E+07	5.3833E+05	7.5870E+05	8.4975E+04
	Std	5.1570E+06	4.2439E+06	1.4655E+05	3.5742E+05	2.2609E+04
	Rank	4	5	2	3	1
F18	Best	1.4745E+05	1.8794E+04	2.7676E+04	4.5420E+03	6.1620E+03
	Mean	5.1427E+05	5.7059E+04	1.5172E+05	4.5762E+04	5.1381E+04
	Std	3.4018E+05	6.9333E+04	8.4132E+04	8.6485E+04	6.8858E+04
	Rank	5	3	4	1	2
F19	Best	4.7325E+03	4.7012E+03	4.0084E+03	3.5119E+03	4.0953E+03
	Mean	5.6316E+03	5.6474E+03	5.0096E+03	4.7446E+03	4.7803E+03
	Std	6.0655E+02	4.3625E+02	5.7107E+02	5.9582E+02	4.4110E+02
	Rank	4	5	3	1	2
F20	Best	3.0111E+03	3.0150E+03	2.6000E+03	2.7235E+03	2.5563E+03
	Mean	3.1982E+03	3.1252E+03	2.7139E+03	2.8302E+03	2.6131E+03
	Std	1.3199E+02	9.1378E+01	5.6976E+01	4.9585E+01	4.5271E+01
	Rank	5	4	2	3	1
F21	Best	1.8213E+04	1.6996E+04	1.7409E+04	1.8614E+04	1.7145E+04
	Mean	2.1446E+04	1.9405E+04	2.0236E+04	2.2924E+04	1.9601E+04
	Std	1.6005E+03	1.1228E+03	1.9280E+03	2.6962E+03	1.4572E+03
	Rank	4	1	3	5	2
F22	Best	3.4530E+03	3.2757E+03	3.1496E+03	3.1483E+03	3.0796E+03
	Mean	3.5663E+03	3.4255E+03	3.2539E+03	3.2321E+03	3.1696E+03
	Std	7.4896E+01	7.9695E+01	6.3334E+01	5.6727E+01	4.1180E+01
	Rank	5	4	3	2	1
F23	Best	4.0054E+03	3.8992E+03	3.6334E+03	3.6694E+03	3.5192E+03
	Mean	4.2415E+03	4.0220E+03	3.7328E+03	3.7755E+03	3.6361E+03
	Std	1.5605E+02	8.1787E+01	5.0837E+01	6.4949E+01	4.7330E+01
	Rank	5	4	2	3	1
F24	Best	3.5860E+03	3.4276E+03	3.6184E+03	3.8896E+03	3.4140E+03
	Mean	3.6867E+03	3.5996E+03	3.7191E+03	4.1964E+03	3.5716E+03
	Std	8.0200E+01	9.1593E+01	7.2304E+01	2.7611E+02	7.2742E+01
	Rank	3	2	4	5	1
F25	Best	5.4651E+03	1.1827E+04	9.2849E+03	9.8766E+03	8.8280E+03
	Mean	1.4782E+04	1.3894E+04	1.0594E+04	1.1010E+04	9.4549E+03
	Std	2.9616E+03	1.2148E+03	5.9529E+02	6.7521E+02	4.7181E+02
	Rank	5	4	2	3	1
F26	Best	3.5984E+03	3.4979E+03	3.5013E+03	3.4836E+03	3.4289E+03
	Mean	3.7580E+03	3.6221E+03	3.6150E+03	3.5923E+03	3.5420E+03
	Std	8.4152E+01	6.8361E+01	7.9835E+01	8.0731E+01	5.5613E+01
	Rank	5	4	3	2	1
F27	Best	3.6185E+03	3.5844E+03	3.6672E+03	4.0497E+03	3.5928E+03
	Mean	3.7325E+03	3.6287E+03	3.8290E+03	4.6433E+03	3.6984E+03
	Std	7.4455E+01	3.6295E+01	1.5112E+02	5.0183E+02	7.5370E+01
	Rank	3	1	4	5	2
F28	Best	6.9713E+03	6.2396E+03	6.4695E+03	5.8348E+03	5.5167E+03
	Mean	8.1562E+03	7.4903E+03	7.3101E+03	6.8188E+03	6.6386E+03
	Std	6.8176E+02	8.2938E+02	6.8984E+02	4.4825E+02	6.6864E+02
	Rank	5	4	3	2	1
F29	Best	2.9704E+06	6.6771E+05	2.2302E+06	1.3346E+06	3.3834E+05
	Mean	1.1563E+07	2.5856E+06	5.4763E+06	6.7743E+06	1.1179E+06
	Std	5.1551E+06	1.3197E+06	2.9529E+06	4.7646E+06	7.1226E+05
	Rank	5	2	3	4	1

**Table A7:** Results of ablation experiments with BWSMA based on CEC2022 (10D).

No.	Index	SMA	SMA-AGM	SMA-BWS	SMA-SRM	BWSMA
F1	Best	3.0000E+02	3.0000E+02	3.0000E+02	3.0006E+02	3.0000E+02
	Mean	3.0006E+02	3.0001E+02	3.0000E+02	3.0136E+02	3.0000E+02
	Std	6.7015E−02	1.7061E−02	5.3818E−04	1.3789E+00	1.3704E−03
	Rank	4	3	1	5	2
F2	Best	4.0026E+02	4.0628E+02	4.0001E+02	4.0028E+02	4.0002E+02
	Mean	4.1030E+02	4.1272E+02	4.0721E+02	4.0716E+02	4.0649E+02
	Std	1.5297E+01	1.7776E+01	2.6036E+00	2.0174E+00	2.8829E+00
	Rank	4	5	3	2	1
F3	Best	6.0009E+02	6.0008E+02	6.0003E+02	6.0024E+02	6.0000E+02
	Mean	6.0032E+02	6.0016E+02	6.0009E+02	6.0050E+02	6.0002E+02
	Std	4.0248E−01	5.2959E−02	2.5504E−02	1.1786E−01	1.9725E−02
	Rank	4	3	2	5	1
F4	Best	8.1294E+02	8.1194E+02	8.0199E+02	8.0506E+02	8.0298E+02
	Mean	8.3131E+02	8.2963E+02	8.0945E+02	8.1519E+02	8.0813E+02
	Std	1.3203E+01	1.0735E+01	5.1254E+00	6.8395E+00	3.4574E+00
	Rank	5	4	2	3	1
F5	Best	9.0001E+02	9.0000E+02	9.0000E+02	9.0008E+02	9.0000E+02
	Mean	9.4552E+02	9.1954E+02	9.0003E+02	9.0104E+02	9.0001E+02
	Std	1.2699E+02	5.5046E+01	1.1542E−01	1.6423E+00	2.2714E−02
	Rank	5	4	2	3	1
F6	Best	1.8662E+03	1.9092E+03	1.8058E+03	1.8814E+03	1.8002E+03
	Mean	4.6717E+03	4.6491E+03	1.8529E+03	3.4391E+03	1.8130E+03
	Std	1.9392E+03	2.0367E+03	3.1883E+01	2.0096E+03	1.1076E+01
	Rank	5	4	2	3	1
F7	Best	2.0011E+03	2.0020E+03	2.0017E+03	2.0073E+03	2.0000E+03
	Mean	2.0197E+03	2.0198E+03	2.0224E+03	2.0215E+03	2.0163E+03
	Std	5.7652E+00	4.7928E+00	9.0545E+00	3.5909E+00	8.6227E+00
	Rank	2	3	5	4	1
F8	Best	2.2203E+03	2.2201E+03	2.2015E+03	2.2017E+03	2.2002E+03
	Mean	2.2213E+03	2.2213E+03	2.2204E+03	2.2193E+03	2.2138E+03
	Std	8.2023E−01	9.3656E−01	5.6025E+00	7.1824E+00	9.4827E+00
	Rank	5	4	3	2	1
F9	Best	2.5293E+03	2.5293E+03	2.5293E+03	2.5293E+03	2.5293E+03
	Mean	2.5293E+03	2.5293E+03	2.5293E+03	2.5293E+03	2.5293E+03
	Std	1.7370E−03	7.2037E−04	5.6549E−04	1.3252E−02	1.1269E−04
	Rank	4	3	2	5	1
F10	Best	2.5002E+03	2.5003E+03	2.5002E+03	2.5004E+03	2.5002E+03
	Mean	2.5228E+03	2.5495E+03	2.5225E+03	2.5270E+03	2.5225E+03
	Std	5.1515E+01	6.1680E+01	4.5112E+01	4.8908E+01	4.5283E+01
	Rank	3	5	2	4	1
F11	Best	2.6001E+03	2.6000E+03	2.6000E+03	2.6006E+03	2.6000E+03
	Mean	2.7716E+03	2.7302E+03	2.7101E+03	2.6985E+03	2.7400E+03
	Std	1.8946E+02	1.4058E+02	1.3610E+02	1.6760E+02	1.5222E+02
	Rank	5	3	2	1	4
F12	Best	2.8597E+03	2.8596E+03	2.8594E+03	2.8614E+03	2.8594E+03
	Mean	2.8633E+03	2.8628E+03	2.8634E+03	2.8624E+03	2.8631E+03
	Std	1.3500E+00	1.2611E+00	1.6675E+00	1.0167E+00	1.5168E+00
	Rank	4	2	5	1	3

**Table A8:** Results of ablation experiments with BWSMA based on CEC2022 (20D).

No.	Index	SMA	SMA-AGM	SMA-BWS	SMA-SRM	BWSMA
F1	Best	3.6330E+02	3.2140E+02	3.3457E+02	7.0865E+02	3.0007E+02
	Mean	2.4384E+03	1.5993E+03	6.6314E+02	1.9003E+03	3.0044E+02
	Std	1.7939E+03	1.5122E+03	3.1066E+02	1.1377E+03	2.9651E−01
	Rank	5	3	2	4	1
F2	Best	4.0651E+02	4.4494E+02	4.0614E+02	4.4543E+02	4.4490E+02
	Mean	4.6788E+02	4.5922E+02	4.4851E+02	4.5697E+02	4.5129E+02
	Std	4.1839E+01	2.1495E+01	1.1992E+01	2.1808E+01	7.9738E+00
	Rank	5	4	1	3	2
F3	Best	6.0080E+02	6.0062E+02	6.0073E+02	6.0159E+02	6.0004E+02
	Mean	6.0477E+02	6.0186E+02	6.0108E+02	6.0265E+02	6.0012E+02
	Std	4.1965E+00	1.2873E+00	2.7204E−01	8.8362E−01	4.7548E−02
	Rank	5	3	2	4	1
F4	Best	8.2696E+02	8.4387E+02	8.1111E+02	8.1434E+02	8.0996E+02
	Mean	8.8920E+02	8.8459E+02	8.2343E+02	8.4273E+02	8.1862E+02
	Std	3.2891E+01	2.3304E+01	8.5538E+00	1.5012E+01	7.9788E+00
	Rank	5	4	2	3	1
F5	Best	1.0131E+03	1.0384E+03	9.0018E+02	9.1043E+02	9.0000E+02
	Mean	1.9123E+03	1.8509E+03	9.0585E+02	1.0188E+03	9.0010E+02
	Std	7.1176E+02	7.1082E+02	4.8808E+00	9.5581E+01	1.1172E−01
	Rank	5	4	2	3	1
F6	Best	2.0474E+03	2.0387E+03	3.7582E+03	2.0255E+03	1.8632E+03
	Mean	1.9453E+04	1.8429E+04	1.1103E+04	1.7173E+04	1.9348E+03
	Std	7.1187E+03	8.4444E+03	4.2549E+03	7.9368E+03	6.9540E+01
	Rank	5	4	2	3	1
F7	Best	2.0264E+03	2.0256E+03	2.0269E+03	2.0277E+03	2.0024E+03
	Mean	2.0870E+03	2.0836E+03	2.0529E+03	2.0519E+03	2.0253E+03
	Std	4.4533E+01	3.1829E+01	1.7438E+01	2.7550E+01	7.2348E+00
	Rank	5	4	3	2	1
F8	Best	2.2237E+03	2.2215E+03	2.2235E+03	2.2235E+03	2.2082E+03
	Mean	2.2691E+03	2.2550E+03	2.2318E+03	2.2262E+03	2.2223E+03
	Std	4.9492E+01	4.9085E+01	2.1404E+01	1.4406E+00	2.9836E+00
	Rank	5	4	3	2	1
F9	Best	2.4808E+03	2.4808E+03	2.4808E+03	2.4810E+03	2.4808E+03
	Mean	2.4815E+03	2.4812E+03	2.4809E+03	2.4824E+03	2.4808E+03
	Std	9.8731E−01	4.9868E−01	6.4140E−02	1.6644E+00	1.3075E−02
	Rank	4	3	2	5	1
F10	Best	2.5006E+03	2.5007E+03	2.5004E+03	2.5006E+03	2.5003E+03
	Mean	3.0343E+03	2.9643E+03	3.4922E+03	3.2711E+03	2.8591E+03
	Std	3.7503E+02	2.5676E+02	9.2910E+02	5.7995E+02	3.7931E+02
	Rank	3	2	5	4	1
F11	Best	2.6056E+03	2.6016E+03	2.9023E+03	2.6857E+03	2.9001E+03
	Mean	2.9821E+03	2.9170E+03	2.9093E+03	3.1823E+03	2.9007E+03
	Std	1.9050E+02	1.2221E+02	3.9249E+00	3.0207E+02	3.6095E−01
	Rank	4	3	2	5	1
F12	Best	2.9383E+03	2.9377E+03	2.9361E+03	2.9401E+03	2.9320E+03
	Mean	2.9526E+03	2.9495E+03	2.9476E+03	2.9504E+03	2.9413E+03
	Std	1.5183E+01	1.5834E+01	9.6213E+00	1.0136E+01	7.2332E+00
	Rank	5	3	2	4	1

**Table A9:** Comparison results between BWSMA and SMA variants based on CEC2018 (10D).

No.	Index	BWSMA	PSMADE	MSSMA	ESMA	LSMA	AOSMA	EOSMA	ISMA	DTSMA
F1	Best	1.1525E+02	1.0000E+02	1.0480E+02	3.3766E+02	1.0106E+02	2.2654E+02	1.0409E+05	1.0047E+02	1.5864E+02
	Mean	9.2922E+02	1.1348E+03	5.2064E+03	5.9063E+03	6.0436E+03	5.9570E+03	6.8593E+05	2.5497E+03	5.0336E+03
	Std	7.5662E+02	2.9113E+03	3.7387E+03	4.2704E+03	4.4007E+03	4.5631E+03	7.4809E+05	2.4183E+03	3.7765E+03
	Rank	1	2	5	6	8	7	9	3	4
F2	Best	3.0000E+02	3.0000E+02	3.0001E+02	3.0001E+02	3.0000E+02	3.0010E+02	1.3481E+03	3.0313E+02	3.0000E+02
	Mean	3.0000E+02	4.4092E+02	3.0185E+02	3.1096E+02	3.0044E+02	3.4406E+02	3.3211E+03	3.1842E+02	3.1303E+02
	Std	1.6833E−03	5.0798E+02	5.5960E+00	2.6013E+01	1.0952E+00	6.4518E+01	1.3413E+03	1.7765E+01	2.6402E+01
	Rank	1	8	3	4	2	7	9	6	5
F3	Best	4.0080E+02	4.0000E+02	4.0040E+02	4.0379E+02	4.0398E+02	4.0270E+02	4.0522E+02	4.0017E+02	4.0036E+02
	Mean	4.0420E+02	4.0949E+02	4.1175E+02	4.1372E+02	4.1135E+02	4.1470E+02	4.0707E+02	4.1593E+02	4.1412E+02
	Std	1.1763E+00	2.2113E+01	1.7780E+01	2.1449E+01	1.6796E+01	2.1458E+01	7.3466E−01	2.5399E+01	2.2843E+01
	Rank	1	3	5	6	4	8	2	9	7
F4	Best	5.0299E+02	5.0497E+02	5.0882E+02	5.0796E+02	5.0597E+02	5.0703E+02	5.0713E+02	5.0995E+02	5.0597E+02
	Mean	5.0914E+02	5.2020E+02	5.2191E+02	5.2122E+02	5.2149E+02	5.3625E+02	5.1654E+02	5.2747E+02	5.1784E+02
	Std	3.7821E+00	8.3928E+00	8.3356E+00	7.2438E+00	9.8673E+00	1.9161E+01	4.9419E+00	1.1378E+01	6.2010E+00
	Rank	1	4	7	5	6	9	2	8	3
F5	Best	6.0000E+02	6.0026E+02	6.0005E+02	6.0003E+02	6.0002E+02	6.0025E+02	6.0006E+02	6.0072E+02	6.0001E+02
	Mean	6.0002E+02	6.0052E+02	6.0062E+02	6.0014E+02	6.0048E+02	6.0911E+02	6.0044E+02	6.0696E+02	6.0022E+02
	Std	2.4785E−02	2.7202E−01	8.6464E−01	9.7639E−02	7.3214E−01	7.3403E+00	3.1397E−01	6.0355E+00	1.6123E−01
	Rank	1	6	7	2	5	9	4	8	3
F6	Best	7.1320E+02	7.1506E+02	7.1362E+02	7.1326E+02	7.1541E+02	7.2759E+02	7.2306E+02	7.2427E+02	7.1041E+02
	Mean	7.1869E+02	7.3256E+02	7.4629E+02	7.3046E+02	7.2834E+02	7.5537E+02	7.3222E+02	7.5322E+02	7.2609E+02
	Std	3.2076E+00	1.1198E+01	1.4137E+01	1.1355E+01	8.6943E+00	2.3183E+01	7.4905E+00	1.6527E+01	7.1927E+00
	Rank	1	6	7	4	3	9	5	8	2
F7	Best	8.0199E+02	8.0995E+02	8.1194E+02	8.0995E+02	8.0597E+02	8.1194E+02	8.0580E+02	8.1194E+02	8.0597E+02
	Mean	8.0876E+02	8.2044E+02	8.2443E+02	8.2086E+02	8.2371E+02	8.2979E+02	8.1481E+02	8.2412E+02	8.1625E+02
	Std	3.7990E+00	7.0319E+00	6.6665E+00	8.3917E+00	1.0377E+01	1.1545E+01	5.1266E+00	6.6350E+00	6.3880E+00
	Rank	1	4	8	5	6	9	2	7	3
F8	Best	9.0000E+02	9.0000E+02	9.0000E+02	9.0000E+02	9.0000E+02	9.0540E+02	9.0003E+02	9.0046E+02	9.0000E+02
	Mean	9.0000E+02	9.0442E+02	9.5771E+02	9.0567E+02	9.0754E+02	1.1309E+03	9.0114E+02	9.7620E+02	9.0093E+02
	Std	1.6351E−02	7.0758E+00	1.0014E+02	1.7355E+01	2.2059E+01	2.0473E+02	9.6746E−01	1.1078E+02	1.6285E+00
	Rank	1	4	7	5	6	9	3	8	2
F9	Best	1.0103E+03	1.1389E+03	1.1451E+03	1.1465E+03	1.2050E+03	1.1431E+03	1.4580E+03	1.1286E+03	1.3532E+03
	Mean	1.3616E+03	1.6731E+03	1.6486E+03	1.6792E+03	1.6947E+03	1.9624E+03	2.0234E+03	1.9231E+03	1.5548E+03
	Std	2.1647E+02	2.9115E+02	2.8148E+02	3.4063E+02	2.5951E+02	3.6337E+02	2.6822E+02	3.1313E+02	1.7767E+02
	Rank	1	4	3	5	6	8	9	7	2
F10	Best	1.1003E+03	1.1030E+03	1.1018E+03	1.1024E+03	1.1030E+03	1.1114E+03	1.1054E+03	1.1065E+03	1.1027E+03
	Mean	1.1033E+03	1.1299E+03	1.1141E+03	1.1629E+03	1.2100E+03	1.1842E+03	1.1161E+03	1.1556E+03	1.1116E+03
	Std	1.4183E+00	6.2013E+01	6.7529E+00	9.1142E+01	1.0595E+02	8.7463E+01	1.0388E+01	6.5645E+01	6.6829E+00
	Rank	1	5	3	7	9	8	4	6	2
F11	Best	1.3188E+03	1.2002E+03	8.4906E+03	7.7630E+03	4.8931E+04	5.3783E+03	4.7938E+04	2.2102E+04	3.1000E+03
	Mean	4.7612E+03	5.5029E+04	1.1003E+06	6.4135E+05	1.8834E+06	1.8176E+06	5.2268E+05	1.5507E+06	4.8607E+04
	Std	8.4062E+03	2.2660E+05	1.3296E+06	5.8337E+05	1.7870E+06	2.0472E+06	6.2716E+05	1.5903E+06	5.9313E+04
	Rank	1	3	6	5	9	8	4	7	2
F12	Best	1.3032E+03	1.3032E+03	1.3407E+03	1.3118E+03	2.3084E+03	2.8489E+03	2.1155E+03	2.2187E+03	1.6455E+03
	Mean	1.3120E+03	3.3078E+03	7.5088E+03	1.0260E+04	1.6716E+04	1.8833E+04	4.5519E+03	2.0517E+04	1.1988E+04
	Std	5.8979E+00	5.0804E+03	8.4492E+03	1.0485E+04	1.4565E+04	1.3517E+04	1.9597E+03	1.2549E+04	1.2255E+04
	Rank	1	2	4	5	7	8	3	9	6
F13	Best	1.4000E+03	1.4237E+03	1.4091E+03	1.4153E+03	1.4653E+03	1.4711E+03	1.4740E+03	1.4426E+03	1.4121E+03
	Mean	1.4056E+03	1.6827E+03	1.4652E+03	6.6774E+03	2.2495E+03	3.4047E+03	1.5324E+03	1.9548E+03	2.2008E+03
	Std	8.0927E+00	5.3475E+02	4.1237E+01	7.5946E+03	1.2199E+03	4.0271E+03	4.2084E+01	7.4434E+02	1.3103E+03
	Rank	1	4	2	9	7	8	3	5	6
F14	Best	1.5002E+03	1.5031E+03	1.5138E+03	1.5318E+03	1.7105E+03	1.6591E+03	1.6815E+03	1.6146E+03	1.5096E+03
	Mean	1.5047E+03	1.8723E+03	1.6978E+03	5.9587E+03	5.8695E+03	4.9736E+03	2.2277E+03	3.4941E+03	2.2402E+03
	Std	4.8891E+00	9.7124E+02	5.3544E+02	5.3195E+03	5.0393E+03	3.8093E+03	4.6001E+02	2.6984E+03	1.1201E+03
	Rank	1	3	2	9	8	7	4	6	5
F15	Best	1.6005E+03	1.6011E+03	1.6006E+03	1.6007E+03	1.6123E+03	1.6034E+03	1.6083E+03	1.6047E+03	1.6017E+03
	Mean	1.6129E+03	1.6967E+03	1.7250E+03	1.7499E+03	1.6889E+03	1.7948E+03	1.6561E+03	1.7609E+03	1.6655E+03
	Std	2.4387E+01	1.0537E+02	1.0414E+02	1.2356E+02	9.3393E+01	1.3418E+02	6.3066E+01	1.0103E+02	8.1236E+01
	Rank	1	5	6	7	4	9	2	8	3
F16	Best	1.7006E+03	1.7030E+03	1.7226E+03	1.7054E+03	1.7335E+03	1.7266E+03	1.7316E+03	1.7300E+03	1.7016E+03
	Mean	1.7175E+03	1.7430E+03	1.7686E+03	1.7579E+03	1.7926E+03	1.7851E+03	1.7557E+03	1.7633E+03	1.7366E+03
	Std	1.2925E+01	2.7849E+01	4.3245E+01	4.7442E+01	4.6760E+01	4.0134E+01	1.3308E+01	2.3608E+01	2.4100E+01
	Rank	1	3	7	5	9	8	4	6	2
F17	Best	1.8004E+03	1.8214E+03	3.0761E+03	2.1102E+03	6.7235E+03	2.3857E+03	3.8406E+03	4.8596E+03	8.2724E+03
	Mean	1.8089E+03	7.0401E+03	2.6025E+04	3.0814E+04	2.7917E+04	2.9458E+04	1.4321E+04	2.1373E+04	2.9249E+04
	Std	9.1926E+00	9.9823E+03	1.6023E+04	1.9249E+04	1.3963E+04	1.3557E+04	7.2371E+03	1.4615E+04	1.2063E+04
	Rank	1	2	5	9	6	8	3	4	7
F18	Best	1.9001E+03	1.9012E+03	1.9027E+03	1.9338E+03	1.9978E+03	1.9730E+03	1.9492E+03	2.0143E+03	1.9039E+03
	Mean	1.9014E+03	3.9873E+03	4.2267E+03	1.2969E+04	1.6045E+04	1.0622E+04	2.8139E+03	4.9575E+03	8.9580E+03
	Std	1.4326E+00	5.5127E+03	4.3312E+03	1.2647E+04	9.5582E+03	9.9016E+03	1.1243E+03	4.5816E+03	7.0951E+03
	Rank	1	3	4	8	9	7	2	5	6
F19	Best	2.0000E+03	2.0050E+03	2.0053E+03	2.0050E+03	2.0225E+03	2.0377E+03	2.0230E+03	2.0245E+03	2.0004E+03
	Mean	2.0092E+03	2.0320E+03	2.0623E+03	2.0359E+03	2.0638E+03	2.1121E+03	2.0425E+03	2.1104E+03	2.0160E+03
	Std	1.0646E+01	1.7704E+01	4.9253E+01	2.5263E+01	3.8850E+01	5.4830E+01	1.2920E+01	6.4250E+01	1.2499E+01
	Rank	1	3	6	4	7	9	5	8	2
F20	Best	2.2000E+03	2.2000E+03	2.2004E+03	2.2027E+03	2.2012E+03	2.2025E+03	2.2031E+03	2.2005E+03	2.2007E+03
	Mean	2.2951E+03	2.3170E+03	2.3197E+03	2.3076E+03	2.3039E+03	2.3261E+03	2.2735E+03	2.2067E+03	2.2564E+03
	Std	3.7582E+01	3.3580E+01	3.3733E+01	4.7849E+01	5.2178E+01	4.3407E+01	5.1597E+01	2.2037E+01	5.7330E+01
	Rank	4	7	8	6	5	9	3	1	2
F21	Best	2.3000E+03	2.3008E+03	2.3012E+03	2.3000E+03	2.2402E+03	2.3013E+03	2.2511E+03	2.2403E+03	2.3010E+03
	Mean	2.3001E+03	2.3025E+03	2.3632E+03	2.3022E+03	2.3732E+03	2.3411E+03	2.3016E+03	2.2993E+03	2.3031E+03
	Std	1.7247E−01	1.2737E+00	2.0125E+02	1.3134E+00	2.7993E+02	2.0183E+02	9.6576E+00	1.5901E+01	1.3148E+00
	Rank	2	5	8	4	9	7	3	1	6
F22	Best	2.6031E+03	2.6085E+03	2.6105E+03	2.6097E+03	2.6140E+03	2.6135E+03	2.6069E+03	2.6139E+03	2.6101E+03
	Mean	2.6119E+03	2.6204E+03	2.6240E+03	2.6231E+03	2.6242E+03	2.6306E+03	2.6164E+03	2.6292E+03	2.6200E+03
	Std	5.0350E+00	7.4125E+00	9.4657E+00	6.9834E+00	6.6024E+00	1.1225E+01	4.5414E+00	8.4811E+00	6.8633E+00
	Rank	1	4	6	5	7	9	2	8	3
F23	Best	2.7317E+03	2.5004E+03	2.7414E+03	2.7398E+03	2.5001E+03	2.5009E+03	2.5214E+03	2.5000E+03	2.5014E+03
	Mean	2.7390E+03	2.7448E+03	2.7587E+03	2.7607E+03	2.7465E+03	2.7480E+03	2.6901E+03	2.6462E+03	2.7431E+03
	Std	3.9986E+00	4.7343E+01	1.0029E+01	1.4095E+01	4.7579E+01	6.8380E+01	8.4535E+01	1.2759E+02	4.6720E+01
	Rank	3	5	8	9	6	7	2	1	4
F24	Best	2.8977E+03	2.8985E+03	2.8980E+03	2.8984E+03	2.8978E+03	2.8991E+03	2.8990E+03	2.8988E+03	2.8978E+03
	Mean	2.9273E+03	2.9245E+03	2.9413E+03	2.9401E+03	2.9368E+03	2.9467E+03	2.9278E+03	2.9350E+03	2.9274E+03
	Std	2.2583E+01	2.4741E+01	3.6399E+01	3.3668E+01	2.9531E+01	4.2640E+01	2.0356E+01	2.1839E+01	2.4010E+01
	Rank	2	1	8	7	6	9	4	5	3
F25	Best	2.9000E+03	2.9000E+03	2.8002E+03	2.9000E+03	2.9000E+03	2.8000E+03	2.8335E+03	2.6001E+03	2.9000E+03
	Mean	2.9032E+03	3.0291E+03	3.0941E+03	3.2396E+03	3.1279E+03	3.1002E+03	2.9229E+03	2.9457E+03	2.9501E+03
	Std	1.1957E+01	1.9114E+02	3.5240E+02	4.3707E+02	3.7141E+02	3.7626E+02	2.6615E+01	1.2609E+02	4.4351E+01
	Rank	1	5	6	9	8	7	2	3	4
F26	Best	3.0890E+03	3.0784E+03	3.0890E+03	3.0880E+03	3.0890E+03	3.0898E+03	3.0908E+03	3.0893E+03	3.0890E+03
	Mean	3.0901E+03	3.0908E+03	3.1007E+03	3.0944E+03	3.0918E+03	3.0956E+03	3.0957E+03	3.0944E+03	3.0969E+03
	Std	1.6432E+00	3.4881E+00	2.0273E+01	1.2550E+01	2.6843E+00	3.4264E+00	1.9211E+00	3.6044E+00	1.7267E+01
	Rank	1	2	9	4	3	6	7	5	8
F27	Best	3.1000E+03	3.1000E+03	3.1025E+03	3.1004E+03	3.1691E+03	3.1643E+03	3.1145E+03	3.1000E+03	3.1000E+03
	Mean	3.2457E+03	3.2560E+03	3.3223E+03	3.3436E+03	3.3713E+03	3.3618E+03	3.1883E+03	3.2349E+03	3.2842E+03
	Std	1.4244E+02	7.9209E+01	1.2023E+02	1.5655E+02	1.7861E+02	8.8264E+01	4.5353E+01	1.1212E+02	1.2119E+02
	Rank	3	4	6	7	9	8	1	2	5
F28	Best	3.1293E+03	3.1342E+03	3.1411E+03	3.1372E+03	3.1396E+03	3.1388E+03	3.1482E+03	3.1505E+03	3.1327E+03
	Mean	3.1404E+03	3.1842E+03	3.2297E+03	3.2365E+03	3.2307E+03	3.2582E+03	3.1920E+03	3.2229E+03	3.1909E+03
	Std	1.0094E+01	5.0117E+01	6.4164E+01	7.4800E+01	6.5920E+01	8.6973E+01	2.1988E+01	5.0169E+01	5.1204E+01
	Rank	1	2	6	8	7	9	4	5	3
F29	Best	3.4600E+03	3.2667E+03	5.7074E+03	5.0654E+03	8.2037E+03	3.9065E+03	6.5876E+03	1.2329E+04	5.2648E+03
	Mean	2.3768E+05	5.6184E+04	6.4584E+05	3.7430E+05	1.6850E+05	5.4212E+05	2.1454E+05	5.4490E+05	3.8659E+05
	Std	4.4337E+05	1.3969E+05	5.2116E+05	4.5765E+05	3.6875E+05	6.4624E+05	3.8785E+05	5.7543E+05	5.4219E+05
	Rank	4	1	9	5	2	7	3	8	6

**Table A10:** Comparison results between BWSMA and SMA variants based on CEC2018 (30D).

No.	Index	BWSMA	PSMADE	MSSMA	ESMA	LSMA	AOSMA	EOSMA	ISMA	DTSMA
F1	Best	1.0241E+02	1.0000E+02	1.7291E+02	3.7327E+03	1.0320E+04	1.2304E+05	3.5973E+08	1.2542E+05	5.7404E+03
	Mean	3.5430E+03	1.4118E+07	1.2998E+05	1.6695E+04	5.0382E+04	3.1874E+06	1.2881E+09	1.3853E+06	4.5185E+07
	Std	4.2661E+03	3.7591E+07	4.7172E+05	8.5435E+03	2.8901E+04	5.5201E+06	5.3013E+08	1.3097E+06	8.7864E+07
	Rank	1	7	4	2	3	6	9	5	8
F2	Best	3.0103E+02	3.0000E+02	2.8664E+04	2.3037E+04	3.3479E+04	4.4410E+04	4.8077E+04	3.0546E+04	2.4655E+04
	Mean	3.5108E+02	4.8235E+04	4.3406E+04	5.0769E+04	5.7363E+04	6.8079E+04	9.1421E+04	4.4395E+04	5.2740E+04
	Std	6.5321E+01	6.1524E+04	7.8098E+03	1.4251E+04	1.5002E+04	1.3491E+04	1.9067E+04	6.8378E+03	1.7852E+04
	Rank	1	4	2	5	7	8	9	3	6
F3	Best	4.4193E+02	4.0000E+02	4.5994E+02	4.6514E+02	4.5151E+02	4.9948E+02	5.5809E+02	4.9150E+02	4.6726E+02
	Mean	4.9308E+02	4.5967E+02	5.1794E+02	5.0221E+02	5.1506E+02	5.5624E+02	6.4188E+02	5.3694E+02	5.1650E+02
	Std	1.6638E+01	7.4924E+01	3.6029E+01	1.6206E+01	3.3691E+01	4.7844E+01	4.8388E+01	3.0625E+01	2.2938E+01
	Rank	2	1	6	3	4	8	9	7	5
F4	Best	5.2105E+02	5.7263E+02	5.9859E+02	5.6560E+02	5.8556E+02	6.2068E+02	6.1724E+02	6.4157E+02	5.5880E+02
	Mean	5.3446E+02	6.4223E+02	6.7198E+02	6.2131E+02	6.3729E+02	7.1174E+02	6.7363E+02	7.1666E+02	6.0262E+02
	Std	9.8776E+00	6.3477E+01	4.0051E+01	2.9501E+01	3.4407E+01	5.1709E+01	2.3317E+01	3.5392E+01	2.4976E+01
	Rank	1	5	6	3	4	8	7	9	2
F5	Best	6.0009E+02	6.0791E+02	6.1292E+02	6.0294E+02	6.0794E+02	6.3444E+02	6.0773E+02	6.3228E+02	6.0467E+02
	Mean	6.0027E+02	6.2241E+02	6.2577E+02	6.0798E+02	6.2798E+02	6.5342E+02	6.1455E+02	6.5279E+02	6.1451E+02
	Std	7.7242E−02	1.1111E+01	8.8767E+00	4.9515E+00	9.8583E+00	9.7114E+00	3.4375E+00	9.1784E+00	5.0759E+00
	Rank	1	5	6	2	7	9	4	8	3
F6	Best	7.4556E+02	8.2562E+02	8.7381E+02	8.2215E+02	8.1802E+02	9.2856E+02	8.9876E+02	8.8149E+02	8.0496E+02
	Mean	7.6442E+02	9.3173E+02	9.9274E+02	8.6649E+02	8.7752E+02	1.0956E+03	9.4882E+02	1.0810E+03	8.8302E+02
	Std	1.0946E+01	5.5169E+01	7.8436E+01	3.1701E+01	4.2533E+01	9.4316E+01	2.8888E+01	9.1748E+01	3.7495E+01
	Rank	1	5	7	2	3	9	6	8	4
F7	Best	8.1328E+02	8.6169E+02	8.8482E+02	8.6285E+02	8.6865E+02	9.2066E+02	9.2705E+02	8.9276E+02	8.7115E+02
	Mean	8.3911E+02	9.3280E+02	9.6105E+02	9.2403E+02	9.2335E+02	9.8956E+02	9.6043E+02	9.5771E+02	9.1814E+02
	Std	1.3461E+01	3.8781E+01	3.8919E+01	2.8858E+01	2.9913E+01	3.4721E+01	1.8896E+01	3.3429E+01	2.3699E+01
	Rank	1	5	8	4	3	9	7	6	2
F8	Best	9.0020E+02	1.6277E+03	2.5918E+03	1.2613E+03	2.1267E+03	3.6763E+03	1.1939E+03	2.7916E+03	1.3675E+03
	Mean	9.0142E+02	4.2393E+03	4.1371E+03	3.4255E+03	4.4393E+03	5.9129E+03	1.9597E+03	5.0284E+03	3.5924E+03
	Std	1.3383E+00	2.3427E+03	8.3651E+02	1.3889E+03	1.5165E+03	1.2604E+03	8.0695E+02	1.2445E+03	1.2773E+03
	Rank	1	6	5	3	7	9	2	8	4
F9	Best	3.1575E+03	3.8546E+03	3.3938E+03	3.3154E+03	3.5730E+03	4.3268E+03	7.1904E+03	4.0389E+03	3.8054E+03
	Mean	4.2796E+03	5.4032E+03	4.5987E+03	4.6209E+03	5.0531E+03	5.5795E+03	8.1447E+03	5.2110E+03	5.4042E+03
	Std	6.4497E+02	7.7695E+02	6.5881E+02	7.5897E+02	5.6597E+02	8.6252E+02	5.2737E+02	5.9170E+02	8.5662E+02
	Rank	1	6	2	3	4	8	9	5	7
F10	Best	1.1099E+03	1.1796E+03	1.1773E+03	1.1493E+03	1.2155E+03	1.2433E+03	1.4725E+03	1.2276E+03	1.1713E+03
	Mean	1.1536E+03	1.4494E+03	1.2612E+03	1.2524E+03	1.3757E+03	1.4130E+03	2.2375E+03	1.3180E+03	1.3519E+03
	Std	3.6894E+01	2.7676E+02	4.8352E+01	5.5387E+01	7.5120E+01	1.0104E+02	3.8970E+02	6.0377E+01	7.6869E+01
	Rank	1	8	3	2	6	7	9	4	5
F11	Best	8.5647E+03	1.9807E+03	8.4931E+05	7.2331E+04	8.3585E+05	3.4579E+06	2.5360E+06	2.7654E+06	2.7002E+05
	Mean	3.0646E+04	3.6501E+06	4.9991E+06	3.8483E+06	1.7322E+07	2.1958E+07	2.7577E+07	2.3192E+07	5.3178E+06
	Std	2.4784E+04	1.1853E+07	3.0312E+06	2.3742E+06	1.6666E+07	1.9430E+07	2.1720E+07	1.7543E+07	6.0905E+06
	Rank	1	2	4	3	6	7	9	8	5
F12	Best	1.7429E+03	2.6599E+03	5.0559E+03	2.6558E+03	6.5428E+04	4.9793E+04	1.0877E+05	1.9101E+04	4.7654E+03
	Mean	3.1906E+03	8.1774E+04	3.2921E+04	2.9090E+04	2.8900E+05	3.3489E+05	6.3503E+05	1.8810E+05	3.4571E+04
	Std	9.1225E+02	3.1742E+05	2.9239E+04	3.1265E+04	1.6906E+05	2.9559E+05	7.0519E+05	1.2780E+05	2.9324E+04
	Rank	1	5	3	2	7	8	9	6	4
F13	Best	1.4351E+03	1.5261E+03	2.2473E+03	2.4087E+04	3.7078E+04	2.2516E+04	3.8881E+03	7.2448E+03	7.9603E+03
	Mean	1.4590E+03	5.9915E+04	2.4842E+05	5.3696E+05	1.5930E+05	6.6267E+05	6.1935E+04	1.6982E+05	8.4231E+04
	Std	1.5078E+01	1.5248E+05	2.5042E+05	7.7928E+05	1.1421E+05	8.3978E+05	4.6318E+04	1.8260E+05	8.5102E+04
	Rank	1	2	7	8	5	9	3	6	4
F14	Best	1.5974E+03	1.8174E+03	1.8245E+03	2.4421E+03	2.7453E+04	1.5066E+04	1.4090E+04	8.1183E+03	1.7036E+03
	Mean	1.7426E+03	1.6312E+04	1.2053E+04	1.7751E+04	1.2018E+05	8.9923E+04	1.0210E+05	6.2507E+04	1.4969E+04
	Std	1.0656E+02	1.4071E+04	1.2954E+04	1.4528E+04	6.2277E+04	4.2838E+04	1.3378E+05	4.7386E+04	1.4640E+04
	Rank	1	4	2	5	9	7	8	6	3
F15	Best	1.6057E+03	2.1962E+03	2.0153E+03	2.1159E+03	2.1554E+03	2.2986E+03	2.2915E+03	2.2115E+03	1.7616E+03
	Mean	2.0826E+03	2.7379E+03	2.8356E+03	2.7355E+03	2.8494E+03	3.1349E+03	2.9765E+03	3.2118E+03	2.4722E+03
	Std	2.6252E+02	3.4771E+02	3.1194E+02	3.2624E+02	3.8787E+02	4.0943E+02	3.0070E+02	3.6609E+02	4.0025E+02
	Rank	1	4	5	3	6	8	7	9	2
F16	Best	1.7596E+03	1.8194E+03	1.7941E+03	2.0875E+03	1.8798E+03	2.1020E+03	1.9196E+03	2.0644E+03	1.8731E+03
	Mean	1.8777E+03	2.2901E+03	2.4482E+03	2.4729E+03	2.3369E+03	2.6026E+03	2.0996E+03	2.4454E+03	2.2179E+03
	Std	1.0153E+02	2.6634E+02	2.9442E+02	1.9341E+02	2.2396E+02	2.9935E+02	1.2709E+02	2.3559E+02	1.7630E+02
	Rank	1	4	7	8	5	9	2	6	3
F17	Best	1.8850E+03	6.5967E+03	3.1736E+05	2.6581E+05	3.9312E+05	2.8630E+05	3.3235E+05	6.7536E+04	1.8968E+05
	Mean	1.9892E+03	2.4335E+06	3.4410E+06	3.3765E+06	3.2823E+06	4.0981E+06	1.2317E+06	1.7889E+06	2.2733E+06
	Std	6.9781E+01	5.5680E+06	4.3506E+06	3.0887E+06	3.1294E+06	4.2853E+06	8.9160E+05	1.9863E+06	2.2462E+06
	Rank	1	5	8	7	6	9	2	3	4
F18	Best	1.9250E+03	2.1552E+03	1.9932E+03	2.0785E+03	2.4984E+04	8.1859E+03	1.4645E+04	2.9067E+04	2.1916E+03
	Mean	1.9746E+03	2.6974E+04	1.1809E+04	2.1619E+04	2.8949E+05	1.0086E+05	2.5905E+05	4.9970E+05	2.2184E+04
	Std	3.3756E+01	2.1763E+04	1.4207E+04	2.2347E+04	1.4936E+05	1.0037E+05	2.8063E+05	4.2058E+05	1.9196E+04
	Rank	1	5	2	3	8	6	7	9	4
F19	Best	2.0303E+03	2.2112E+03	2.1115E+03	2.1977E+03	2.3453E+03	2.4294E+03	2.2385E+03	2.2954E+03	2.1049E+03
	Mean	2.1999E+03	2.5440E+03	2.4647E+03	2.5567E+03	2.7181E+03	2.7463E+03	2.5622E+03	2.5784E+03	2.3865E+03
	Std	1.3211E+02	2.2853E+02	2.0716E+02	2.5932E+02	1.9639E+02	1.4144E+02	1.8271E+02	1.7645E+02	1.9170E+02
	Rank	1	4	3	5	8	9	6	7	2
F20	Best	2.3225E+03	2.3640E+03	2.3980E+03	2.3699E+03	2.3606E+03	2.4222E+03	2.4234E+03	2.3930E+03	2.3727E+03
	Mean	2.3367E+03	2.4514E+03	2.4622E+03	2.4239E+03	2.4290E+03	2.5139E+03	2.4608E+03	2.4813E+03	2.4145E+03
	Std	1.1600E+01	6.9749E+01	4.5525E+01	3.2081E+01	3.4988E+01	5.0959E+01	1.7872E+01	4.2456E+01	3.1120E+01
	Rank	1	5	7	3	4	9	6	8	2
F21	Best	2.3003E+03	2.3000E+03	2.3038E+03	2.3025E+03	2.3063E+03	2.3083E+03	2.4109E+03	2.3130E+03	2.3146E+03
	Mean	4.1865E+03	5.9580E+03	5.0575E+03	5.6692E+03	6.3206E+03	6.7003E+03	2.8344E+03	2.4912E+03	6.0817E+03
	Std	1.6751E+03	1.6452E+03	1.8966E+03	1.0780E+03	1.5673E+03	1.3190E+03	1.2660E+03	8.9335E+02	1.7492E+03
	Rank	3	6	4	5	8	9	2	1	7
F22	Best	2.6735E+03	2.7140E+03	2.7212E+03	2.7163E+03	2.7145E+03	2.7902E+03	2.7633E+03	2.7835E+03	2.7045E+03
	Mean	2.6919E+03	2.7709E+03	2.7931E+03	2.7648E+03	2.7682E+03	2.8755E+03	2.8118E+03	2.8748E+03	2.7585E+03
	Std	1.0484E+01	4.6940E+01	3.8668E+01	2.3102E+01	2.7202E+01	6.4277E+01	2.5633E+01	6.4406E+01	2.8925E+01
	Rank	1	5	6	3	4	9	7	8	2
F23	Best	2.8324E+03	2.8691E+03	2.9088E+03	2.8752E+03	2.8681E+03	2.9288E+03	2.9489E+03	2.9202E+03	2.8973E+03
	Mean	2.8571E+03	2.9401E+03	2.9753E+03	2.9444E+03	2.9328E+03	3.0065E+03	2.9861E+03	3.0176E+03	2.9193E+03
	Std	1.4884E+01	6.4039E+01	4.4717E+01	3.8130E+01	3.2989E+01	5.2661E+01	2.1654E+01	5.6275E+01	1.5390E+01
	Rank	1	4	6	5	3	8	7	9	2
F24	Best	2.8870E+03	2.8760E+03	2.8852E+03	2.8840E+03	2.8920E+03	2.9007E+03	2.9622E+03	2.9091E+03	2.8848E+03
	Mean	2.8907E+03	2.9097E+03	2.9185E+03	2.8960E+03	2.9229E+03	2.9484E+03	3.0280E+03	2.9506E+03	2.9243E+03
	Std	1.0557E+01	3.4297E+01	2.8471E+01	1.0110E+01	2.1556E+01	2.8941E+01	3.9173E+01	2.7926E+01	2.9201E+01
	Rank	1	3	4	2	5	7	9	8	6
F25	Best	2.9014E+03	4.4571E+03	3.3839E+03	2.9019E+03	2.9175E+03	2.8931E+03	3.7856E+03	2.8275E+03	4.4050E+03
	Mean	3.9298E+03	5.3702E+03	5.3353E+03	4.8042E+03	5.0892E+03	6.0703E+03	5.2000E+03	4.5094E+03	4.8686E+03
	Std	2.3423E+02	7.8087E+02	6.9976E+02	7.1782E+02	4.9934E+02	1.0926E+03	6.2731E+02	1.6966E+03	2.4936E+02
	Rank	1	8	7	3	5	9	6	2	4
F26	Best	3.1903E+03	3.1699E+03	3.2141E+03	3.2131E+03	3.2073E+03	3.2227E+03	3.2498E+03	3.2204E+03	3.2024E+03
	Mean	3.2092E+03	3.2029E+03	3.2400E+03	3.2319E+03	3.2373E+03	3.2563E+03	3.2923E+03	3.2731E+03	3.2257E+03
	Std	1.1246E+01	2.5796E+01	1.7800E+01	1.3820E+01	1.8337E+01	1.8603E+01	2.1097E+01	4.1984E+01	1.6433E+01
	Rank	2	1	6	4	5	7	9	8	3
F27	Best	3.1515E+03	3.1000E+03	3.2243E+03	3.2149E+03	3.2374E+03	3.2482E+03	3.3422E+03	3.2359E+03	3.2179E+03
	Mean	3.2208E+03	3.2945E+03	3.2798E+03	3.2797E+03	3.3135E+03	3.3366E+03	3.4381E+03	3.3154E+03	3.3196E+03
	Std	2.4966E+01	1.6891E+02	3.6831E+01	5.3500E+01	5.7544E+01	6.6068E+01	5.9463E+01	3.6098E+01	9.5669E+01
	Rank	1	4	3	2	5	8	9	6	7
F28	Best	3.3168E+03	3.5729E+03	3.6311E+03	3.6183E+03	3.7617E+03	3.7044E+03	3.7077E+03	3.6549E+03	3.5798E+03
	Mean	3.4787E+03	4.1508E+03	4.1094E+03	4.0179E+03	4.2156E+03	4.4999E+03	4.0290E+03	4.3329E+03	3.9628E+03
	Std	1.1094E+02	3.2398E+02	2.2662E+02	2.0908E+02	2.8726E+02	4.5512E+02	2.0377E+02	2.9657E+02	1.9952E+02
	Rank	1	6	5	3	7	9	4	8	2
F29	Best	6.5387E+03	3.5199E+03	1.0919E+04	9.4444E+03	1.6955E+05	2.0461E+05	5.1950E+05	4.9837E+05	7.7834E+03
	Mean	9.2945E+03	4.5718E+04	3.1062E+04	2.7255E+04	2.2564E+06	1.8785E+06	2.9361E+06	4.5101E+06	4.7543E+04
	Std	1.9576E+03	7.1343E+04	2.0563E+04	2.1276E+04	1.5693E+06	2.1123E+06	1.6713E+06	3.4826E+06	6.4662E+04
	Rank	1	4	3	2	7	6	8	9	5

**Table A11:** Comparison results between BWSMA and SMA variants based on CEC2018 (50D).

No.	Index	BWSMA	PSMADE	MSSMA	ESMA	LSMA	AOSMA	EOSMA	ISMA	DTSMA
F1	Best	2.2772E+04	1.0000E+02	6.4899E+05	9.7295E+05	3.9392E+06	9.5649E+07	5.6239E+09	5.7046E+07	3.4340E+07
	Mean	2.8243E+05	6.1813E+08	4.5348E+08	2.4241E+06	1.9712E+07	3.6576E+08	9.6131E+09	2.1697E+08	1.6302E+09
	Std	3.9156E+05	1.3707E+09	6.8866E+08	1.1989E+06	1.3938E+07	2.2345E+08	2.4211E+09	1.3587E+08	1.5894E+09
	Rank	1	7	6	2	3	5	9	4	8
F2	Best	4.8335E+03	3.0000E+02	8.7522E+04	1.0773E+05	1.3557E+05	1.4008E+05	1.3961E+05	1.0639E+05	1.0150E+05
	Mean	1.2313E+04	2.1724E+04	1.2955E+05	1.9671E+05	2.1779E+05	2.4652E+05	2.1285E+05	1.4603E+05	1.8305E+05
	Std	5.5277E+03	7.1891E+04	2.7665E+04	8.3683E+04	6.2447E+04	6.5210E+04	4.3165E+04	2.7059E+04	3.9778E+04
	Rank	1	2	3	6	8	9	7	4	5
F3	Best	4.7970E+02	4.0000E+02	5.3376E+02	5.1636E+02	5.8210E+02	6.4686E+02	1.1197E+03	5.9973E+02	5.9039E+02
	Mean	5.6915E+02	5.8963E+02	6.9837E+02	6.3937E+02	6.8473E+02	8.1523E+02	1.6784E+03	7.4700E+02	7.0552E+02
	Std	4.6928E+01	3.2269E+02	9.5195E+01	8.2290E+01	8.0461E+01	1.0539E+02	3.3899E+02	9.5846E+01	9.0605E+01
	Rank	1	2	5	3	4	8	9	7	6
F4	Best	5.4801E+02	6.9568E+02	7.1428E+02	6.7726E+02	7.0535E+02	7.7916E+02	8.1320E+02	7.7826E+02	6.8301E+02
	Mean	5.7665E+02	8.6683E+02	8.4565E+02	7.7474E+02	8.2337E+02	8.8183E+02	8.8336E+02	8.6257E+02	7.8430E+02
	Std	1.8467E+01	8.2701E+01	5.1042E+01	4.9038E+01	5.8738E+01	4.5495E+01	3.9031E+01	4.5313E+01	4.5274E+01
	Rank	1	7	5	2	4	8	9	6	3
F5	Best	6.0118E+02	6.3126E+02	6.3528E+02	6.1203E+02	6.2703E+02	6.4602E+02	6.1869E+02	6.5129E+02	6.1841E+02
	Mean	6.0176E+02	6.5209E+02	6.5763E+02	6.3189E+02	6.5783E+02	6.6525E+02	6.2796E+02	6.6608E+02	6.3405E+02
	Std	4.0912E−01	1.2810E+01	7.0569E+00	1.3147E+01	1.3764E+01	9.6085E+00	4.5632E+00	5.9429E+00	8.6927E+00
	Rank	1	5	6	3	7	8	2	9	4
F6	Best	8.1345E+02	1.0202E+03	1.1952E+03	9.8396E+02	1.0587E+03	1.4611E+03	1.1407E+03	1.2768E+03	1.0032E+03
	Mean	8.3261E+02	1.3281E+03	1.3932E+03	1.0710E+03	1.1928E+03	1.6114E+03	1.2732E+03	1.5255E+03	1.1562E+03
	Std	1.3712E+01	1.9399E+02	1.2780E+02	5.8507E+01	6.9656E+01	9.0108E+01	5.0025E+01	1.2564E+02	7.8902E+01
	Rank	1	6	7	2	4	9	5	8	3
F7	Best	8.5389E+02	9.8108E+02	9.6875E+02	9.7108E+02	9.8655E+02	1.0898E+03	1.1298E+03	1.1131E+03	9.9292E+02
	Mean	8.8293E+02	1.1569E+03	1.0984E+03	1.0608E+03	1.0799E+03	1.1762E+03	1.1809E+03	1.1857E+03	1.0817E+03
	Std	1.6990E+01	1.2745E+02	5.5483E+01	4.2841E+01	4.7072E+01	4.0532E+01	3.6644E+01	4.8019E+01	5.4665E+01
	Rank	1	6	5	2	3	7	8	9	4
F8	Best	9.1646E+02	7.3420E+03	9.8470E+03	6.7604E+03	5.7581E+03	1.1029E+04	4.8197E+03	9.2286E+03	4.5803E+03
	Mean	9.8237E+02	1.2779E+04	1.3742E+04	1.3700E+04	1.5300E+04	1.7266E+04	8.6381E+03	1.5002E+04	1.5044E+04
	Std	6.5657E+01	4.2815E+03	2.3205E+03	3.4261E+03	4.6562E+03	3.5880E+03	2.5935E+03	2.8783E+03	6.4447E+03
	Rank	1	3	5	4	8	9	2	6	7
F9	Best	5.4030E+03	7.6646E+03	5.8646E+03	5.3024E+03	6.9870E+03	7.4566E+03	1.2738E+04	7.0330E+03	7.6102E+03
	Mean	7.0530E+03	9.0861E+03	7.7637E+03	7.7454E+03	9.2959E+03	9.1503E+03	1.4042E+04	9.3203E+03	1.0048E+04
	Std	8.3965E+02	8.0896E+02	8.3191E+02	1.0840E+03	9.4611E+02	9.5531E+02	6.9658E+02	1.1188E+03	1.3253E+03
	Rank	1	4	3	2	6	5	9	7	8
F10	Best	1.1788E+03	1.2681E+03	1.3106E+03	1.2641E+03	1.5093E+03	1.5648E+03	3.9085E+03	1.6562E+03	1.3758E+03
	Mean	1.2613E+03	1.5997E+03	1.6696E+03	1.6011E+03	1.7681E+03	1.9905E+03	7.6993E+03	2.3330E+03	1.7888E+03
	Std	4.9045E+01	7.2125E+02	5.4318E+02	3.6943E+02	1.5322E+02	2.8048E+02	2.3595E+03	4.2746E+02	6.0474E+02
	Rank	1	2	4	3	5	7	9	8	6
F11	Best	2.6165E+05	4.8274E+03	5.6123E+06	4.9412E+06	3.1661E+07	1.4728E+07	2.3931E+08	1.1518E+07	7.1583E+06
	Mean	1.5527E+06	2.9104E+07	2.4402E+07	3.1679E+07	1.3788E+08	1.8082E+08	4.8420E+08	1.5218E+08	5.9298E+07
	Std	9.5160E+05	6.6964E+07	1.2898E+07	1.7887E+07	6.2919E+07	1.0877E+08	2.1731E+08	1.0985E+08	4.3923E+07
	Rank	1	3	2	4	6	8	9	7	5
F12	Best	9.5964E+03	3.1327E+03	6.3253E+03	2.0611E+04	9.1026E+04	8.9658E+04	2.7505E+06	4.7423E+04	1.6261E+04
	Mean	2.2395E+04	4.3151E+04	2.9145E+04	5.0479E+04	3.4285E+05	3.2943E+05	1.7669E+07	2.3177E+05	9.3725E+04
	Std	7.7615E+03	9.6662E+04	1.8915E+04	1.5178E+04	1.7265E+05	2.0768E+05	1.3889E+07	2.4851E+05	5.6176E+04
	Rank	1	3	2	4	8	7	9	6	5
F13	Best	1.5033E+03	2.0889E+03	1.9077E+05	2.3245E+05	4.1731E+04	1.1731E+05	3.9002E+04	9.8721E+04	6.2884E+04
	Mean	1.6526E+03	2.9368E+05	1.6980E+06	1.6337E+06	7.4726E+05	1.9352E+06	5.5138E+05	9.9456E+05	6.2524E+05
	Std	7.8456E+01	5.4349E+05	1.7153E+06	1.3047E+06	4.9908E+05	2.0747E+06	4.4313E+05	7.8930E+05	3.9331E+05
	Rank	1	2	8	7	5	9	3	6	4
F14	Best	2.5115E+03	1.9923E+03	2.4372E+03	3.0474E+03	4.4306E+04	5.2545E+04	7.9464E+04	9.0954E+03	3.7593E+03
	Mean	3.1552E+03	2.3014E+04	1.6238E+04	1.8430E+04	1.9472E+05	1.7005E+05	4.4899E+05	7.3543E+04	2.5794E+04
	Std	6.6451E+02	2.8203E+04	9.0873E+03	1.0653E+04	1.2988E+05	8.2724E+04	4.8514E+05	9.6703E+04	1.2565E+04
	Rank	1	4	2	3	8	7	9	6	5
F15	Best	1.7799E+03	2.2160E+03	2.8406E+03	2.6805E+03	2.5063E+03	3.2678E+03	3.2432E+03	3.1045E+03	2.5820E+03
	Mean	2.8279E+03	3.8730E+03	3.7318E+03	3.5517E+03	3.9490E+03	4.2687E+03	3.9910E+03	4.2396E+03	3.5876E+03
	Std	4.5854E+02	6.1011E+02	4.6396E+02	4.3651E+02	5.1307E+02	5.0142E+02	3.4916E+02	5.6080E+02	4.3924E+02
	Rank	1	5	4	2	6	9	7	8	3
F16	Best	2.1039E+03	3.0801E+03	2.9636E+03	2.6659E+03	3.1485E+03	3.1694E+03	2.7991E+03	2.9940E+03	2.7311E+03
	Mean	2.5797E+03	3.7260E+03	3.4508E+03	3.3790E+03	3.7855E+03	3.9485E+03	3.5078E+03	3.6257E+03	3.3087E+03
	Std	2.5402E+02	4.3092E+02	2.6435E+02	3.2551E+02	3.6903E+02	3.9901E+02	2.8821E+02	3.3162E+02	3.1031E+02
	Rank	1	7	4	3	8	9	5	6	2
F17	Best	2.4931E+03	5.7230E+03	5.0978E+05	6.3527E+05	5.5984E+05	9.3466E+05	1.5501E+06	1.1075E+06	5.7002E+05
	Mean	6.6545E+03	1.4355E+06	7.1096E+06	5.8980E+06	7.1864E+06	8.5324E+06	6.1690E+06	5.7511E+06	5.8071E+06
	Std	5.3557E+03	3.2474E+06	6.0555E+06	3.8228E+06	5.4004E+06	6.0432E+06	3.5232E+06	6.9422E+06	3.9372E+06
	Rank	1	2	7	5	8	9	6	3	4
F18	Best	1.9961E+03	2.2001E+03	2.2033E+03	2.5872E+03	1.7702E+05	2.1330E+05	1.3118E+04	3.3468E+05	2.2445E+03
	Mean	2.4372E+03	1.5373E+04	2.4267E+04	1.9118E+04	1.4870E+06	9.0219E+05	6.5246E+05	2.5440E+06	1.9678E+04
	Std	7.1108E+02	1.5870E+04	1.5578E+04	1.5900E+04	8.5286E+05	6.7015E+05	4.9698E+05	1.8325E+06	1.7534E+04
	Rank	1	2	5	3	8	7	6	9	4
F19	Best	2.1923E+03	2.3610E+03	2.5608E+03	2.3493E+03	2.7197E+03	2.5954E+03	2.9493E+03	2.7084E+03	2.4565E+03
	Mean	2.7642E+03	3.4931E+03	3.2342E+03	3.1603E+03	3.5204E+03	3.6650E+03	3.5330E+03	3.2753E+03	3.2692E+03
	Std	3.0558E+02	3.8923E+02	3.2802E+02	3.4727E+02	3.2343E+02	3.4552E+02	2.7198E+02	2.7457E+02	2.9840E+02
	Rank	1	6	3	2	7	9	8	5	4
F20	Best	2.3525E+03	2.4628E+03	2.5003E+03	2.4647E+03	2.4897E+03	2.6142E+03	2.5620E+03	2.5834E+03	2.4656E+03
	Mean	2.3792E+03	2.7182E+03	2.6108E+03	2.5588E+03	2.5829E+03	2.7551E+03	2.6545E+03	2.7188E+03	2.5445E+03
	Std	1.5952E+01	1.7438E+02	6.5267E+01	5.4364E+01	5.5821E+01	7.1946E+01	3.6505E+01	7.4931E+01	4.2963E+01
	Rank	1	7	5	3	4	9	6	8	2
F21	Best	5.8954E+03	7.9284E+03	2.3225E+03	7.6005E+03	8.0607E+03	8.4443E+03	1.3962E+04	2.5834E+03	1.0015E+04
	Mean	8.2923E+03	1.0139E+04	9.2732E+03	9.3368E+03	1.0376E+04	1.0890E+04	1.5541E+04	1.0491E+04	1.1635E+04
	Std	9.2514E+02	7.8962E+02	1.6194E+03	9.3280E+02	1.2507E+03	1.1995E+03	7.1557E+02	2.2906E+03	1.1931E+03
	Rank	1	4	2	3	5	7	9	6	8
F22	Best	2.7643E+03	2.9340E+03	2.9430E+03	2.9190E+03	2.9364E+03	3.0835E+03	3.0353E+03	3.0611E+03	2.9154E+03
	Mean	2.8093E+03	3.1147E+03	3.0742E+03	3.0007E+03	3.0245E+03	3.2612E+03	3.1254E+03	3.2536E+03	2.9857E+03
	Std	2.3901E+01	1.6809E+02	6.0556E+01	4.8584E+01	4.6460E+01	1.2045E+02	3.2962E+01	9.9453E+01	4.4996E+01
	Rank	1	6	5	3	4	9	7	8	2
F23	Best	2.9373E+03	3.0184E+03	3.0966E+03	3.0755E+03	3.0720E+03	3.2100E+03	3.1916E+03	3.1287E+03	3.0444E+03
	Mean	2.9723E+03	3.1773E+03	3.2383E+03	3.1713E+03	3.1390E+03	3.3821E+03	3.2632E+03	3.3169E+03	3.1376E+03
	Std	1.6964E+01	1.0223E+02	6.3556E+01	6.2760E+01	4.6589E+01	9.4779E+01	3.7262E+01	9.9917E+01	5.2025E+01
	Rank	1	5	6	4	3	9	7	8	2
F24	Best	2.9855E+03	2.9312E+03	3.0572E+03	3.0519E+03	3.0675E+03	3.1051E+03	3.5453E+03	3.1440E+03	3.1005E+03
	Mean	3.0412E+03	2.9676E+03	3.2137E+03	3.1071E+03	3.1506E+03	3.2843E+03	4.1380E+03	3.2235E+03	3.2335E+03
	Std	2.2587E+01	1.2279E+02	9.6798E+01	3.6783E+01	4.0343E+01	6.7984E+01	3.7539E+02	5.8671E+01	1.1355E+02
	Rank	2	1	5	3	4	8	9	6	7
F25	Best	4.1717E+03	6.0278E+03	3.8480E+03	2.9401E+03	3.3680E+03	4.5733E+03	6.5525E+03	4.0205E+03	5.2928E+03
	Mean	4.5449E+03	8.0511E+03	8.2157E+03	6.1177E+03	6.1024E+03	9.7460E+03	7.8129E+03	7.5498E+03	6.4015E+03
	Std	2.3611E+02	1.5523E+03	2.1005E+03	1.8223E+03	2.0601E+03	1.5762E+03	5.7347E+02	3.2412E+03	4.5993E+02
	Rank	1	7	8	3	2	9	6	5	4
F26	Best	3.2264E+03	3.2000E+03	3.4081E+03	3.3502E+03	3.3825E+03	3.3675E+03	3.6584E+03	3.5170E+03	3.3002E+03
	Mean	3.3094E+03	3.2497E+03	3.6312E+03	3.4914E+03	3.5325E+03	3.7199E+03	3.8608E+03	3.7184E+03	3.5019E+03
	Std	3.8880E+01	1.3086E+02	1.2887E+02	9.5240E+01	1.0043E+02	1.6699E+02	1.1246E+02	1.6586E+02	8.4692E+01
	Rank	2	1	6	3	5	8	9	7	4
F27	Best	3.2632E+03	3.3000E+03	3.3887E+03	3.3090E+03	3.3401E+03	3.5230E+03	4.2240E+03	3.4343E+03	3.6218E+03
	Mean	3.3027E+03	3.6494E+03	3.7291E+03	3.4022E+03	3.7394E+03	3.9086E+03	4.7918E+03	3.6985E+03	4.5933E+03
	Std	3.1206E+01	9.2299E+02	5.6153E+02	8.2011E+01	4.4994E+02	5.0080E+02	3.3160E+02	1.2370E+02	5.4456E+02
	Rank	1	3	5	2	6	7	9	4	8
F28	Best	3.3828E+03	4.0942E+03	3.6990E+03	3.7354E+03	4.4689E+03	4.8256E+03	4.5305E+03	4.8457E+03	3.8868E+03
	Mean	3.8790E+03	5.4609E+03	4.7477E+03	4.6392E+03	5.5211E+03	6.3663E+03	5.0799E+03	6.3122E+03	4.8225E+03
	Std	2.2118E+02	8.6846E+02	3.9770E+02	4.1482E+02	5.1188E+02	6.8571E+02	3.0028E+02	6.7974E+02	5.2890E+02
	Rank	1	6	3	2	7	9	5	8	4
F29	Best	1.0807E+06	3.7388E+03	1.1910E+06	1.6414E+06	2.6098E+07	1.5959E+07	4.0173E+07	2.8447E+07	4.1697E+06
	Mean	2.5866E+06	4.2287E+06	2.8195E+06	2.9428E+06	5.0468E+07	6.5650E+07	9.1566E+07	1.0305E+08	1.2572E+07
	Std	9.6265E+05	9.1032E+06	1.1761E+06	7.7206E+05	1.9286E+07	2.3721E+07	2.5710E+07	4.1232E+07	5.1729E+06
	Rank	1	4	2	3	6	7	8	9	5

**Table A12:** Comparison results between BWSMA and SMA variants based on CEC2018 (100D).

No.	Index	BWSMA	PSMADE	MSSMA	ESMA	LSMA	AOSMA	EOSMA	ISMA	DTSMA
F1	Best	1.0265E+08	1.0000E+02	1.0273E+10	1.5980E+08	1.3914E+09	5.7963E+09	6.0312E+10	5.0720E+09	1.9181E+10
	Mean	2.8491E+08	2.0209E+09	2.8975E+10	2.2008E+08	4.5267E+09	1.3254E+10	8.7886E+10	1.0330E+10	3.8529E+10
	Std	1.7408E+08	1.1069E+10	1.1025E+10	3.5040E+07	1.5496E+09	3.7959E+09	1.3888E+10	2.8149E+09	1.2755E+10
	Rank	2	3	7	1	4	6	9	5	8
F2	Best	1.3769E+05	6.5811E+02	2.4868E+05	3.1837E+05	3.2539E+05	3.3116E+05	3.6328E+05	2.9425E+05	4.4900E+05
	Mean	1.6598E+05	6.2645E+04	3.1088E+05	9.5370E+05	7.9412E+05	5.9109E+05	5.4570E+05	3.4426E+05	7.4169E+05
	Std	2.2104E+04	2.0461E+05	5.8454E+04	2.7790E+05	3.4020E+05	3.1668E+05	1.0958E+05	2.8032E+04	1.3130E+05
	Rank	2	1	3	9	8	6	5	4	7
F3	Best	6.9066E+02	4.0000E+02	1.1313E+03	7.1488E+02	1.0305E+03	1.5504E+03	8.0323E+03	1.3348E+03	1.3791E+03
	Mean	8.3589E+02	6.8358E+02	1.9135E+03	9.2868E+02	1.4377E+03	2.5573E+03	1.0049E+04	2.0374E+03	2.3361E+03
	Std	6.2124E+01	1.1038E+03	6.5387E+02	9.6152E+01	1.9733E+02	4.7336E+02	1.3399E+03	4.3262E+02	6.8833E+02
	Rank	2	1	5	3	4	8	9	6	7
F4	Best	7.2204E+02	1.2034E+03	1.2513E+03	1.0907E+03	1.2478E+03	1.3443E+03	1.4119E+03	1.2258E+03	1.1609E+03
	Mean	8.0340E+02	1.3592E+03	1.3776E+03	1.2785E+03	1.4234E+03	1.4986E+03	1.5635E+03	1.4727E+03	1.3270E+03
	Std	3.9544E+01	1.5183E+02	7.0398E+01	1.0696E+02	8.2376E+01	6.0082E+01	6.5691E+01	7.2246E+01	1.0034E+02
	Rank	1	4	5	2	6	8	9	7	3
F5	Best	6.1384E+02	6.4660E+02	6.5098E+02	6.4089E+02	6.5884E+02	6.6770E+02	6.4678E+02	6.6969E+02	6.5472E+02
	Mean	6.1915E+02	6.6098E+02	6.6499E+02	6.5353E+02	6.7448E+02	6.7724E+02	6.5588E+02	6.7855E+02	6.6524E+02
	Std	3.2956E+00	7.6064E+00	4.2144E+00	6.6013E+00	5.6399E+00	5.3401E+00	5.3264E+00	5.2588E+00	6.7805E+00
	Rank	1	4	5	2	7	8	3	9	6
F6	Best	1.2112E+03	2.1898E+03	2.5445E+03	1.7037E+03	1.9892E+03	2.6188E+03	2.4914E+03	2.5839E+03	2.1128E+03
	Mean	1.3225E+03	2.9305E+03	2.8889E+03	2.0733E+03	2.4867E+03	3.1360E+03	2.6646E+03	3.1600E+03	2.4525E+03
	Std	6.0837E+01	3.9502E+02	1.8954E+02	1.6539E+02	2.6303E+02	1.9145E+02	1.2601E+02	2.1020E+02	2.0140E+02
	Rank	1	7	6	2	4	8	5	9	3
F7	Best	1.0303E+03	1.4547E+03	1.5244E+03	1.3885E+03	1.5666E+03	1.7462E+03	1.7250E+03	1.7466E+03	1.3715E+03
	Mean	1.1138E+03	1.7599E+03	1.7277E+03	1.6059E+03	1.7476E+03	1.9288E+03	1.8680E+03	1.8976E+03	1.6628E+03
	Std	4.4101E+01	2.2933E+02	9.5766E+01	1.2007E+02	1.0755E+02	8.8628E+01	7.1663E+01	6.5942E+01	1.1775E+02
	Rank	1	6	4	2	5	9	7	8	3
F8	Best	3.3200E+03	1.7656E+04	2.3437E+04	2.2879E+04	2.9949E+04	2.7167E+04	3.3780E+04	2.7446E+04	3.0431E+04
	Mean	5.6313E+03	2.5263E+04	2.9508E+04	3.3904E+04	3.5627E+04	3.6056E+04	4.5519E+04	3.7223E+04	5.0494E+04
	Std	1.9177E+03	6.3680E+03	4.1699E+03	4.5039E+03	3.7481E+03	6.9673E+03	7.0502E+03	4.6684E+03	1.2132E+04
	Rank	1	2	3	4	5	6	8	7	9
F9	Best	1.4404E+04	1.4789E+04	1.3664E+04	1.3776E+04	1.7255E+04	1.4275E+04	2.9286E+04	1.6856E+04	1.8778E+04
	Mean	1.7008E+04	1.7120E+04	1.5993E+04	1.6928E+04	2.0754E+04	1.9953E+04	3.0990E+04	2.0679E+04	2.3748E+04
	Std	1.6548E+03	1.6567E+03	1.2731E+03	1.5835E+03	1.8778E+03	2.0016E+03	8.1299E+02	1.6560E+03	2.5491E+03
	Rank	3	4	1	2	7	5	9	6	8
F10	Best	3.2800E+03	2.3545E+03	1.9234E+04	1.9824E+04	3.6450E+04	4.5393E+04	1.1277E+05	3.5420E+04	1.8560E+04
	Mean	4.5120E+03	1.2215E+04	4.6116E+04	6.2465E+04	7.3751E+04	1.3203E+05	1.5850E+05	9.0541E+04	7.2622E+04
	Std	8.6733E+02	3.1913E+04	1.3927E+04	3.1548E+04	1.8775E+04	4.3091E+04	2.6668E+04	2.8683E+04	2.7561E+04
	Rank	1	2	3	4	6	8	9	7	5
F11	Best	4.3706E+07	1.5220E+04	1.0893E+08	7.9872E+07	3.3278E+08	3.9815E+08	6.6482E+09	5.9159E+08	4.9303E+08
	Mean	9.5046E+07	1.5894E+08	6.4656E+08	2.5477E+08	9.2137E+08	1.5528E+09	1.1253E+10	1.2719E+09	1.7100E+09
	Std	3.5906E+07	8.6647E+08	8.7260E+08	8.8376E+07	3.7032E+08	6.6314E+08	2.8981E+09	5.2978E+08	9.5708E+08
	Rank	1	2	4	3	5	7	9	6	8
F12	Best	3.2287E+04	7.3629E+03	2.3195E+04	4.5849E+04	8.7732E+04	1.9407E+05	7.2208E+07	1.4726E+05	7.9464E+04
	Mean	6.1914E+04	5.3463E+05	2.5788E+05	2.5030E+06	5.7856E+06	5.3851E+06	4.6223E+08	6.0345E+05	1.5233E+07
	Std	1.5329E+04	2.8460E+06	7.9590E+05	6.3380E+06	1.4225E+07	2.2779E+07	2.5845E+08	4.2061E+05	2.3430E+07
	Rank	1	3	2	5	7	6	9	4	8
F13	Best	6.3099E+03	2.0865E+03	1.0861E+06	1.6450E+06	9.1892E+05	1.3205E+06	3.2904E+06	2.7567E+06	8.4568E+05
	Mean	2.1032E+04	3.7529E+05	4.4620E+06	5.1977E+06	6.8331E+06	7.1228E+06	1.0192E+07	5.3935E+06	5.9499E+06
	Std	1.2511E+04	1.0311E+06	2.1301E+06	3.0286E+06	3.2925E+06	3.6973E+06	5.2513E+06	2.0616E+06	3.6656E+06
	Rank	1	2	3	4	7	8	9	5	6
F14	Best	8.1804E+03	1.7682E+03	5.9453E+03	1.1694E+04	3.6463E+04	5.7567E+04	8.4992E+06	2.8868E+04	3.7769E+04
	Mean	2.6324E+04	2.2146E+05	6.6900E+04	7.9369E+05	1.6305E+06	7.6887E+05	2.8000E+07	5.1332E+05	3.6294E+05
	Std	1.1801E+04	1.1548E+06	1.9317E+05	1.6329E+06	2.4890E+06	3.4224E+06	1.4665E+07	2.4034E+06	1.4097E+06
	Rank	1	3	2	7	8	6	9	5	4
F15	Best	4.0879E+03	5.2223E+03	4.7470E+03	4.5316E+03	6.2277E+03	6.8853E+03	7.9990E+03	6.9859E+03	4.6261E+03
	Mean	4.9977E+03	6.1325E+03	6.3646E+03	6.2083E+03	7.4944E+03	8.5775E+03	8.9878E+03	8.9101E+03	6.4175E+03
	Std	7.4905E+02	6.5246E+02	7.6658E+02	7.3010E+02	7.9277E+02	8.5704E+02	5.5377E+02	1.3104E+03	8.4104E+02
	Rank	1	2	4	3	6	7	9	8	5
F16	Best	3.4256E+03	4.7067E+03	5.0189E+03	4.6056E+03	5.4948E+03	6.0705E+03	5.6278E+03	5.3169E+03	4.5023E+03
	Mean	4.6606E+03	6.5558E+03	6.1219E+03	5.5962E+03	6.4246E+03	7.4520E+03	6.4340E+03	6.4170E+03	5.8027E+03
	Std	5.0956E+02	9.5349E+02	5.8425E+02	6.1499E+02	6.0518E+02	7.4471E+02	4.6567E+02	5.7613E+02	6.5486E+02
	Rank	1	8	4	2	6	9	7	5	3
F17	Best	4.7477E+04	1.5871E+04	1.8163E+06	3.0645E+06	2.4824E+06	1.8904E+06	3.6818E+06	2.1546E+06	1.3697E+06
	Mean	1.1397E+05	4.0302E+05	8.7971E+06	1.1373E+07	1.1956E+07	1.2483E+07	1.3980E+07	6.8897E+06	9.0052E+06
	Std	2.9092E+04	1.2995E+06	6.1019E+06	5.9041E+06	6.4682E+06	6.3501E+06	7.2678E+06	4.2311E+06	6.1256E+06
	Rank	1	2	4	6	7	8	9	3	5
F18	Best	5.5765E+03	2.9290E+03	3.8233E+03	8.7634E+03	5.0790E+06	2.7242E+06	1.3736E+07	1.6232E+06	6.3781E+04
	Mean	3.4841E+04	1.6243E+05	2.0678E+04	1.1120E+05	1.4690E+07	1.4412E+07	4.4835E+07	1.5173E+07	1.9102E+06
	Std	5.1133E+04	8.0569E+05	1.3779E+04	1.7466E+05	5.4649E+06	6.9257E+06	2.2778E+07	9.3678E+06	3.6679E+06
	Rank	2	4	1	3	7	6	9	8	5
F19	Best	3.5877E+03	4.3344E+03	4.2840E+03	4.3189E+03	5.0748E+03	4.3897E+03	6.2242E+03	4.6176E+03	4.8461E+03
	Mean	4.7847E+03	5.7490E+03	5.3724E+03	5.4802E+03	5.9994E+03	6.0352E+03	7.1144E+03	5.6219E+03	5.9657E+03
	Std	5.5316E+02	6.8522E+02	5.0957E+02	5.8964E+02	6.6859E+02	5.8573E+02	3.9074E+02	4.8114E+02	6.3589E+02
	Rank	1	5	2	3	7	8	9	4	6
F20	Best	2.5332E+03	2.8945E+03	2.9976E+03	3.0009E+03	3.0305E+03	3.3541E+03	3.2557E+03	3.3003E+03	2.9346E+03
	Mean	2.6101E+03	3.2775E+03	3.2069E+03	3.1535E+03	3.2390E+03	3.6939E+03	3.3702E+03	3.6660E+03	3.1476E+03
	Std	4.1714E+01	2.9818E+02	1.0983E+02	1.1098E+02	1.4544E+02	1.7412E+02	7.0034E+01	2.2008E+02	1.0797E+02
	Rank	1	6	4	3	5	9	7	8	2
F21	Best	1.6957E+04	1.5881E+04	1.7543E+04	1.6381E+04	1.9203E+04	1.8058E+04	3.1234E+04	2.0840E+04	2.0508E+04
	Mean	1.9600E+04	1.8953E+04	1.9742E+04	1.9304E+04	2.2661E+04	2.2189E+04	3.3638E+04	2.4358E+04	2.5564E+04
	Std	1.7835E+03	1.6938E+03	1.3845E+03	1.3837E+03	1.6355E+03	1.6066E+03	1.1045E+03	1.9306E+03	2.7386E+03
	Rank	3	1	4	2	6	5	9	7	8
F22	Best	3.0796E+03	3.4184E+03	3.3219E+03	3.2563E+03	3.3552E+03	3.8151E+03	3.8254E+03	3.6516E+03	3.3939E+03
	Mean	3.1773E+03	3.7474E+03	3.5402E+03	3.4040E+03	3.5996E+03	4.0840E+03	4.0013E+03	4.1253E+03	3.5173E+03
	Std	4.9991E+01	3.0734E+02	9.1065E+01	8.9650E+01	1.2057E+02	1.8547E+02	8.7463E+01	1.9708E+02	1.1281E+02
	Rank	1	6	4	2	5	8	7	9	3
F23	Best	3.5310E+03	4.0138E+03	3.9474E+03	3.7837E+03	3.8917E+03	4.3896E+03	4.4619E+03	4.3582E+03	3.7723E+03
	Mean	3.6141E+03	4.4434E+03	4.3264E+03	4.0578E+03	4.1328E+03	4.8687E+03	4.7062E+03	4.8274E+03	4.0445E+03
	Std	4.8296E+01	3.0220E+02	1.7642E+02	1.4371E+02	1.5046E+02	2.0576E+02	1.3066E+02	2.8974E+02	1.5119E+02
	Rank	1	6	5	3	4	9	7	8	2
F24	Best	3.4140E+03	3.1342E+03	4.0962E+03	3.4387E+03	3.9387E+03	4.3573E+03	8.0506E+03	4.2950E+03	5.1241E+03
	Mean	3.5994E+03	3.3619E+03	4.5878E+03	3.6223E+03	4.3451E+03	4.8973E+03	9.5649E+03	4.6657E+03	5.9846E+03
	Std	7.7347E+01	7.5070E+02	3.5875E+02	8.5393E+01	1.8920E+02	3.0510E+02	1.0302E+03	2.3573E+02	7.0328E+02
	Rank	2	1	5	3	4	7	9	6	8
F25	Best	7.9404E+03	1.2056E+04	1.6024E+04	5.0144E+03	1.3366E+04	1.7083E+04	1.6903E+04	8.2631E+03	1.1857E+04
	Mean	9.3106E+03	1.8961E+04	1.9438E+04	1.4145E+04	1.6818E+04	2.3963E+04	2.2209E+04	2.2351E+04	1.4484E+04
	Std	5.2458E+02	3.5047E+03	2.4147E+03	2.8477E+03	2.0762E+03	3.8437E+03	2.6574E+03	5.0108E+03	1.3520E+03
	Rank	1	5	6	2	4	9	7	8	3
F26	Best	3.4009E+03	3.2000E+03	3.5941E+03	3.4927E+03	3.6195E+03	3.8200E+03	4.4311E+03	3.6766E+03	3.5763E+03
	Mean	3.5339E+03	3.3148E+03	3.7556E+03	3.6478E+03	3.8269E+03	4.2150E+03	4.7317E+03	4.0241E+03	3.7398E+03
	Std	5.6082E+01	2.2273E+02	1.0393E+02	7.8908E+01	1.1259E+02	2.2145E+02	2.1257E+02	2.5743E+02	1.1569E+02
	Rank	2	1	5	3	6	8	9	7	4
F27	Best	3.5590E+03	3.2325E+03	3.9333E+03	3.5705E+03	4.0311E+03	4.4549E+03	9.4426E+03	4.3866E+03	5.9944E+03
	Mean	3.6806E+03	3.4971E+03	4.3474E+03	3.7210E+03	4.6672E+03	5.5903E+03	1.2459E+04	5.2687E+03	1.0331E+04
	Std	9.5163E+01	1.0766E+03	2.7440E+02	1.1732E+02	3.9560E+02	5.5962E+02	1.3068E+03	4.4948E+02	2.8753E+03
	Rank	2	1	4	3	5	7	9	6	8
F28	Best	5.5167E+03	6.5066E+03	6.9109E+03	6.4502E+03	8.6281E+03	9.4831E+03	9.6880E+03	9.0513E+03	7.1650E+03
	Mean	6.6937E+03	9.2483E+03	8.0587E+03	7.4525E+03	1.0005E+04	1.1937E+04	1.0410E+04	1.0798E+04	8.8109E+03
	Std	6.2841E+02	1.5798E+03	6.3647E+02	5.0935E+02	8.4343E+02	1.2488E+03	5.5799E+02	9.9748E+02	8.0167E+02
	Rank	1	5	3	2	6	9	7	8	4
F29	Best	3.2499E+05	3.9667E+03	4.4536E+05	5.5091E+05	4.0327E+07	4.3498E+07	1.1863E+08	8.4034E+07	5.3455E+06
	Mean	1.1447E+06	9.8737E+05	2.9147E+06	1.7574E+06	1.4516E+08	1.9708E+08	3.0956E+08	2.7029E+08	4.0235E+07
	Std	5.1175E+05	5.3558E+06	2.7549E+06	9.2568E+05	7.1101E+07	1.1442E+08	1.0674E+08	1.6778E+08	2.8711E+07
	Rank	2	1	4	3	6	7	9	8	5

**Table A13:** Comparison results between BWSMA and improved algorithms based on CEC2022 (10D).

No.	Index	BWSMA	NMPA	BEESO	FDBARO	WOSSA	PHISAO	ESLPSO	EPSCA	IGWO
F1	Best	3.0000E+02	3.0965E+02	1.1153E+03	6.9410E+02	3.0251E+02	3.4055E+02	3.0003E+02	3.0000E+02	3.0040E+02
	Mean	3.0000E+02	3.5010E+02	2.4124E+03	2.6451E+03	5.6494E+02	1.2333E+03	3.2768E+02	3.0000E+02	3.0608E+02
	Std	1.3704E−03	3.6816E+01	8.9681E+02	1.2847E+03	4.6474E+02	1.0829E+03	5.3229E+01	3.6367E−13	7.1490E+00
	Rank	2	5	8	9	6	7	4	1	3
F2	Best	4.0002E+02	4.0048E+02	4.0505E+02	4.0084E+02	4.0003E+02	4.0003E+02	4.0005E+02	4.0000E+02	4.0004E+02
	Mean	4.0649E+02	4.0597E+02	4.0788E+02	4.0713E+02	4.1378E+02	4.1382E+02	4.0867E+02	4.0590E+02	4.0425E+02
	Std	2.8829E+00	4.0267E+00	1.1353E+00	5.3986E+00	2.1786E+01	1.8793E+01	7.2028E+00	2.9487E+00	3.0498E+00
	Rank	4	3	6	5	8	9	7	2	1
F3	Best	6.0000E+02	6.0135E+02	6.0002E+02	6.0055E+02	6.0000E+02	6.0000E+02	6.0000E+02	6.0000E+02	6.0009E+02
	Mean	6.0002E+02	6.0440E+02	6.0009E+02	6.0140E+02	6.0425E+02	6.0001E+02	6.0000E+02	6.0019E+02	6.0021E+02
	Std	1.9725E−02	2.3891E+00	6.2868E−02	5.9746E−01	5.9267E+00	3.9089E−02	7.1738E−05	5.9383E−01	6.2669E−02
	Rank	3	9	4	7	8	2	1	5	6
F4	Best	8.0298E+02	8.0657E+02	8.1322E+02	8.0767E+02	8.0995E+02	8.0099E+02	8.0597E+02	8.0497E+02	8.0300E+02
	Mean	8.0813E+02	8.2033E+02	8.2786E+02	8.1619E+02	8.2355E+02	8.0468E+02	8.2902E+02	8.1721E+02	8.1151E+02
	Std	3.4574E+00	6.0268E+00	6.4061E+00	5.0854E+00	7.9996E+00	2.4532E+00	8.9915E+00	1.0183E+01	7.3784E+00
	Rank	2	6	8	4	7	1	9	5	3
F5	Best	9.0000E+02	9.0160E+02	9.0000E+02	9.0035E+02	9.0018E+02	9.0000E+02	9.0000E+02	9.0000E+02	9.0000E+02
	Mean	9.0001E+02	9.2421E+02	9.0015E+02	9.0395E+02	1.2470E+03	9.0011E+02	9.0002E+02	9.1037E+02	9.0004E+02
	Std	2.2714E−02	1.7375E+01	2.2947E−01	4.7900E+00	2.4488E+02	2.8510E−01	8.2948E−02	1.7032E+01	3.1145E−02
	Rank	1	8	5	6	9	4	2	7	3
F6	Best	1.8002E+03	2.2677E+03	1.9239E+03	1.9669E+03	1.9373E+03	1.8279E+03	1.8258E+03	1.8018E+03	2.5279E+03
	Mean	1.8130E+03	4.7254E+03	5.5868E+03	3.8589E+03	3.8391E+03	3.5875E+03	2.4158E+03	1.8374E+03	5.6507E+03
	Std	1.1076E+01	1.9140E+03	2.2316E+03	1.6484E+03	1.9209E+03	1.4286E+03	1.0026E+03	2.1735E+01	2.0008E+03
	Rank	1	7	8	6	5	4	3	2	9
F7	Best	2.0000E+03	2.0077E+03	2.0040E+03	2.0040E+03	2.0020E+03	2.0000E+03	2.0041E+03	2.0010E+03	2.0005E+03
	Mean	2.0163E+03	2.0264E+03	2.0193E+03	2.0199E+03	2.0285E+03	2.0125E+03	2.0212E+03	2.0218E+03	2.0225E+03
	Std	8.6227E+00	7.6143E+00	6.0034E+00	6.1569E+00	2.4754E+01	1.1892E+01	6.1163E+00	6.5941E+00	9.7098E+00
	Rank	2	8	3	4	9	1	5	6	7
F8	Best	2.2002E+03	2.2080E+03	2.2051E+03	2.2063E+03	2.2121E+03	2.2071E+03	2.2108E+03	2.2191E+03	2.2058E+03
	Mean	2.2138E+03	2.2233E+03	2.2246E+03	2.2203E+03	2.2345E+03	2.2209E+03	2.2248E+03	2.2222E+03	2.2249E+03
	Std	9.4827E+00	5.8835E+00	5.3873E+00	5.3409E+00	3.6571E+01	2.8473E+00	5.3055E+00	1.4329E+00	6.5047E+00
	Rank	1	5	6	2	9	3	7	4	8
F9	Best	2.5293E+03	2.5293E+03	2.5293E+03	2.5297E+03	2.5293E+03	2.5293E+03	2.5293E+03	2.5293E+03	2.5293E+03
	Mean	2.5293E+03	2.5298E+03	2.5293E+03	2.5323E+03	2.5293E+03	2.5293E+03	2.5293E+03	2.5295E+03	2.5293E+03
	Std	1.1269E−04	6.5754E−01	5.0892E−05	2.2155E+00	1.8882E−13	1.0924E−03	1.2057E−11	8.9043E−01	1.0193E−02
	Rank	4	8	3	9	1	5	2	7	6
F10	Best	2.5002E+03	2.5005E+03	2.4162E+03	2.5004E+03	2.5003E+03	2.5004E+03	2.5003E+03	2.5003E+03	2.5002E+03
	Mean	2.5225E+03	2.5007E+03	2.5061E+03	2.5045E+03	2.5831E+03	2.5223E+03	2.5384E+03	2.5471E+03	2.5291E+03
	Std	4.5283E+01	1.8549E−01	3.3035E+01	2.1513E+01	1.1410E+02	4.4533E+01	5.4936E+01	5.8024E+01	4.8532E+01
	Rank	5	1	3	2	9	4	7	8	6
F11	Best	2.6000E+03	2.6129E+03	2.6156E+03	2.6269E+03	2.6000E+03	2.6000E+03	2.6000E+03	2.6000E+03	2.6011E+03
	Mean	2.7400E+03	2.6891E+03	2.8666E+03	2.6882E+03	2.8350E+03	2.7460E+03	2.8850E+03	2.8541E+03	2.7923E+03
	Std	1.5222E+02	6.1054E+01	9.5766E+01	3.7680E+01	1.2257E+02	1.0695E+02	6.0354E+01	1.0875E+02	1.4748E+02
	Rank	3	2	8	1	6	4	9	7	5
F12	Best	2.8594E+03	2.8598E+03	2.8614E+03	2.8650E+03	2.8614E+03	2.8626E+03	2.8595E+03	2.8587E+03	2.8587E+03
	Mean	2.8631E+03	2.8639E+03	2.8630E+03	2.8671E+03	2.8652E+03	2.8657E+03	2.8634E+03	2.8636E+03	2.8615E+03
	Std	1.5168E+00	1.3249E+00	1.1777E+00	1.4621E+00	2.0772E+00	3.4210E+00	1.3576E+00	1.7798E+00	1.7010E+00
	Rank	3	6	2	9	7	8	4	5	1

**Table A14:** Comparison results between BWSMA and improved algorithms based on CEC2022 (20D).

No.	Index	BWSMA	NMPA	BEESO	FDBARO	WOSSA	PHISAO	ESLPSO	EPSCA	IGWO
F1	Best	3.0007E+02	1.8039E+03	1.3673E+04	9.0961E+03	5.3098E+03	3.6985E+03	2.6539E+03	3.0000E+02	4.7049E+02
	Mean	3.0044E+02	4.6668E+03	2.2083E+04	2.4648E+04	2.0495E+04	1.6363E+04	8.3584E+03	3.0000E+02	1.2235E+03
	Std	2.9651E−01	1.7709E+03	5.2385E+03	8.2944E+03	9.5228E+03	7.2825E+03	3.8480E+03	2.8731E−04	8.5465E+02
	Rank	2	4	8	9	7	6	5	1	3
F2	Best	4.4490E+02	4.5496E+02	4.4379E+02	4.6732E+02	4.2950E+02	4.4915E+02	4.2809E+02	4.4570E+02	4.4551E+02
	Mean	4.5129E+02	4.9243E+02	4.5352E+02	4.9723E+02	4.5679E+02	4.6030E+02	4.5307E+02	4.5280E+02	4.5206E+02
	Std	7.9738E+00	3.3924E+01	1.0620E+01	1.8108E+01	1.9035E+01	1.4497E+01	1.2623E+01	2.9438E+00	7.4437E+00
	Rank	1	8	5	9	6	7	4	3	2
F3	Best	6.0004E+02	6.1486E+02	6.0031E+02	6.0713E+02	6.1253E+02	6.0001E+02	6.0001E+02	6.0060E+02	6.0049E+02
	Mean	6.0012E+02	6.2573E+02	6.0069E+02	6.1146E+02	6.3642E+02	6.0017E+02	6.0004E+02	6.0505E+02	6.0087E+02
	Std	4.7548E−02	5.9748E+00	2.5291E−01	3.3456E+00	1.4684E+01	1.3581E−01	9.8757E−02	4.2466E+00	2.9917E−01
	Rank	2	8	4	7	9	3	1	6	5
F4	Best	8.0996E+02	8.5589E+02	8.5224E+02	8.4776E+02	8.4179E+02	8.0201E+02	8.1758E+02	8.1293E+02	8.1892E+02
	Mean	8.1862E+02	8.7798E+02	9.0549E+02	8.8757E+02	8.7558E+02	8.1355E+02	8.8026E+02	8.4285E+02	8.6294E+02
	Std	7.9788E+00	1.6972E+01	1.6344E+01	1.8908E+01	1.4255E+01	7.4343E+00	3.3000E+01	2.0627E+01	3.3746E+01
	Rank	2	6	9	8	5	1	7	3	4
F5	Best	9.0000E+02	1.0454E+03	9.0138E+02	9.4548E+02	1.3818E+03	9.0019E+02	9.0000E+02	9.1031E+02	9.0048E+02
	Mean	9.0010E+02	1.5154E+03	9.1601E+02	1.2747E+03	2.2076E+03	9.0433E+02	9.0149E+02	9.9821E+02	9.0330E+02
	Std	1.1172E−01	2.9033E+02	1.3881E+01	1.9099E+02	3.2760E+02	5.2224E+00	2.6049E+00	9.0140E+01	4.0960E+00
	Rank	1	8	5	7	9	4	2	6	3
F6	Best	1.8632E+03	4.6781E+04	3.4373E+03	9.2970E+04	2.0311E+03	1.8916E+03	2.1741E+03	1.8383E+03	5.5286E+03
	Mean	1.9348E+03	2.7734E+05	3.6287E+04	6.5703E+05	4.7017E+03	4.1236E+03	6.6227E+03	1.9371E+03	2.6349E+04
	Std	6.9540E+01	1.8390E+05	3.6673E+04	4.8947E+05	2.8453E+03	1.6139E+03	4.4147E+03	6.2737E+01	2.2103E+04
	Rank	1	8	7	9	4	3	5	2	6
F7	Best	2.0024E+03	2.0476E+03	2.0407E+03	2.0416E+03	2.0493E+03	2.0271E+03	2.0361E+03	2.0337E+03	2.0250E+03
	Mean	2.0253E+03	2.0835E+03	2.0715E+03	2.0735E+03	2.1554E+03	2.0455E+03	2.0731E+03	2.1053E+03	2.0394E+03
	Std	7.2348E+00	2.3341E+01	2.1550E+01	2.0006E+01	9.0132E+01	1.6314E+01	2.1476E+01	4.6183E+01	1.5171E+01
	Rank	1	7	4	6	9	3	5	8	2
F8	Best	2.2082E+03	2.2265E+03	2.2318E+03	2.2275E+03	2.2213E+03	2.2215E+03	2.2290E+03	2.2209E+03	2.2251E+03
	Mean	2.2223E+03	2.2366E+03	2.2440E+03	2.2322E+03	2.2663E+03	2.2237E+03	2.2384E+03	2.2501E+03	2.2321E+03
	Std	2.9836E+00	7.7123E+00	8.2011E+00	4.2814E+00	5.8295E+01	1.5108E+00	6.0786E+00	5.7527E+01	4.0645E+00
	Rank	1	5	7	4	9	2	6	8	3
F9	Best	2.4808E+03	2.4835E+03	2.4808E+03	2.4855E+03	2.4808E+03	2.4808E+03	2.4808E+03	2.4809E+03	2.4809E+03
	Mean	2.4808E+03	2.4890E+03	2.4812E+03	2.4986E+03	2.4808E+03	2.4823E+03	2.4808E+03	2.4853E+03	2.4812E+03
	Std	1.3075E−02	5.7888E+00	2.9200E−01	7.7565E+00	2.1844E−02	4.0684E+00	1.6510E−01	5.1455E+00	1.8047E−01
	Rank	1	8	5	9	2	6	3	7	4
F10	Best	2.5003E+03	2.5006E+03	2.5008E+03	2.5007E+03	2.5009E+03	2.5004E+03	2.5006E+03	2.5006E+03	2.5004E+03
	Mean	2.8591E+03	2.5097E+03	2.6640E+03	2.7481E+03	3.6943E+03	2.6542E+03	3.5838E+03	3.8779E+03	2.7523E+03
	Std	3.7931E+02	3.9295E+01	1.6176E+02	5.0183E+02	7.4353E+02	2.8251E+02	1.7920E+03	9.8056E+02	7.7621E+02
	Rank	6	1	3	4	8	2	7	9	5
F11	Best	2.9001E+03	3.0921E+03	3.1382E+03	3.4002E+03	2.6001E+03	2.9029E+03	2.9001E+03	2.9000E+03	2.9262E+03
	Mean	2.9007E+03	3.5457E+03	3.3026E+03	3.7754E+03	2.9059E+03	2.9347E+03	2.9005E+03	2.9000E+03	2.9484E+03
	Std	3.6095E−01	2.1732E+02	1.0598E+02	2.6842E+02	1.1442E+02	5.0484E+01	2.2818E−01	5.0191E−10	1.4977E+01
	Rank	3	8	7	9	4	5	2	1	6
F12	Best	2.9320E+03	2.9526E+03	2.9411E+03	2.9755E+03	2.9431E+03	2.9417E+03	2.9326E+03	2.9393E+03	2.9325E+03
	Mean	2.9413E+03	2.9770E+03	2.9494E+03	3.0062E+03	2.9811E+03	2.9514E+03	2.9490E+03	2.9581E+03	2.9416E+03
	Std	7.2332E+00	2.0969E+01	8.2173E+00	1.6236E+01	3.9831E+01	7.5426E+00	1.3392E+01	1.2415E+01	5.5385E+00
	Rank	1	7	4	9	8	5	3	6	2
